# AI-Driven Topic Modeling and Sentiment Analysis of Systemic Lupus Erythematosus Discussions on Social Media: Cross-Platform Study

**DOI:** 10.2196/95175

**Published:** 2026-08-31

**Authors:** Jian Tang, Huifang Jiang, Jie Peng, Hao Zhang, Ping Qin, Zikun Huang

**Affiliations:** 1 Department of Pharmacy, Guilin People’s Hospital, Guilin, Guangxi, China; 2 Department of Pharmacy, The Second Affiliated Hospital, Zhejiang University School of Medicine, Hangzhou, Zhejiang, China; 3 Department of Pharmacy, Maternal and Child Health Hospital of Hubei Province, Tongji Medical College, Huazhong University of Science and Technology, Wuhan, Hubei, China; 4 School of Information Engineering, Guilin University, No. 3 Yanzhong Road, Yanshan District, Guilin, Guangxi, 541006, China, 86 17615107081

**Keywords:** systemic lupus erythematosus, social media, AI, sentiment analysis, modeling analysis

## Abstract

**Background:**

Systemic lupus erythematosus (SLE) is a multifactorial autoimmune disease influenced by genetic, epigenetic, ecological, and environmental factors, with a global prevalence of 7.7 to 13 per 100,000, and standardized mortality rates of 2.4% to 5.9%. Between 14% and 75% of patients experience psychiatric comorbidities such as anxiety and depression, which impair treatment adherence and health-related quality of life. Social media has become an important channel for patients to express health concerns and seek support. Reddit and Weibo, as mainstream platforms globally and in China, respectively, host large volumes of user-generated content; however, no prior study has examined SLE-related discourse across both cultural and platform contexts.

**Objective:**

This study aimed to characterize SLE-related discussions on Reddit and Weibo. Specifically, we sought to identify and hierarchically categorize discussion topics, compare platform-specific differences in patient concerns, assess sentiment polarity across topics and thematic groups, and characterize public misconceptions related to SLE.

**Methods:**

This observational, computational content analysis examined all SLE-related discussions on Reddit (r/lupus) and Weibo up to November 30, 2024. An AI-driven pipeline first embedded the discussions using the Multilingual-E5-base BERT (bidirectional encoder representations from transformers) model, then applied uniform manifold approximation and projection (UMAP) for dimensionality reduction and hierarchical density-based spatial clustering of applications with noise (HDBSCAN) for density-based clustering to identify fine-grained topics, with keywords extracted via class-based term frequency-inverse document frequency (c-TF-IDF). Topics were further grouped into higher-level thematic domains through spectral clustering, with labels and definitions generated using GPT-5 Thinking via prompt engineering. Sentiment polarity (positive, neutral, and negative) was classified using a fine-tuned multilingual BERT model. Two clinical pharmacists independently validated the topic modeling and sentiment results.

**Results:**

We analyzed 11,318 discussions from 3649 unique authors on Reddit and 33,628 discussions from 21,509 authors on Weibo. We identified 99 fine-grained topics on Reddit and 189 on Weibo, grouped into 6 thematic domains per platform. Reddit discussions centered on diagnostic journeys, treatment experiences, and symptom management, whereas Weibo discussions emphasized social news, charitable activities, and traditional Chinese medicine (eg, artemisinin). The overall sentiment on Reddit was positive (mean 0.42, SD 0.86; 95% CI 0.40-0.43), whereas that on Weibo was neutral (mean 0.01, SD 0.91; 95% CI 0.00-0.02). Sentiment was positive in 7493, neutral in 1032, and negative in 2793 Reddit discussions, whereas on Weibo, it was positive, neutral, and negative in 14,026, 5768, and 13,834 discussions, respectively. Treatment-related misconceptions and unverified remedies recurred on both platforms.

**Conclusions:**

This cross-platform analysis reveals both shared and platform-specific concerns among people with SLE, highlighting distinct informational and emotional needs across cultural and platform contexts. Recurring treatment-related misconceptions and the substantial volume of negative-sentiment discussions warrant targeted public health communication and psychological support. The findings may help clinicians, public health authorities, and patient support organizations identify unmet needs, while the AI-driven approach offers a scalable, real-time tool for monitoring patient perspectives.

## Introduction

Systemic lupus erythematosus (SLE) is a multifactorial autoimmune disease influenced by genetic, epigenetic, ecological, and environmental factors. It leads to the deposition of immune complexes in tissues, triggering autoimmune cascade reactions that can affect a single organ or multiple systems [1]. Globally, SLE among adults has an estimated prevalence ranging from 7.7 to 13 per 100,000 [2] and standardized mortality rates between 2.4% and 5.9% [3]. In China, the prevalence is reported at 48 per 100,000 [4], whereas in the United States it reaches 72.8 per 100,000 [5]. As one of the most common autoimmune diseases, SLE poses a significant global public health challenge. Among individuals with SLE, 14% to 75% experience psychiatric comorbidities such as insomnia, anxiety disorders, depression, and schizophrenia [6]. These manifestations contribute to poor treatment adherence [7], substantially impair health-related quality of life (HRQoL) [8], and in some cases, lead to suicidal ideation [9]. Many patients turn to social media to share their illness experiences, seek advice, and offer mutual support [10].

Over the past 2 decades, the number of social media users has grown exponentially, with billions of people engaging on these platforms daily—making them an indispensable part of modern life [11]. On such platforms, individuals can easily share personal experiences, understand the emotions of friends and family, and seek emotional support or validation during critical moments. Due to their real-time nature, accessibility, and extensive user base, social media platforms have become valuable tools for public health research. For instance, Golder et al [12] conducted a qualitative analysis of 11,852 social media posts to explore patients’ beliefs and attitudes regarding statin therapy decisions. Bi et al [13] used online health communities (OHCs) to uncover the ongoing needs and challenges faced by patients with albinism, a rare disease in China. Similarly, Blumenthal et al [14] analyzed content from multiple social media platforms to assess the risk of skin and soft tissue infections associated with allergen immunotherapy. To better understand the diverse experiences of breast cancer patients in China, Zhang et al [11] applied machine learning to examine thematic and emotional differences between posts authored by patients and those written by their relatives or friends. Collectively, such studies demonstrate that social media provides valuable insights into how patients and the general public perceive and respond to public health issues beyond traditional clinical settings [15].

Reddit is a centralized online community platform organized into topic-specific subcommunities based on user interests, allowing individuals to engage with content that aligns with their preferences. As of June 30, 2025, Reddit hosts more than 100,000 active subreddits, with more than 110 million daily active users and more than 22 billion posts and comments [16]. This vast volume of user-generated content offers an extensive data source for public health research. Increasingly, researchers have used Reddit data to monitor and analyze diverse health-related topics, including mental health, ophthalmology, opioid use, and breast cancer [17-20].

Weibo, one of the most popular social media platforms in China—often referred to as the “Chinese Twitter”—has approximately 249 million daily active users. It enables individuals to access information, share interests and personal updates, and interact with others [21]. Beyond serving as a convenient channel for patients to obtain health-related information and support, Weibo provides a trusted space where the public can freely express opinions, perspectives, and emotions on a wide range of health topics [22]. Researchers have leveraged Weibo content to predict users’ risk of depression [23] and to identify health-related discussion themes, including public perceptions of health care experiences and patient-prioritized areas requiring attention from health care providers [24].

Given the enormous volume of social media data, manual analysis alone is infeasible. The rapid advancement of AI has already had profound impacts across sectors, such as health care, education, agriculture, and government. As a key subfield of AI, natural language processing (NLP) enables entity relation extraction (ERE) and named entity recognition (NER) from large text corpora, thereby facilitating the analysis and interpretation of massive social media datasets [25]. To the best of our knowledge, no prior study has examined SLE-related discourse across both cultural and platform contexts. To address this knowledge gap, this study applies NLP techniques to analyze Reddit and Weibo posts related to SLE, with the aim of identifying and categorizing major discussion topics, exploring their temporal dynamics, and conducting sentiment analysis. We hypothesized that (1) large-scale social media data from Reddit and Weibo would present heterogeneous semantic structures and user behavior profiles regarding SLE, (2) the AI-driven pipeline would successfully map out structurally distinct thematic groups, capturing unique localized clinical and psychosocial needs across different linguistic contexts, and (3) the assessed sentiment polarities would reflect different emotional landscapes and coping mechanisms between Western and Chinese patient communities.

## Methods

### Ethical Considerations

All data used in this study were obtained from publicly accessible, anonymized sources on Reddit and Weibo. Ethical approval was obtained from the Guilin People’s Hospital Ethics Committee (2025-317KY).

### Data Collection and Preprocessing

Data were collected from Reddit and Weibo up to November 30, 2024. On Reddit, SLE-related communities were identified using the keywords “systemic lupus erythematosus,” “lupus erythematosus,” “lupus,” and “SLE.” Most of the keywords returned only r/lupus, whereas “lupus” returned 15 subreddits; however, all except r/lupus had weekly activity below 100 (mostly in the single digits) and were therefore excluded. All posts and comments from r/lupus were retrieved via the official Reddit API on January 20, 2025. As Weibo has no official API, a web crawler collected SLE-related posts and comments on January 10, 2025, using the Chinese keywords “系统性红斑狼疮” (systemic lupus erythematosus), “红斑狼疮” (lupus erythematosus), and “狼疮” (lupus). “SLE” was not used as a Weibo search term because it retrieved large volumes of unrelated content (eg, referring to regions rather than the disease). Posts deleted by users or removed by the platform before retrieval could not be obtained and were excluded.

Because posts and comments on Weibo cannot be reliably distinguished, posts and comments on both platforms were uniformly treated as individual “discussions” and used as the basic analytical unit to enable cross-platform comparison. The unstructured text was preprocessed using regular expressions to remove emojis, URLs, user mentions (“@”), extraneous whitespace, and stop words; exact duplicate discussions were removed via string matching.

### Modeling

BERTopic [26] (bidirectional encoder representations from transformers) was applied to the preprocessed dataset to identify discussion topics related to SLE. This state-of-the-art NLP method begins by embedding text using the pretrained sentence-level BERT model Multilingual-E5-base. The Multilingual-E5-base model was trained on more than 1 billion text pairs from multilingual datasets, including Reddit and Semantic Scholar Open Research Corpus (S2ORC; a large corpus containing more than 12.8 million scholarly papers in the biomedical domain) [27], making it particularly suitable for analyzing medical discourse expressed in both Chinese and English social media. Detailed methodological and parameter settings are provided in eMethods in Multimedia Appendix 1.

High-dimensional text embeddings were first reduced using uniform manifold approximation and projection (UMAP) to preserve the semantic structure, and then clustered using hierarchical density-based spatial clustering of applications with noise (HDBSCAN), which handles clusters of varying density and assigns points below the minimum cluster-size threshold to a noise class; these noise documents were excluded from subsequent analyses. Representative keywords for each topic were extracted using class-based term frequency-inverse document frequency (c-TF-IDF). A grid search optimized 4 parameters influencing topic quality—the UMAP n_neighbors and n_components, the HDBSCAN min_cluster_size, and the BERTopic nr_topics (Table S1 in Multimedia Appendix 1), selecting the configuration yielding the most coherent and well-separated topics.

Because BERTopic typically generates many granular topics, an additional clustering step was used to improve interpretability. Pairwise semantic similarities computed from the c-TF-IDF representations were reduced via UMAP and grouped into broader thematic groups using spectral clustering, which partitions topics according to the similarity-graph structure and captures nonconvex relationships that conventional methods may miss. This yielded a 2-level structure: fine-grained topics at the document level and higher-order thematic categories for interpretation. The optimal number of thematic groups was determined using the Silhouette coefficient (values approaching 1.0 indicate well-defined partitions [28]) and the Davies-Bouldin index (lower values indicate better clustering [29]). The configuration yielding the most favorable combination of both indices was selected.

To enhance the interpretability of these thematic groups, we used a large language model (LLM) as part of an innovative analytical approach. Specifically, the c-TF-IDF keywords and a representative subset of discussions for each group, both produced by BERTopic, were provided to GPT-5 Thinking (see eMethods in Multimedia Appendix 1 for details) via the following prompt:

- “I have groups that are described by the following keywords: [KEYWORDS]”
- “In these groups, the following documents are a small but representative subset of all documents in the groups: [DOCUMENTS]”
- “Based on the information above, please provide clear, concise names for each group. Write a definition for each group to clearly capture their essence.”

The generated theme names were then reviewed by one of the authors (JT), who revised one label: “Lupus diagnosis and daily struggles” was changed to “Navigating lupus diagnosis and daily struggles” to better capture the active and ongoing nature of patients’ experiences in seeking a diagnosis and managing daily treatment, rather than a static description.

### Sentiment Analysis

Sentiment analysis used bert-base-multilingual-cased [30], a multilingual BERT variant pretrained on 104 languages, including Chinese and English, with reliable performance on multilingual sentiment classification [31]. The model classified SLE-related discussions into 3 categories—positive, neutral, and negative—following the standard sentiment polarity framework [32,33] (operational definitions in Multimedia Appendix 2). It was fine-tuned on 2 public 3-class corpora: a Chinese news sentiment dataset (chinese-news-sentiment-c3-ds, ModelScope) and the English Coronavirus Tweets NLP dataset (Coronavirus tweets NLP-Text Classification, Kaggle); parameter settings are provided in Table S2 in Multimedia Appendix 1. The Softmax layer output positive, neutral, and negative classes, assigned values of 1, 0, and −1, respectively. For each topic and thematic group, the sentiment score was the arithmetic mean across all discussions within it, with values closer to 1, 0, and −1 indicating predominantly positive, neutral, and negative orientations. The model achieved an *F*_1_-score of 88.52% on the combined test set, meeting deployment requirements.

### Manual Validation

All validation was performed by 2 clinical pharmacists (JP and HJ) working independently, with disagreements resolved by author JT. For topic validation, 30% of topics were randomly sampled from each platform (30 from Reddit, 57 from Weibo; 87 in total), with the top 10 c-TF-IDF keywords and 5 posts extracted per topic (435 total posts; Cohen κ=0.79). All 12 higher-level thematic groups (6 Reddit and 6 Weibo) were similarly reviewed, with 25 posts extracted per group (300 total posts; Cohen κ=0.82). For sentiment validation, 300 discussions were randomly selected (25 per thematic group); interrater agreement was 0.76 (Cohen κ), and manual-model agreement was 79.2%. These findings support the reliability of the sentiment classification, though proportions should be interpreted as general trends rather than precise estimates.

## Results

**Figure 1.**
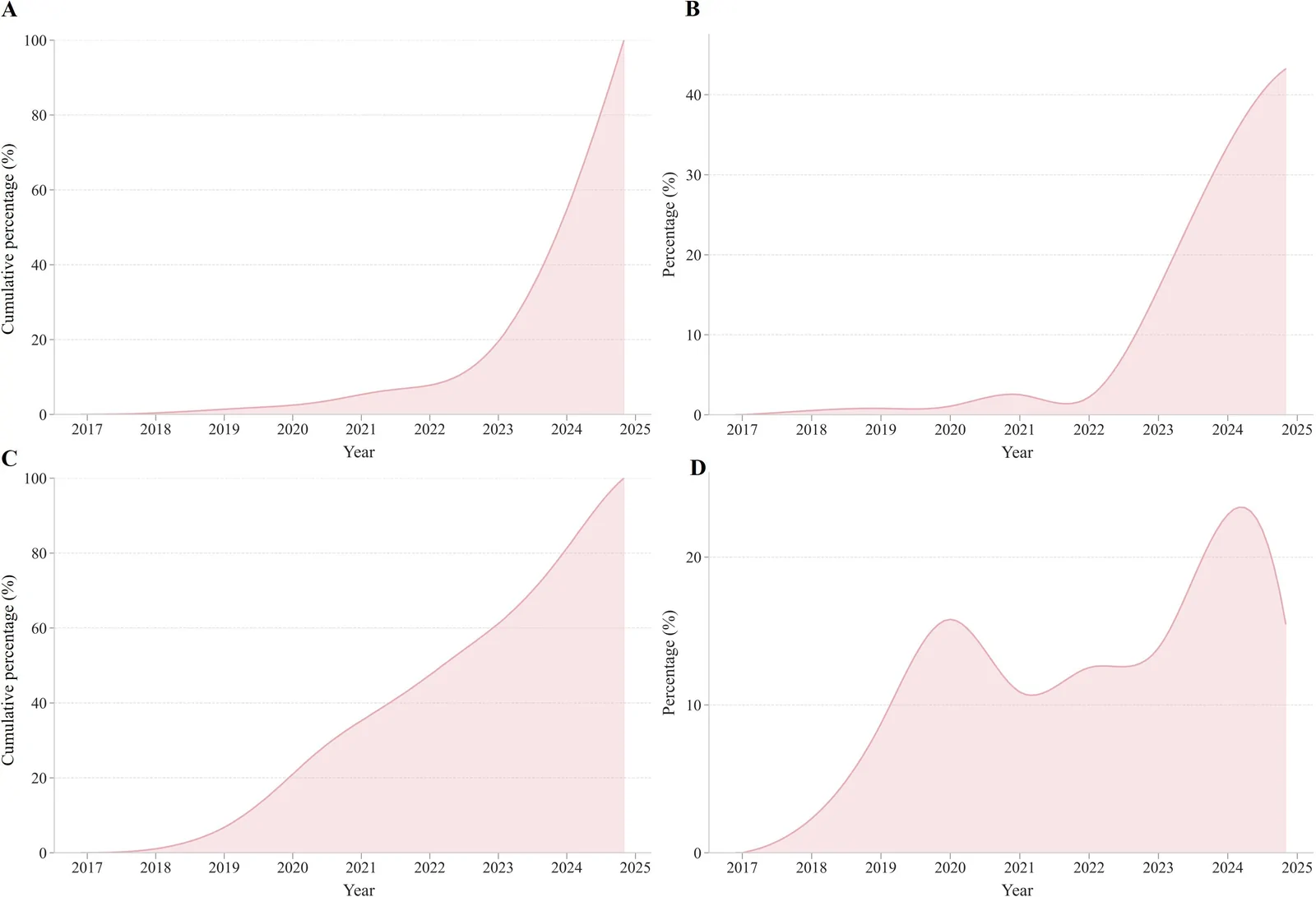
Temporal distribution of lupus-related discussions on Reddit and Weibo. (A, C) Number of discussions over time on Reddit (A) and Weibo (C). (B, D) Percentage of discussions per year on Reddit (B) and Weibo (D).

On Reddit, we identified one SLE-related subreddit (r/lupus), from which 11,318 discussions pertaining to SLE were retrieved, contributed by 3649 unique authors. The average number of characters per discussion was 535.51 (SD 760.83). Notably, 92.55% (n=3377) of authors contributed between 1 and 5 discussions (Figure S1 in Multimedia Appendix 1). On Weibo, a total of 33,628 SLE-related discussions were collected from 21,509 unique authors. The average number of characters per discussion was 265.38 (SD 440.18), with 97.75% (n=21,025) of authors contributing between 1 and 5 discussions (Figure S1 in Multimedia Appendix 1). The annual variation in the number of SLE-related discussions over time is presented in Figure 1.

Using the silhouette coefficient, Calinski-Harabasz index, and Davies-Bouldin index (Figure S2 in Multimedia Appendix 1), we identified 99 SLE-related topics from Reddit and 189 from Weibo (Table S3 in Multimedia Appendix 1). On Reddit, the most prevalent topics included discussions of antinuclear antibody (ANA) positivity and SLE symptoms (topic 0), expressions of gratitude and emotional support (topic 1), and experiences related to malar rash diagnosis, skin biopsy, and clinical visits (topic 2). Other frequently discussed themes involved the management of medication side effects (topics 6, 7, and 87), occupational challenges and coping strategies among individuals with SLE (topics 88 and 93), and mental health impacts (topic 91).

On Weibo, the most prominent topics focused on breakthroughs in the use of artemisinin for SLE treatment (topic 0), family caregiving and emotional struggles (topic 1), and the daily life experiences and emotional states of individuals with SLE (topic 2). Additional themes addressed hormone therapies and related side effects (topic 3), fear and hope in relation to SLE (topic 5), and charity fundraising and mutual support initiatives (topic 8).

**Figure 2.**
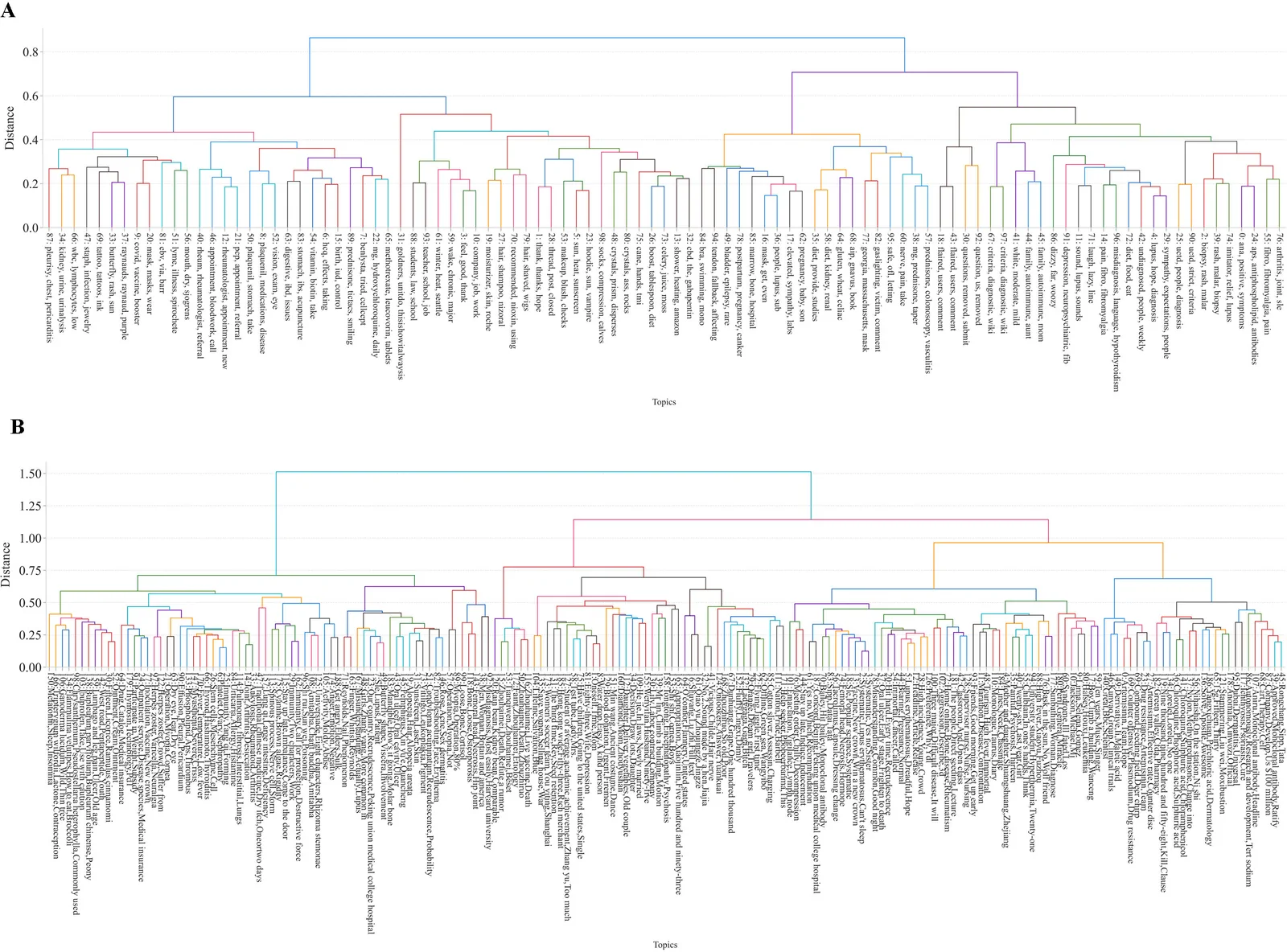
Hierarchical clustering of discussion topics on Reddit (A) and Weibo (B). Trees were constructed based on pairwise semantic similarities between topic representations; each leaf represents one topic, with color indicating the thematic group and size proportional to its number of discussions. Topics that merge lower are more semantically similar; the y-axis represents the linkage distance.

Overall, discussions on both Reddit and Weibo encompassed clinical manifestations, diagnostic processes, pharmacological treatments, side effect management, patient life experiences, and emotional support. However, Reddit discussions tended to emphasize the prolonged diagnostic journey and detailed treatment experiences, whereas Weibo posts focused more on social news, charitable activities, and the potential role of traditional Chinese medicine (TCM)—particularly artemisinin—in SLE management. The hierarchical structure of these topics is shown in Figure 2, while the temporal trends in topic-specific discussion volumes are presented in Figure S3 in Multimedia Appendix 1.

**Figure 3.**
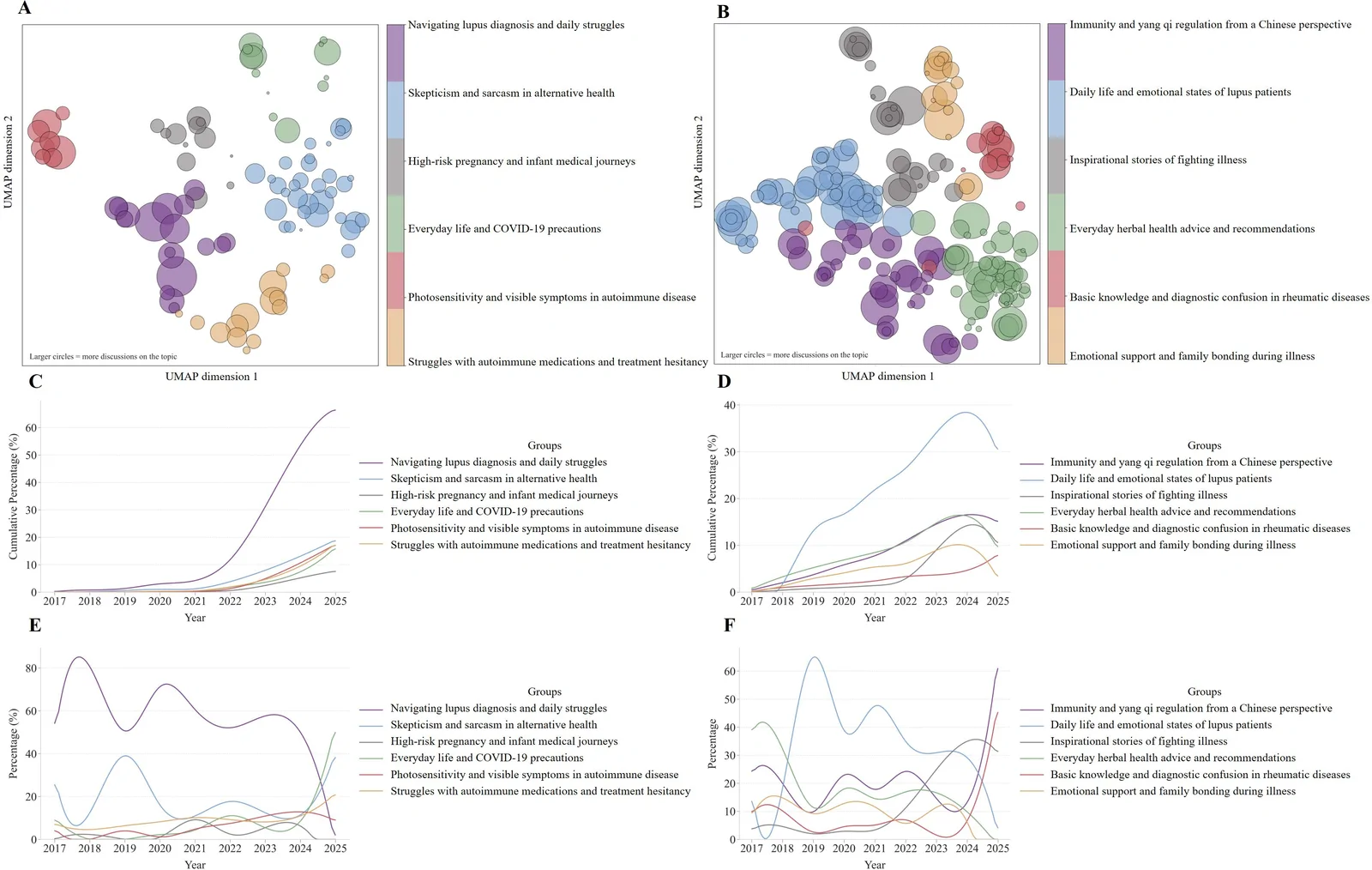
Thematic group visualization and temporal evolution of lupus-related discussions on Reddit and Weibo. (A, B) Uniform manifold approximation and projection visualizations of BERTopic-derived topics from Reddit (A) and Weibo (B); each circle represents one topic, with size proportional to its number of discussions and color indicating the thematic group. (C, D) Cumulative proportion of discussions per thematic group over time for Reddit (C) and Weibo (D). (E, F) Annual proportion of discussions per thematic group for Reddit (E) and Weibo (F).

Following sensitivity analyses using the silhouette coefficient and the Davies-Bouldin index (Figures S4 and S5 in Multimedia Appendix 1), 6 higher-level thematic groups were identified from 99 Reddit topics and 189 Weibo topics, respectively (Figure 3A and B).

The Reddit groups are as follows:

- Group 0: Navigating lupus diagnosis and daily struggles
- Group 1: Skepticism and sarcasm toward alternative health
- Group 2: High-risk pregnancy and infant medical journeys
- Group 3: Everyday life and COVID-19 precautions
- Group 4: Photosensitivity and visible symptoms in autoimmune disease
- Group 5: Struggles with autoimmune medications and treatment hesitancy (Multimedia Appendix 3)

The Weibo groups are as follows:

- Group 0: Immunity and yang qi regulation from a Chinese medicine perspective
- Group 1: Daily life and emotional states of lupus patients
- Group 2: Inspirational stories of fighting illness
- Group 3: Everyday herbal health advice and recommendations
- Group 4: Basic knowledge and diagnostic confusion in rheumatic diseases
- Group 5: Emotional support and family bonding during illness (Multimedia Appendix 3)

**Figure 4.**
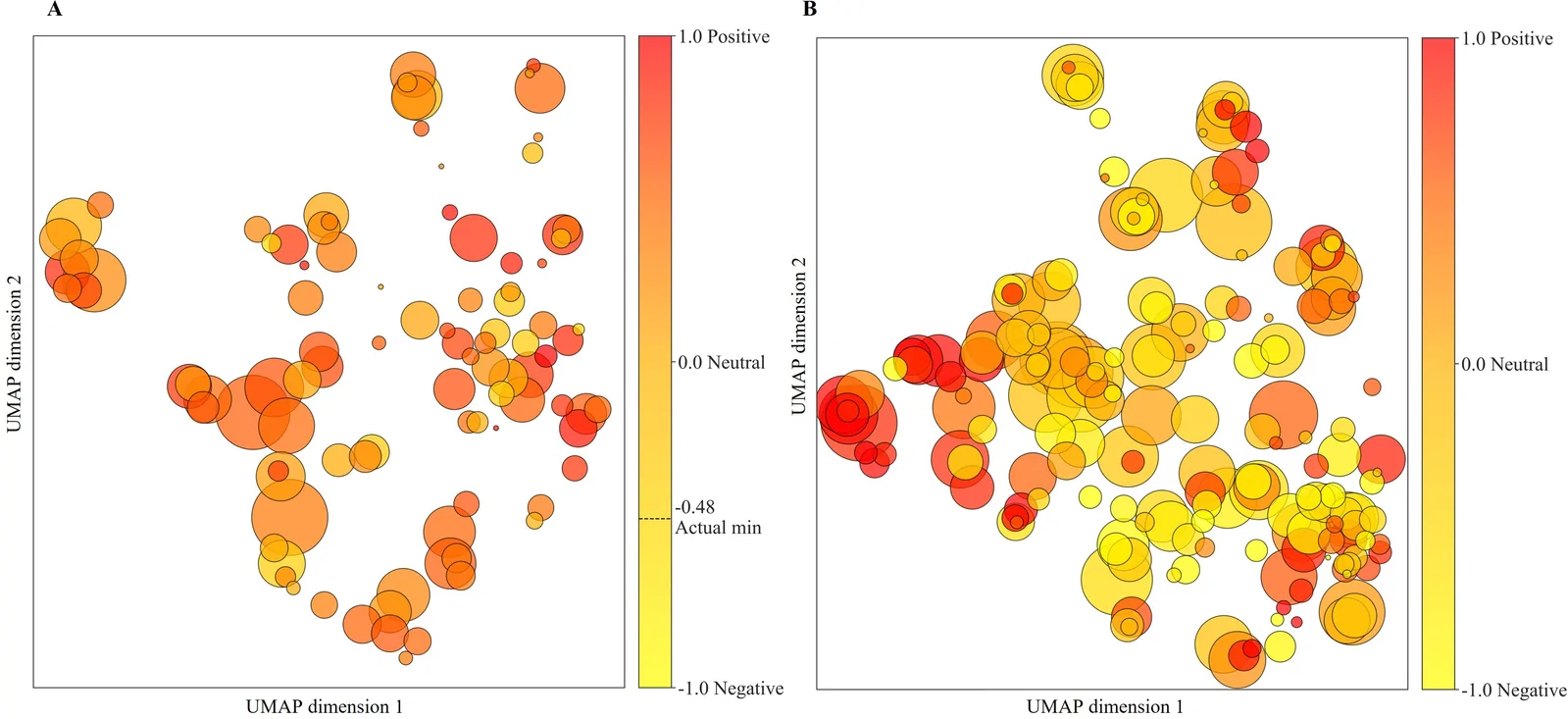
Topic-level sentiment analysis on Reddit (A) and Weibo (B). Each circle represents one topic, with its size proportional to the number of discussions; color indicates the mean sentiment score, where yellow reflects a negative orientation (closer to −1) and red reflects a positive orientation (closer to +1). UMAP: uniform manifold approximation and projection.

The temporal variation in discussion volumes across these thematic groups is presented in Figure 3C-F. As shown in Figure 4, the overall sentiment of 11,318 SLE-related discussions on Reddit was positive, with a mean sentiment score of 0.42 (SD 0.86; 95% CI 0.40-0.43). Of these, 7493 discussions were classified as positive, 1032 as neutral, and 2793 as negative. Examples of highly negative discussions included statements such as “Feeling like I was going to die anytime,” whereas highly positive examples included “Great advice on hydration and stress. Super important,” and “I think I will keep getting better to a point. Yay, great to hear!!” At the topic level, the lowest mean sentiment score was observed for topic 78 (mean −0.48, SD 0.89; 95% CI −0.81 to −0.15), which focused on pregnancy and postpartum-related discussions. Despite this, all thematic groups exhibited an overall positive sentiment (Table S4 in Multimedia Appendix 1).

On Weibo, the overall sentiment of 33,628 discussions was neutral, with a mean sentiment score of 0.01 (SD 0.91; 95% CI 0.00-0.02). Among these, 14,026 discussions were classified as positive, 5768 as neutral, and 13,834 as negative. Examples of highly negative discussions included “It’s so hard. I was just diagnosed two months ago, and I’m still a senior in high school. Please help??” and “I’m only 30 years old. If I don’t survive the New Year, could you all go to the beach for me?” In contrast, highly positive examples included “Take less medicine and keep your body super healthy. Come on, come on, come on!” and “It’s been nearly ten years since I stopped taking medication. Lupus is not as terrifying as people think.”

The lowest sentiment score was observed for topic 188 (mean −1.00, SD 0.00; 95% CI −1.00 to −1.00), which focused on anger and conflicts with older adults. Three thematic groups reflected predominantly negative emotions: immunity and yang qi regulation from a Chinese medicine perspective (group 0: mean −0.25, SD 0.94; 95% CI −0.27 to −0.23); inspirational stories of fighting illness (group 2: mean −0.29, SD 0.83; 95% CI −0.25 to −0.21); and emotional support and family bonding during illness (group 5: −0.06, SD 0.95; 95% CI −0.09 to −0.03). The remaining 3 groups demonstrated positive emotions (Table S4 in Multimedia Appendix 1).

## Discussion

### Principal Findings

This study systematically analyzed SLE-related content on Reddit and Weibo using AI-based methods, identifying 99 and 189 topics from 11,318 Reddit and 33,628 Weibo discussions, respectively, with each platform’s topic further clustered into 6 thematic groups. Both platforms converged on core clinical and psychosocial themes, including symptom management, medication side effects, and emotional support, yet diverged in emphasis. Reddit discussions centered on individualized diagnosis and treatment management, whereas Weibo discussions gave greater visibility to collective, socially oriented narratives around charity and integrative medicine such as TCM (eg, artemisinin). Because the platforms differ in user composition, moderation practices, linguistic contexts, and communication styles, these differences likely reflect a combination of sampling, platform-specific, and cultural factors rather than culture alone. Moreover, social media users are a self-selected subgroup whose concerns may not fully represent the broader SLE population.

Consistent with previous studies, users on TikTok and 13 other English-language social media platforms focus on daily symptom management [34,35]; YouTube videos emphasize information quality and health education [36]; and vertical communities, such as PatientsLikeMe, center discussions around symptoms and quality of life [37]. Together, this evidence underscores the platform-specific roles that different media play within the broader disease information ecosystem, suggesting that public health education and social media–based interventions should be tailored to the user base and communication style of each platform.

Sentiment analysis showed an overall positive tendency on Reddit (mean 0.42, SD 0.85; 95% CI 0.40-0.43) and a neutral tendency on Weibo (mean 0.01, SD 0.91; 95% CI 0.00-0.02; Figure 4). More clinically meaningful than these platform-level averages were the negative clusters within specific topics and thematic groups, notably pregnancy-related and postpartum-related discussions on Reddit (topic 78) and anger, intergenerational conflict, and Chinese medicine–oriented discussions on Weibo (topic 188 and group 0). People with SLE are known to experience high rates of depression, anxiety, and fatigue [38], which are closely associated with disease activity, medication side effects, and insufficient social support. TikTok research has likewise shown that pharmacological treatments are portrayed negatively [34], and 17.2% of people with SLE reported negative emotions during the COVID-19 pandemic that were linked to social media content [39]. These findings highlight emotionally vulnerable subgroups among people with SLE and the need for targeted psychological support around fertility, family relationships, and major life events, as well as ongoing monitoring of panic and misinformation on such platforms. Building on frameworks such as online communities and patient registries proposed by Blackie et al [40], integrating social media with AI-driven analysis offers valuable opportunities for people with SLE to better manage their conditions and to understand public perceptions and unmet needs [35,40].

Frequent discussions of TCM and herbal therapies in the Weibo corpus suggest that these approaches were salient among Chinese-speaking users with SLE. This interest may stem from concerns about long-term adverse effects of conventional therapies, heightened by the chronic, relapsing nature of SLE that necessitates lifelong treatment, together with culturally rooted health beliefs. Active compounds in TCM, such as artemisinin, have shown potential in modulating immune function and reducing disease activity [41,42], and TCM is often perceived as a gentler, complementary approach. A Chinese cross-sectional study of 351 people with SLE found that 85.5% regularly used at least 1 complementary therapy [43]. These preferences may also reflect structural factors in China’s health care system: approximately 85.3% of rheumatologists practice in tertiary hospitals concentrated in urban centers [44], creating access barriers in less-developed regions and increasing reliance on online health information [12-14]. By contrast, Reddit users within Western health care systems more frequently sought diagnostic clarification and engaged in treatment decision-making, reflecting the role of online communities in supporting informational needs and self-management in chronic conditions [45]. As patient motivations, treatment behaviors, and health-system influences were not directly assessed, these interpretations should be regarded as plausible contextual factors rather than definitive conclusions.

Moreover, cross-platform differences should not be attributed solely to cultural or health-system factors. Research suggests that differences in platform affordances, user communities, and health-system contexts shape not only what health topics are discussed but also how illness experiences are framed and shared [46]. Weibo’s algorithmic curation and hashtag-based aggregation increase the visibility of thematically related and culturally familiar content, shaping how users identify and prioritize health information. Reddit’s threaded format and community norms, by contrast, foster more deliberative, evidence-oriented exchanges that direct users toward procedural information. These structural features indicate that platform design does not merely reflect preexisting user preferences but actively shapes the information environments users encounter, the credibility cues they rely on, and ultimately the illness narratives they construct and share.

Discussions surrounding SLE on both Reddit and Weibo increased markedly between 2019 and 2021. This surge coincided with the COVID-19 pandemic. During this period, people with SLE frequently reported feelings of losing control over their health, accompanied by heightened anxiety and stress [47], while overall news consumption and social media use also rose significantly [39,48]. Notably, at the peak of the pandemic, the number of COVID-19–related communities on Weibo increased by nearly 40% compared with the prepandemic period [49,50]—a rise substantially greater than that observed on Reddit. This discrepancy may reflect differences in user engagement between the 2 platforms, as well as potential gaps in the Reddit data available for analysis. A second notable spike in SLE-related discussions on Weibo occurred from 2023 to 2024, which may be attributed to celebrity influence [51]: the death of a well-known actor from SLE may have drawn renewed public attention to the disease, possibly contributing to a wave of popular science communication and a new focal point in public discourse.

When interpreting the topic modeling results, we observed topics on both platforms containing treatment-related misinformation alongside concerns reported by people with SLE and culturally situated health beliefs. One recurring pattern was concerns reported by people with SLE about pharmacological treatment, where distressing experiences were generalized into cautionary claims (eg, “Taking HCQ for a week and just had a second acute attack of extreme pain and diarrhea and just decided it cannot go on, it just cannot, I am unable to live like this.” [Reddit topic 7]; “Taking hormones to treat lupus erythematosus causes femoral head necrosis!” [Weibo topic 74]). Alternative and complementary therapies such as the autoimmune protocol (AIP) diet and supplements, including Reishi mushrooms and brown algal astaxanthin, were occasionally framed as curative alternatives (eg, “I also take natural, ground Reishi mushroom capsules. They contain a heap of healing powers...and are very effective at healing lupus.” [Reddit topic 73]; “Lupus erythematosus...we must take brown algal astaxanthin.” [Weibo topic 175]). A few topics referenced scientifically unsupported or commercially promoted “cures” (eg, “I went to a ‘Hindu’ monk when I was 11/12, and he told me I could be cured if I drank my own urine every morning for a year.” [Reddit topic 80]; “The China International Academy of Medicine has invented a drug that can cure lupus erythematosus... [Skin No. 1]...” [Weibo topic 117]). Polarized disease perceptions and pessimistic narratives likely reflected emotional responses to chronic illness rather than misinformation in a strict sense (eg, “She had lupus erythematosus, which was more serious than cancer...” [Weibo topic 108]; “I knew that I had lost the right to live a normal life…” [Weibo topic 1]). Some posts also reflected distrust of physicians and a stronger inclination to trust peer experiences, potentially facilitating the circulation of unverified content (eg, "You can’t really speak to every doctor’s intentions either. Plenty of people have been told that they are making things up, for instance.” [Reddit topic 82]). As Barahona-Correa et al [36] observed, some individuals use social media platforms as their default search engines, and the easy access to unverified information makes these platforms a potent channel through which misconceptions may influence public perception and treatment decisions.

We did not quantify the prevalence of these content types or formally distinguish misinformation from legitimate patient experiences, so these cross-platform similarities should be interpreted as exploratory observations rather than empirically validated patterns. Nevertheless, their recurrence across both Reddit and Weibo suggests that treatment-related misconceptions and unverified remedies may pose shared challenges in online communities of people with SLE, warranting dedicated studies with systematic misinformation identification protocols grounded in clinical guidelines.

### Research Prospects and Significance

This study presents several notable strengths. First, unlike traditional public opinion assessments that often rely on Gallup-style self-reported surveys, our research incorporates internet platforms into the measurement framework, thereby extending the methodological boundaries of information systems [52]. Internet-based data can serve as an early warning signal for public health and contribute to the detection and correction of health-related misinformation [53,54]. Simultaneously, these data provide access to authentic concerns and lived experiences reported by people with SLE—insights that are difficult to capture through traditional channels—thus offering valuable evidence to improve medical communication and optimize clinical workflows [55]. Furthermore, emotional support and information sharing within online communities may enhance patient empowerment and satisfaction. Importantly, because these data are generated in natural, noninterventional contexts, the risk of observation bias is minimized.

Another strength of this study lies in the parallel comparison of discussions across 2 major social media platforms, Reddit and Weibo. In contrast to studies that focus on a single platform, our cross-platform and cross-cultural design not only reveals differences in information diffusion and disease perception but also highlights platform-specific discourse patterns. This approach broadens the comparative scope of our findings and provides valuable insights for addressing the current research gap in public health communication across cultural settings [56,57].

### Limitations

Several limitations should be acknowledged. First, because the abbreviation “SLE” was excluded from the Weibo search terms, Weibo discussions that used this abbreviation rather than the full Chinese disease terms may have been missed, which may have led to an underestimation of the Weibo corpus and a slight underrepresentation of users who prefer shorthand expressions. Second, as SLE disproportionately affects women—with reported female-to-male ratios ranging from 1.2:1 to 15:1 [58,59]—and women are generally more active on social media [60], the absence of demographic information such as gender limited our ability to adjust for sample structure or examine gender-related differences in discussion activity. Third, the unreviewed nature of social media content introduces substantial volumes of false or misleading information (eg, COVID-19 vaccine–related rumors), complicating data cleaning and potentially affecting the reliability of conclusions [61]. Fourth, social media users constitute a self-selected subgroup that likely differs systematically from the broader population of people with SLE: individuals with greater disease burden, stronger emotional needs, or higher health literacy may be more inclined to contribute to online discussions, while those with limited internet access, lower digital literacy, or from underrepresented groups remain largely absent. Therefore, the concerns and needs identified may not represent the full spectrum of experiences among people with SLE, and caution is warranted when generalizing the findings [62]. Fifth, a small number of highly active users contributed a disproportionately large share of content, while the voices of the majority of “silent” users remained underrepresented. This imbalance may have exaggerated the apparent engagement and influence of certain subgroups [63]. Finally, the cross-lingual design may have introduced additional bias: machine translation of Chinese content may have altered culturally specific or emotionally nuanced expressions, and although multilingual embeddings enabled joint analysis of Chinese and English texts, they may not have captured culturally embedded meanings with equal accuracy across languages [64]. Cultural differences in emotional expression may have influenced sentiment classification and thematic interpretation. Furthermore, the 2 platforms were sampled using different strategies: Reddit data were drawn from a single subreddit (r/lupus), whereas Weibo data were obtained through broader keyword-based retrieval. This difference may affect the representativeness of each corpus and the strength of cross-platform generalization.

### Future Directions

Future research should incorporate clinical indicators, patient-reported outcomes, and registry data to validate whether social media sentiment reflects disease burden and quality of life among people with SLE, and the integration of such data into AI predictive models could enable real-time monitoring of experiences among people with SLE, prediction of disease trajectories, and the development of personalized, data-driven support strategies.

### Conclusions

In summary, this study demonstrates the feasibility of using AI to perform topic modeling and sentiment analysis on large-scale, cross-cultural, and cross-platform social media data related to SLE. Beyond this, the distinct informational and emotional needs across platforms indicate that culturally tailored communication strategies are needed for people with SLE, while the recurrence of treatment-related misconceptions calls for clinicians and public health authorities to engage with online communities of people with SLE. More broadly, this cross-platform approach provides a transferable framework for real-time monitoring of patient perspectives beyond SLE.

## Supplementary material

10.2196/95175 Multimedia Appendix 1 Supplementary methods, references, figures, and tables supporting topic modeling, sentiment analysis, sensitivity analyses, and GPT-5 Thinking parameter settings.

10.2196/95175 Multimedia Appendix 2 Operational definitions of sentiment categories.

10.2196/95175 Multimedia Appendix 3 Overview of groups of topics with example text.

## Abbreviations

AIP: autoimmune protocol
ANA: antinuclear antibody
BERT: bidirectional encoder representations from transformers
c-TF-IDF: class-based term frequency–inverse document frequency
ERE: entity relation extraction
HDBSCAN: hierarchical density-based spatial clustering of applications with noise
HRQoL: health-related quality of life
LLM: large language model
NER: named entity recognition
NLP: natural language processing
OHCs: online health communities
S2ORC: Semantic Scholar Open Research Corpus
SLE: systemic lupus erythematosus
TCM: traditional Chinese medicine
UMAP: uniform manifold approximation and projection
