# Supplementary material for: AI-Driven Topic Modeling and Sentiment Analysis of Systemic Lupus Erythematosus Discussions on Social Media: Cross-Platform Study
Source: J Med Internet Res. 2026 Aug 31;28:e95175. doi: 10.2196/95175 (PMC13528885; doi:10.2196/95175)
Supplement: Supplementary file 1 [file jmir-v28-e95175-s001.docx]

**Supplemental Online Content**

**eMethods.**

**Topic model**

**eReferences**

**Figure S1.** Histogram of Number of Discussions per Author

**Figure S2.** BERTopic Parameter Search

**Figure S3.** Topic Evolution Over Time

**Figure S4.** Group Determination Sensitivity Analysis – Reddit

**Figure S5.** Group Determination Sensitivity Analysis – Weibo

**Table S1.** Grid search parameter ranges for BERTopic optimization

**Table S2.** Sentiment Analysis Model parameters

**Table S3.** Topic Overview

**Table S4.** Mean Sentiment Across Groups

**Table S5.** Parameter Settings of GPT-5 Thinking

This supplemental material has been provided by the authors to give readers additional information about their work.

**eMethods**

**Topic model**

The BERT model dynamically adjusts word vector representations according to their specific context within a sentence, thereby generating context-aware text embeddings. This capability effectively addresses lexical ambiguity and allows for the capture of richer semantic and syntactic information[1].

To handle the high complexity of SLE-related discussions on Reddit and Weibo, as well as the domain-specific characteristics of medical discourse, we adopted BERTopic, an advanced topic modeling approach built on BERT. BERTopic operates in a staged and modular fashion, encompassing four core components: semantic representation, dimensionality reduction, topic clustering, and topic representation[2].

To ensure that the model could accurately interpret social media discussions on SLE across different languages and cultural contexts, we selected Multilingual-E5-base at the semantic representation stage. This sentence-level pretrained language model, built on the Transformer architecture, constructs a unified, cross-domain semantic space in which clinical terms (e.g., “lupus nephritis”) and layperson expressions (e.g., “kidney problems and body swelling”) are mapped to nearby vector positions with high accuracy[3].

We then applied the Uniform Manifold Approximation and Projection (UMAP) algorithm for dimensionality reduction. Compared with linear methods such as PCA, UMAP projects the high-dimensional semantic space into a lower-dimensional space while preserving as much of the original semantic neighborhood structure as possible, thereby creating an optimal input environment for clustering algorithms[4].

Within this reduced semantic space, we employed Hierarchical Density-Based Spatial Clustering of Applications with Noise (HDBSCAN) to identify topics. By defining clusters based on point density, HDBSCAN can flexibly recognize semantic groups of varying shapes and sizes, while excluding outliers such as advertisements, spam, and off-topic discussions, ensuring that the analysis remains focused on meaningful content[5].

However, given its tendency to over-detect outliers in smaller datasets, we further incorporated spectral clustering to identify low-dimensional representations of similarity matrices between data points. To optimize model performance, we performed a grid search across four key parameters known to affect topic model quality **(Table S1)**. For each parameter combination, an independent BERTopic model was constructed and evaluated using the Silhouette Coefficient (SC), Calinski–Harabasz (CH) index, and Davies–Bouldin (DB) index to determine the optimal number of clusters[6].

After clustering, BERTopic applied class-based term frequency inverse document frequency (c-TF-IDF) to extract representative keywords for each topic, taking into account the distribution of documents within each cluster. Finally, a cosine distance matrix of c-TF-IDF representations was calculated, and Ward’s linkage hierarchical clustering was performed to generate a coarse-grained hierarchical structure of topics, which was visualized using a dendrogram.

Because BERTopic generates highly fine-grained topics, it is particularly important to identify broader thematic groups from these subdivided clusters. To achieve this, we first represented each topic numerically using its c-TF-IDF vector. Given the high dimensionality of this feature space, dimensionality reduction was performed using UMAP. In the reduced space, spectral clustering was applied to group the topics. Spectral clustering was chosen because of its ability to effectively identify non-convex clusters, making it well suited to capture the complex interrelationships among topics. Since spectral clustering requires the number of clusters to be specified in advance, we conducted a systematic sensitivity analysis to determine the optimal grouping. Specifically, the number of clusters was iteratively varied from 3 to 20, and for each setting we computed the Silhouette Coefficient and Davies–Bouldin Index. The Silhouette Coefficient assesses clustering quality by evaluating intra-cluster cohesion and inter-cluster separation, with values closer to 1 indicating better results. The Davies–Bouldin Index quantifies the ratio of intra-cluster dispersion to inter-cluster separation, with lower values (closer to 0) reflecting superior performance. Based on the combined performance of these two metrics, the optimal number of macro-level groups was determined.

After clustering, each group was characterized by extracting representative keywords using c-TF-IDF. To facilitate interpretation of the topic modeling results, higher-level thematic labels were generated using GPT-5 Thinking (OpenAI API, accessed August 30, 2025). Model parameter settings are reported in Table S5. For each platform, topic clusters identified through BERTopic were represented by their top-ranked keywords and a subset of representative documents, which together served as the basis for thematic abstraction.

eReferences

[1] D Devlin J, Chang M, Lee K, Toutanova K. BERT: Pre-training of deep bidirectional transformers for language understanding. Proceedings of the 2019 conference of the north american chapter of the association for computational linguistics: Human language technologies, Volume 1 (Long and Short Papers); Jun 6-7, 2018: 4171-4186; Minneapolis, Minnesota. Association for Computational Linguistics.
[2] Grootendorst M. BERTopic: Neural topic modeling with a class-based TF-IDF procedure. arXiv preprint. arXiv: 2203. 05794. 2022.
[3] Wang L, Yang N, Huang X, Yang L, Majumder R, Wei F. Multilingual E5 text embeddings: a technical report. arXiv preprint. arXiv: 2402. 05672. 2024.
[4] McInnes L, Healy J, Melville J. Umap: Uniform manifold approximation and projection for dimension reduction. arXiv preprint. arXiv: 1802. 03426, 2018.
[5] McInnes L, Healy J, Astels S. hdbscan: Hierarchical density based clustering. J Open Source Softw. 2017; 2(11): 205. doi: 10.21105/joss.00205
[6] Rousseeuw PJ. Silhouettes: a graphical aid to the interpretation and validation of cluster analysis. J Comput Appl Math. 1987 Nov; 20: 53-65. doi: 10.1016/0377-0427(87)90125-7

**A**

**B**

**Figure S1. Histogram of Number of Discussions per Author.** (A) Histogram of Number of Discussions per Author – Reddit; (B) Histogram of Number of Discussions per Author - Weibo

The frequency distribution of the number of discussions (posts and comments) maded by each author on Reddit and Weibo. Apply the kernel-density estimator to make the histogram smoother

**A**

**B**

**Figure S2. BERTopic Parameter Search.** (A) BERTopic Parameter Search - Reddit; (B) BERTopic Parameter Search - Weibo

Visualization results of the multi-parameter joint search in the BERTopic modeling task on Reddit (A) and Weibo (B). The X-axis represents the synergistic PCA dimensionality reduction outcomes derived from four parameters: min_cluster_size (HDBSCAN), n_neighbors (UMAP), n_components (UMAP), and nr_topics (BERTopic). The Y-axis denotes the integrated evaluation score combining the Silhouette Coefficient、Calinski-Harabasz indexand and Davies-Bouldin index, where higher values indicate greater precision and rationality in topic partitioning.

**A	B**

**Figure S3.** **Topic Evolution Over Time.** (A) Topic Evolution Over Time - Reddit; (B) opic Evolution Over Time - Weibo

The distribution of the frequency changes of all posts and comments within each topic on Reddit and Weibo over time.

**Figure S4.** **Clustering Performance - Reddit**

The optimal number of groupings for the 99 topics on Reddit was determined using the silhouette score (top) and the Davies-Bouldin score (bottom). Higher silhouette score and lower Davies-Bouldin score indicate better clustering performance, which was maximized at n = 6 groups (“number of clusters”).

**Figure S5. Clustering Performance - Weibo**

The optimal number of groupings for the 189 topics on Weibo was determined using the silhouette score (top) and the Davies-Bouldin score (bottom). Higher silhouette score and lower Davies-Bouldin score indicate better clustering performance, which was maximized at n = 6 groups (“number of clusters”).

**Table S1**. **Grid search parameter ranges for BERTopic optimization**

| **Parameter** | **Algorithmic Component** | **Description** | **Search range** | **Optimal (Reddit)** | **Optimal (Weibo)** |
| --- | --- | --- | --- | --- | --- |
| n_neighbors | UMAP | Controls the balance between local and global structure during dimensionality reduction. | **8, 12, 16, 20, 24** | **8** | **24** |
| n_components | UMAP | Number of dimensions in the target embedding space after dimensionality reduction. | **9, 12, 15, 18, 21** | **15** | **12** |
| min_cluster_  size | HDBSCAN | Minimum number of documents required to form a valid topic cluster. | **14, 20, 26, 32, 38** | **20** | **26** |
| nr_topics | BERTopic | Target number of topics output after merging similar clusters. | **100, 130, 160, 190, 220** | **100** | **190** |

**Table S2 Sentiment Analysis Model parameters**

| **Parameter** | **Value** |
| --- | --- |
| Batch size | 64 |
| Epoch | 10 |
| Learning rate | 1e-5 |
| Max length | 256 |

**Table S3. Topic Overview**

The table lists the number of discussions, key words and representative texts (the text closest to the average value of the numerical values of this cluster) for each topic on Reddit and Weibo. The selected keywords are the most common words for the topic based on the class-based term-frequency inverse-document frequency (c-TF-IDF), these words and the documents best describe the topic.

| **Reddit** | | | | |
| --- | --- | --- | --- | --- |
| **Topic** | **Discussions** | **Group** | **Keywords** | **Representative** |
| 0 | 2475 | 0 | 'ana', 'positive', 'symptoms', 'lupus', 'negative', 'pain', 'test', 'anti', 'back', 'also', 'blood', 'tests', 'rheumatologist', 'like', 'low', 'could', 'dsdna', 'get', 'rash', 'high' | Yep, I developed the burning tongue also. Anything with the slightest spice or hot (temperature) will trigger it for hours. Cold soothes it for me. Symptoms started in March for lupus and the burning tongue started 4 weeks ago-ish. |
| 1 | 1983 | 0 | 'thank', 'thanks', 'hope', 'much', 'sorry', 'good', 'glad', 'luck', 'yes', 'sharing', 'well', 'appreciate', 'hear', 'wow', 'll', 'love', 're', 'oh', 'great', 'lol' | I will, thank you. |
| 2 | 569 | 4 | 'biopsy', 'rash', 'malar', 'dermatologist', 'derm', 'biopsied', 'rashes', 'cellular', 'get', 'skin', 'lupus', 'see', 'level', 'whether', 'tell', 'scar', 'face', 'would', 'rosacea', 'related' | I mean, it very well could be a malar rash. However, there are several conditions that can cause a malar rash, not just SLE. A biopsy done by a dermatologist and sent to pathology is the only way to know 100% if a rash is a lupus rash. Probably not what you wanted to hear, but all we can really tell you is yes, you have redness that’s visible to other people. Thank you for the reply, it really was helpful. I will try to see a dermatologist, I didn't know they could actually biopsy the rash. |
| 3 | 356 | 0 | 'feel', 'good', 'thank', 're', 'like', 'know', 'hope', 'someone', 'think', 'much', 'hard', 'sorry', 'better', 'things', 'really', 'day', 'life', 'take', 'support', 'going' | Yeah, I have been diagnosed for 3 months and I won’t lie now I feel better I feel really guilty as I haven’t been working and my family are supporting me! I feel like I’m making it up even though I actually wanted to die not that long ago because I was in so much pain. I don’t really know how to make it better but you’re definitely not alone in how you feel lol Thank you for sharing! For starters, I’m so glad you’re feeling better. Second, I totally know what you mean. I also find that it can be really hard to think about your pain retrospectively. |
| 4 | 272 | 0 | 'lupus', 'hope', 'diagnosis', 'people', 'get', 'life', 'well', 'going', 'knew', 'know', 'years', 'think', 'want', 'doctor', 'thank', 'really', 'feel', 'diagnosed', 'like', 'told' | this is different and not what i was talking about I realize that, and I think I know which post(s) you are referencing. It’s important to keep and open mind; some people just want an answer and are willing to take on the wolf. If it’s a true diagnosis, they might just change their minds however. I understand wanting an answer. That’s not my point. |
| 5 | 260 | 4 | 'sun', 'heat', 'sunscreen', 'uv', 'exposure', 'summer', 'temp', 'temperature', 'hot', 'always', 'body', 'outside', 'like', 'get', 'car', 'fever', 'makes', 'time', 'beach', 'feel' | I thought it was normal to need a 3 hours nap after spending some time in the sun. I thought 2 days rest was normal after a day at the beach. This. I've always loved the beach and sun, so I just thought getting tired/feeling sick a lot during the summer after ANY bit of sun exposure was just part of all the new things that came with being 30. |
| 6 | 207 | 5 | 'hcq', 'effects', 'taking', 'stomach', 'side', 'mg', 'take', 'hydroxychloroquine', 'months', 'ldn', 'dose', 'issues', 'start', 'years', 'meds', 've', 'medication', 'tolerate', 'started', 'starting' | Also check what you taking (fluid, food, other meds, etc) along with HQC. I learned the hard way that HCQ doesn’t play well with stimulants (especially adhd meds) or anti-histamines. Neither which by the way are listed as reacting to HCQ on drugs. com. |
| 7 | 188 | 5 | 'benlysta', 'tried', 'cellcept', 'infusions', 'methotrexate', 'effects', 'side', 'infusion', 'plaquenil', 'take', 'failed', 'medication', 'months', 'fatigue', 'steroids', 'good', 'imuran', 'first', 'years', 'injection' | Can I ask if it was the first medication you took? Or was it after other trials of medications? You can’t usually take Benlysta first. Most insurance companies require step therapy. I take plaquenil daily. |
| 8 | 176 | 5 | 'plaquenil', 'medications', 'disease', 'prednisone', 'medication', 'uctd', 'biologics', 'people', 'take', 'risks', 'effects', 'organ', 'study', 'side', 'lupus', 'taking', 'prescribed', 'rheumatologist', 'meds', 'progression' | Oh I need around 2 hours of rest after showering. So it’s shower every other day for me. Water wipes are my best friends. My diagnosis is uctd but my rheum notes all say early lupus. I feel like I’m stuck in some kind of hell. I take my plaquenil that doesn’t do much for my symptoms. |
| 9 | 160 | 3 | 'covid', 'vaccine', 'booster', 'vaccinated', 'flu', 'vaccines', 'got', 'shot', 'arm', 'long', 'shots', 'sick', 'pfizer', 'sore', 'boosters', 'felt', 'days', 'gotten', 'first', 'months' | I think it’s possible that it could be long-term covid, but I don’t remember ever getting Covid before these symptoms started. I did however get Covid this last December. |
| 10 | 159 | 3 | 'company', 'job', 'work', 'jobs', 'home', 'flexible', 'working', 'office', 'days', 'health', 'hours', 'remote', 'stress', 'time', 'full', 'hired', 'pharmacy', 'courthouse', 'career', 'schedule' | I think jobs either that are remote/work from home or jobs that are flexible and as long as you accomplish the work, are good options. Some jobs I've considered before & might consider in the future if my current situation changes: courthouse translation (have to better my fluency but def not off the table)courthouse transcriber (where they talk and you keyboard it in)big corporate jobs as data entry/entry level jobs - think like your utility company, your internet company, your Healthcare company, etcif you can consider accounting, it can be a high stress job where you can’t control anything and rely on your company/customers to statt shaping up but you end up getting paid bank as you raise your statushealth inspector (on your own time but might make you drive to a larger city and drive around // my health inspector doesn't come in when it rains and seems to take a lot of pto) nonprofit work (may need to volunteer within a field of npo to gather more confidence & experience)if you can go back to school, biology labs seem pretty good as well. Depends if you can handle cold tho. |
| 11 | 150 | 0 | 'sound', 'lupus', 'sounds', 'like', 'none', 'neurology', 'gp', 'nebulous', 'symptoms', 'idea', 'think', 'heard', 'ankylosing', 'doesn', 'spondylitis', 'imuran', 'evaluate', 'could', 'would', 'see' | None of these sound like lupus specific symptoms. |
| 12 | 134 | 0 | 'rheumatologist', 'appointment', 'new', 'wait', 'rheumatologists', 'get', 'thank', 'rheum', 'reviews', 'first', 'one', 'doctor', 'months', 'ask', 'another', 'appointments', 'see', 'good', 'long', 'next' | If you live in a major city, you should be able to get an appointment sooner. Be honest with your doctor about not being comfortable with the time between appointments. You can even ask if he/she can refer you to another physician or if they have a PA you can see sooner. I do live in area with a lot of rheumatologists but there is only a few rheumatologists that take medicaid here so I'm assuming the good ones here are very booked cause of that. I'm thinking about asking my rheumatologist about the wait times on the next appointment but I dont want to be pushy especially alot of people had said this wait time is normalI keep ur advice in mind, thanks you for your suggestions :). |
| 13 | 129 | 3 | 'shower', 'heating', 'amazon', 'pad', 'chair', 'heated', 'tub', 'bench', 'wash', 'cook', 'blanket', 'packs', 'use', 'ice', 'hair', 'cooking', 'cleaning', 'bought', 'nice', 'teak' | You can get an affordable shower chair on Amazon. And you can get one that isn’t ugly. They have teak bench things that look really nice. Thankfully cuz most of the chairs were so ugly and it made me feel worse seeing only very old people in the photos lol , so it's funny you pointed that out. My mom has been searching on Amazon so I hope we find something decent but if not the alternatives are an option. |
| 14 | 127 | 0 | 'pain', 'fibro', 'fibromyalgia', 'causes', 'joints', 'yes', 'lupus', 'joint', 'hurt', 'doctors', 'idiot', 'symptom', 'painful', 'feels', 'touch', 'inflammation', 'aches', 'neuropathy', 'ability', 'absolutely' | also have trace protein in urine and abnormal EKG with irregularly irregular rhythm (sinus arrhythmia) Yep to all of this. It is very common for lupus patients to have fibro, but that pain from fibro isn't the same as lupus pain. I have been diagnosed w both and also experience the sjogrens and irritable bowel. My rheumatologist told me that the overall achiness in my joints and malaise is the lupus. The pain I experience from huge canker sores and headaches from stress or being in the sun too much, lupus. |
| 15 | 115 | 1 | 'birth', 'iud', 'control', 'mirena', 'hormonal', 'hysterectomy', 'periods', 'mfm', 'preconception', 'tits', 'counseling', 'panel', 'period', 'pill', 'aps', 'risk', 'apl', 'good', 've', 'miscarriages' | I’m in a similar boat but I’ve only been on mine for about 4 years before being diagnosed. Once I was diagnosed they wouldn’t let me take my birth control anymore. They said my only option is a non-hormonal iud which I am not getting an IUD. I told them I don’t care about blood clots or whatever just give me the same pill I’ve been taking and they still said no they won’t prescribe it. |
| 16 | 111 | 3 | 'mask', 'get', 'even', 'school', 'covid', 'like', 'trying', 'still', 'sick', 'time', 'home', 'bad', 'people', 'life', 've', 'going', 'really', 'one', 'feel', 'kids' | Growing up I’ve always been sick, I had really bad nose bleeds from too much heat/sun or when it’s extremely cold. Fevers there and there, one time one of my nails started looking bad, it looked like chicken breast that had been shredded, I could literally please off the strands one by one. My nail is fine now, some antibiotics helped me. |
| 17 | 110 | 1 | 'elevated', 'sympathy', 'labs', 'inconclusive', 'ratio', 'september', 'run', 'pttla', 'wbc', 'avise', 'invalidating', 'expectations', 'mm', 'testing', 'antiphospholipid', 'facts', 'mean', 'crp', 'empathy', 'tests' | Except the repeat testing for the aPL, it sounds like his did run most of the appropriate labs. Do you know what labs were left out? He didn’t run a Lupus Anticoagulant panel after my labs from a year ago were elevated and “inconclusive,” which could have told him more about my elevated cardiolipin IgM. He didn’t run a basic CBC to see if my white count was elevated due to the high inflammation based on my ESR and CRP. |
| 18 | 107 | 2 | 'flaired', 'users', 'comment', 'cld', 'dle', 'moderators', 'mctd', 'performed', 'bot', 'action', 'automatically', 'subreddit', 'contact', 'flair', 'posts', 'removed', 'concerns', 'uctd', 'diagnosed', 'please' | u/Business_Ear_4207, your comment was removed - only users flaired as SLE, UCTD/MCTD or CLD/DLE Diagnosed can comment on Diagnosed Users Only flaired posts. How to flair yourself here. I am a bot, and this action was performed automatically. Please contact the moderators of this subreddit if you have any questions or concerns. |
| 19 | 103 | 4 | 'moisturizer', 'skin', 'roche', 'makeup', 'use', 'cream', 'posay', 'face', 'sunscreen', 'products', 'skincare', 'serum', 'la', 'cetaphil', 'brands', 'niacinamide', 'powder', 'love', 'cosrx', 'cerave' | Mine personally doesn’t ever go fully away. I used to think it was rosacea but it never goes away. It’s not fantastic for my self esteem honestly, but I’ve got used to it now. P. |
| 20 | 103 | 3 | 'mask', 'masks', 'wear', 'wearing', 'covid', 'planes', 'plane', 'airport', 'overkill', 'travel', 'trip', 'people', 'masking', 'traveling', 'airports', 'public', 'crowded', 'sick', 'flights', 'worn' | I would never get on a plane without a mask again. Pre COVID I would get a cold every time I travelled as I'm also on azathioprine. I haven't travelled a lot since Covid started but I've always worn a mask and haven't got an infection yet. For me it's a minimal inconvenience saving me from potential life changing illness. Even if it's saving me a few days of the sniffles - totally worth it. |
| 21 | 102 | 0 | 'pcp', 'appointment', 'referral', 'rheumatologist', 'ask', 'rheum', 'hopefully', 'definitely', 'help', 'get', 'call', 'see', 'told', 'directions', 'wants', 'diagnosis', 'labs', 'answers', 're', 'boss' | Have you spoken with your rheum yet? No one here can help with what it could or could not be, but your rheum should be able to look at your whole picture and help. I follow up on September 4th but mainly just to go over the labs in person and I already know the results. rheumatologist has only said she’s leaning away from lupus but she doesn’t know and wants me to see a PCP. You should definitely ask her what she wants a PCP to investigate if that's the case. |
| 22 | 89 | 5 | 'mg', 'hydroxychloroquine', 'daily', 'folic', 'vitamin', 'acid', 'mycophenolate', 'prednisone', 'cellcept', 'benlysta', 'methotrexate', 'mofetil', 'celebrex', 'iron', 'bid', 'vit', 'hcq', 'infusions', 'azathioprine', 'twice' | Currently Mycophenolate 750 mg twice daily(currently)and hydroxychloroquine 200 mg daily and a couple BP medications. Have previously taken prednisone (as high as 60mg) and methotrexate (can’t remember the dose as it was so long ago). Thanks for your reply! So four total in your journey! |
| 23 | 85 | 4 | 'hoodie', 'sun', 'vampire', 'upf', 'spf', 'sunlight', 'shirts', 'carry', 'sunscreen', 'fabric', 'zinc', 'smears', 'backpacks', 'hats', 'oxide', 'sunblock', 'gloves', 'sleeves', 'uv', 'handbag' | I guess it depends on how sensitive you are to sunlight. They make UPF shirts that are a loose fit though. They’re like athletic shirts. I would think a hoodie would be sufficient, but I also don’t know how thick the fabric is, how tightly knit the fabric is, or how sensitive you are to sunlight. If you’ve used a hoodie before with good success, I would think using the same hoodie would still work for you- assuming your sensitivity to sunlight hasn’t changed since then. |
| 24 | 83 | 1 | 'aps', 'antiphospholipid', 'antibodies', 'anticoagulant', 'event', 'named', 'positive', 'clotting', 'acl', 'syndrome', 'dvvrt', 'clot', 'test', 'carrier', 'lupus', 'anticardiolipin', 'antibody', 'poorly', 'syphilis', 'confused' | So what’s the opposite of APS? Based on your comment, not having Beta 2 glycoprotein antibodies. I understand that. In Quest, it showed up as grey which I’m assuming means I am deficient. I know a positive antibody is associated with SLE—was wondering if a deficiency was linked to anything. |
| 25 | 83 | 2 | 'uctd', 'people', 'diagnosis', 'mctd', 'want', 'support', 'label', 'lupus', 'one', 'neat', 're', 'post', 'symptoms', 'think', 'us', 'treated', 'armchair', 'isn', 'box', 'disease' | How can I get ANY of my doctors to order tests to figure out what I have. They want to stop looking bc “it’s autoimmune so as long as you’re taking some immunosuppressant it shouldn’t matter”I keep getting passed around from specialist to specialist too, and I’m having a hard time advocating for myself. I’ve got a systemic autoimmune disorder, but my drs can’t figure out which one. Lupus seems to fit my symptoms& bloodwork the most. I get what appears to be a mild malar rash, but my rheumatologist won’t even look at pictures of it. |
| 26 | 80 | 1 | 'boost', 'tablespoon', 'diet', 'leafy', 'canned', 'blend', 'tried', 'citrus', 'awards', 'eat', 'fats', 'antioxidant', 'gluten', 'drink', 'powers', 'bingo', 'eating', 'toxins', 'intake', 'mediterranean' | No actually. The key to all of this is having no thoughts at all. You want to ensure you have not a single thought circulating in that brain. Straight static. Getting a weird, almost spider web looking rash when you’re out in the sun. |
| 27 | 77 | 1 | 'hair', 'shampoo', 'nizoral', 'conditioner', 'saved', 'omega', 'doctor', 'biotin', 'strands', 'rosemary', 'lab', 'results', 'loss', 'routine', 'collagen', 'stronger', 'falling', 'regrowing', 'person', 'wording' | Hi! Could you share what the doctor told you in regards to this? It’s still a little bit up in the air. I had a lupus panel done and it came back negative but my inflammatory markers were still higher than usual. Thankfully most of my symptoms have resolved since then. Thank you for your update. I got the same result but i have no symptoms and if anything i feel with low energy but thats about it. I got the lab work done because if seeing a dermatologist for hair thinning problems and she ordered the lab work just to check on my levels and everything is good except that which came all red and at the very top of the results and it aso said Ana Tier 1:80 H. |
| 28 | 76 | 2 | 'thread', 'post', 'closed', 'current', 'comments', 'please', 'week', 'see', 'comment', 'page', 'payed', 'starter', 'alphabetical', 'question', 'country', 'reflaired', 'new', 'weekly', 'wiki', 'questions' | Comments are closed for this post! Please see the current week's thread if you have questions. |
| 29 | 73 | 0 | 'sympathy', 'expectations', 'people', 'empathy', 'treatment', 'healthcare', 'added', 'life', 'invalidating', 'things', 'positively', 'lupus', 'didn', 'paltrow', 'gwyneth', 'mean', 'even', 'facts', 'bogging', 'relationship' | And try to understand our point of view and that of your doc’s. I listed facts. That’s not invalidating. I didn’t say you were invalidating. |
| 30 | 72 | 2 | 'questions', 'removed', 'submit', 'approve', 'editing', 'shorter', 'feedback', 'limited', 'words', 'bot', 'moderators', 'note', 'performed', 'action', 'automatically', 'subreddit', 'contact', 'question', 'concerns', 'won' | u/Healthy-Change6928 Your question was removed because diagnosis questions are limited to 400 words. Shorter questions get more feedback. Note: Editing your removed post won't approve it, you need to re-submit it. I am a bot, and this action was performed automatically. Please contact the moderators of this subreddit if you have any questions or concerns. |
| 31 | 70 | 1 | 'goldners', 'unitdo', 'thisishowitalwaysis', 'trinity', 'tens', 'upvote', 'misspell', 'uhh', 'brooke', 'sidebar', 'disingenuous', 'disengenius', 'golden', 'viridian', 'axis', 'juice', 'holy', 'protocol', 'context', 'guys' | what do you guys think about Dr. brooke goldners protocol?. |
| 32 | 68 | 5 | 'cbd', 'thc', 'gabapentin', 'mg', 'helps', 'cream', 'bloodstream', 'pain', 'cannabis', 'take', 'canada', 'recommend', 'tennessee', 'find', 'effective', 'government', 'legal', 'lot', 'balance', 'nerve' | Thank you for the info. I will have her try the CBD without thc first to see if that helps. I would most definitely pm you if it doesn’t work. |
| 33 | 68 | 4 | 'butterfly', 'rash', 'sun', 'gingers', 'shingles', 'face', 'ginger', 'ha', 'didn', 'like', 'blanchable', 'urticarial', 'argued', 'dollar', 'part', 'day', 'uneasy', 'red', 'redder', 'felt' | Thanks buddSo what does this doctor think happens when a finite space, like a joint capsule, becomes inflamed? Lupus is a known painful condition. It’s also like the OG autoimmune disease. It’s definitely covered in medical school. The fact that this doctor is a rheumatologist and is saying lupus isn’t painful is inexcusable. I would ask him to explain why he thinks your pain isn’t lupus related. |
| 34 | 66 | 1 | 'kidney', 'urine', 'urinalysis', 'protein', 'kidneys', 'blood', 'bacteria', 'rbc', 'creatinine', 'results', 'infection', 'would', 'nephritis', 'high', 'stone', 'foamy', 'biopsy', 'fine', 'clotted', 'nephrologist' | I had one a few moths ago and nothing there, so that's why I'm worried. I'm gonna ask my new rheumatologist to double checkI’m not sure why you would be worried then? It is rare for kidneys to just shut down so quickly. Also if you were bad off, you would be able to see the blood and foam in your urine. |
| 35 | 65 | 1 | 'diet', 'provide', 'studies', 'eating', 'cure', 'patients', 'thousands', 'carnivore', 'science', 'foods', 'scientifically', 'safer', 'people', 'one', 'sources', 'triglycerides', 'research', 'working', 'make', 'cholesterol' | All I know is Certain foods have been proven to promote inflammation in the body. Eliminate the inflammation causing foods, eliminate (or reduce) the inflammation in your body. I can say even getting rid of most of the culprits in my diet has helped. It’s tough to stick to though. |
| 36 | 65 | 0 | 'people', 'lupus', 'sub', 'someone', 'person', 'think', 'age', 're', 'like', 'know', 'diagnosed', 'tired', 'hard', 'get', 'gets', 'lot', 'reality', 'also', 'understand', 'say' | Ok this is my bad for not being more clear but basically every single gp i’ve had is somewhat dismissive (live in a pretty small city i’m not exactly spoiled for choice) and every time I try to do this they tell me to stop and that they can only focus on one problem per appointment and when I say i try to think things are connected they are literally baffled as to why I think my joint pain and skin lesions would impact each other. Is there a way that you think would be better than saying I think I have lupus but also is not going to get me told to many problems at once (i feel like they aren’t understanding I don’t want them to fix these things I just need some a referral or new labs)? Also I’m well aware it may not be lupus i just said possible lupus. Not sure why this seems to be the go to response to everyone who asks for help but that’s fine. I have a family history and related anticoagulant problems so i’m not just randomly grasping onto lupus. |
| 37 | 64 | 1 | 'raynauds', 'raynaud', 'purple', 'warm', 'blue', 'underneath', 'nipples', 'breasts', 'discoloration', 'toes', 'breast', 'red', 'never', 'feet', 'eventuality', 'cold', 'exceptionally', 'hands', 'butterfly', 'bad' | I look forward every week to an injection that causes me extreme anxiety and pain, because without it my legs don’t work and I can barely get out of bed. In the full sun, don’t forget! I have never had a butterfly rash, for me it was raynauds. I had an exceptionally bad attack after exposure to snow. |
| 38 | 63 | 5 | 'mg', 'prednisone', 'taper', 'pred', 'taking', 'take', 'dose', 'better', 'effects', 'day', 'side', 'feel', 'insomnia', 'moon', 'face', 'really', 'sleep', 'gua', 'melatonin', 'hope' | Even on 5mg that can happen? Guessing I've been on it too long. Since November of 2020. Every time I try to tapper, I start to flare and get joint pain. |
| 39 | 57 | 4 | 'rash', 'malar', 'biopsy', 'skin', 'rosacea', 'cause', 'redness', 'rashes', 'several', 'visual', 'lupus', 'derm', 'dermatologist', 'sun', 'looks', 'viruses', 'cutaneous', 'possible', 'derms', 'facial' | Dude, you’ve repeatedly posted this pic. A) we have no way to know by just a picture. Malar is not a diagnosis, it’s a descriptor. Several disease processes cause a malar rash. B) the only way to know for sure is a biopsy. |
| 40 | 56 | 0 | 'rheum', 'rheumatologist', 'referral', 'dx', 'ask', 'pcp', 'send', 'new', 'gp', 'stock', 'gi', 'recommendation', 'ed', 'neurodegenerative', 'one', 'rheumatology', 'saw', 'dr', 'specialists', 'see' | You could ask for a GI Dr and for a rheumatologist referral. |
| 41 | 55 | 0 | 'white', 'moderate', 'mild', 'german', 'hispanic', 'caucasian', 'severe', 'female', 'irish', 'family', 'activity', 'organ', 'involvement', 'asian', 'black', 'english', 'mostly', 'american', 'european', 'dad' | White female, 44, moderate with kidney involvement, RA, psoriatic arthritis, thyroid disease. First in family diagnosed at 8yrs old. |
| 42 | 54 | 2 | 'undiagnosed', 'people', 'weekly', 'questions', 'thread', 'sub', 'threads', 'post', 'traffic', 'posts', 'cop', 'flair', 'user', 'diagnosis', 'advice', 'headache', 'lupus', 'read', 'someone', 'answer' | I try to only offer that kind of advice for people who say they are undiagnosed. And though I’m still undiagnosed, I know how regular otc medications don’t even touch the pain. But people with chronic pain who are undiagnosed (like myself) are willing to try anything-Tylenol, Motrin, deep breathing, diet change, whatever. I think some who have finally been diagnosed might forget what it was like before they got a diagnosis, all the uncertainty, doubt by doctors, etc. |
| 43 | 49 | 2 | 'flaired', 'users', 'comment', 'cld', 'dle', 'moderators', 'performed', 'bot', 'mctd', 'action', 'automatically', 'subreddit', 'contact', 'diagnosed', 'posts', 'removed', 'concerns', 'uctd', 'flair', 'please' | u/Fit_Dragonfruit3602, your comment was removed - only users flaired as SLE, UCTD/MCTD or CLD/DLE Diagnosed can comment on Diagnosed Users Only flaired posts. How to flair yourself here. I am a bot, and this action was performed automatically. Please contact the moderators of this subreddit if you have any questions or concerns. |
| 44 | 49 | 0 | 'family', 'autoimmune', 'aunt', 'relatives', 'conditions', 'lupie', 'mom', 'lupus', 'diagnosed', 'sides', 'history', 'neighbor', 'vitiligo', 'great', 'mother', 'one', 'co', 'lucky', 'far', 'person' | Me. No family history of lupus or any other diagnosed autoimmune. |
| 45 | 47 | 0 | 'family', 'autoimmune', 'mom', 'sister', 'history', 'cousin', 'hashimoto', 'diseases', 'aunt', 'members', 'diagnosed', 'dad', 'side', 'disease', 'ra', 'diabetes', 'grandmother', 'cousins', 'mother', 'lupus' | I’m the only one in my family with lupus. My mom had Hashimoto’s but no other autoimmune issues. I had one aunt with RA. My rheumatologist is always saying that usually someone else in family has it but idk. |
| 46 | 46 | 0 | 'appointment', 'bloodwork', 'call', 'results', 'telehealth', 'months', 'wait', 'weeks', 'suggested', 'confused', 'openings', 'said', 'trend', 'told', 'practices', 'cant', 'first', 'methods', 'sure', 'sooner' | My initial rheum appointment was scheduled 8 months out. D: I called them and asked them to check for something sooner, and managed to get a telehealth appointment for "only" 4 months later. Might be worth calling and seeing if they have any telehealth openings or cancellations. The issue with the telehealth is the doctor told me to do the bloodwork 2 weeks before my appointment so I'd have to break this rule to even do this optionA few people are suggesting I just do that though and call in for results so I'm a little confused haha I apologize if I'm missing something obvious - can't you go get the bloodwork done and then go over the results at the telehealth appointment? My doctor told me to do the bloodwork 2 weeks before my appointment in may. I'm not sure why I cant do it earlier but that is what he said. |
| 47 | 45 | 1 | 'staph', 'infection', 'jewelry', 'piercings', 'lung', 'infected', 'seriously', 'hospital', 'timeso', 'fiasco', 'mrsa', 'pimple', 'antibiotics', 'nasty', 'one', 'giant', 'went', 'became', 'expecting', 'greatly' | Wow thank you so so much. Let me know how it goes! Im really not sure if you can help, but if you can it'd be greatly appreciated. In June I was in the hospital for a mrsa staph infection in my lung. Can this be related? Also the other one I had just like this went away on its own, I thought it was just a nasty pimple. |
| 48 | 43 | 1 | 'crystals', 'prism', 'disperses', 'rectum', 'begins', 'areadon', 'perianal', 'shove', 'key', 'forget', 'healing', 'sunlight', 'lobotomy', 'gotta', 'tums', 'gi', 'become', 'fog', 'major', 'uv' | OP didn't mention yoga and sunning their taint - I call bullshit! OBJECTION. HEARSAY. DIRECT YOURSELF TO #3. |
| 49 | 43 | 1 | 'bladder', 'epilepsy', 'rare', 'neurogenic', 'paralyzed', 'started', 'old', 'nerves', 'problem', 'population', 'dad', 'seizures', 'genetic', 'family', 'took', 'age', 'control', 'compensated', 'airbnb', 'owners' | My diagnosis took two months… but I was also super super sick. like couldn’t get out of bed. Couldn’t move for three months. My diagnosis took over a decade. I guess you could say that it took 31 1/2 years, as I had my first symptoms at 18 months old, and was diagnosed when I was 33 years old. |
| 50 | 41 | 5 | 'plaquenil', 'stomach', 'take', 'months', 'bed', 'brand', 'taking', 'diarrhea', 'vary', 'name', 'plaquinil', 'bathroom', 'imodium', 'stopped', 'hours', 'first', 'took', 'time', 'wake', 'tolerated' | Just had 21 vials over the course of 3 days. Plaquenil experience may vary. I take it before bed now since I've been on it almost 2 years, but I could not take it at night the first 8-9 months because I had severe stomach issues and diarrhea. So it pretty much there was no good time to take it but I'd take at least 8 hours before bed so I didn't have to wake up and run to the bathroom lol. Just sent a request to join the group! Thank you! |
| 51 | 41 | 1 | 'lyme', 'illness', 'spirochete', 'pathogen', 'believe', 'doxycycline', 'cdc', 'returned', 'protocol', 'tick', 'infectious', 'uncommon', 'resolved', 'acute', 'baseline', 'original', 'believes', 'negative', 'wrong', 'happened' | Yes I was. I was tested twice and was negative both times. I believe that the original illness was some kind of spirochete that could have been Lyme or something else. I did not believe the tests. |
| 52 | 40 | 5 | 'vision', 'exam', 'eye', 'retina', 'plaquenil', 'years', 'exams', 'hydroxochloroquine', 'damage', 'yearly', 'regular', 'eyes', 'glasses', 'blind', 'peripheral', 'ophthalmologist', 'notice', 'eyesight', 'check', 'bright' | Thanks. And I didn't mean to offend. I simply meant that none of your reasons will seem significant in the long run if you end up with vision loss. You'll struggle to justify that choice. After about two years on the meds. I started to lose my peripheral vision and started struggling to see things at a distance. |
| 53 | 39 | 4 | 'makeup', 'blush', 'cheeks', 'rosacea', 'looks', 'pink', 'redness', 'face', 'compliment', 'mine', 'lol', 'skin', 'socials', 'red', 'wear', 'cute', 'look', 'glasses', 'rash', 'always' | It’s def a compliment it looks very cute and subtle. But I understand not liking having a warm rash on your face all the time. I thought my malar rash was rosacea for a long time until I got diagnosed. |
| 54 | 38 | 1 | 'vitamin', 'biotin', 'take', 'hair', 'liquid', 'level', 'iron', 'vit', 'vitamins', 'supplements', 'regular', 'taking', 'flair', 'supplementing', 'drops', 'loss', 'foggy', 'supplement', 'resolved', 'levels' | Is there a product you recommend? Recently, I’ve been trying Mary Ruth’s B complex organic liquid drops, I got them on Amazon. They’re gluten, corn, soy, and nut free, vegan, and taste pretty good for being a vitamin supplement, they also make a regular multi vitamin formula I haven’t tried yet. I’ve also been using Purely Holistic Liquid Biotin drops with D3 to help my hair grow back after a bad flare caused major shedding. |
| 55 | 38 | 0 | 'fibro', 'fibromyalgia', 'pain', 'irritable', 'feels', 'lupus', 'touch', 'body', 'neuropathy', 'joints', 'sjogrens', 'aches', 'achiness', 'patients', 'experience', 'distinguish', 'syndrome', 'arthritis', 'sometimes', 'hurt' | I see some serious stand up comedy potential for you. The work IS at night. You must make plans 4-6 weeks in advance, but be prepared to get canceled the day of or week beforehand ♀I got a knot in my back. |
| 56 | 37 | 1 | 'mouth', 'dry', 'sjogrens', 'dryness', 'dental', 'dentist', 'biotene', 'eyes', 'teeth', 'salivary', 'sores', 'gland', 'toothpaste', 'glands', 'fluoride', 'meibomian', 'tooth', 'sjogren', 'lip', 'may' | Lupus also causes dry mouth and eyes. You could have both or it could be just from the lupus. A lip biopsy would confirm the sjogrens. Otherwise, the blood markers and all symptoms scream lupus. Fyi, I'm on the same dental instructions for my tooth issues from my dry mouth. |
| 57 | 37 | 5 | 'prednisone', 'colonoscopy', 'vasculitis', 'dapsone', 'huvs', 'gastroenterologist', 'time', 'foreign', 'mg', 'plaquenil', 'cancer', 'tried', 'would', 'axial', 'sjogren', 'accutane', 'terrible', 'help', 'increased', 'take' | So perhaps it isn't lupus. If you already have Sjogren's and it's active, the steroids should be helping symptoms. They don't work just sometimes on autoimmune disease, they work all the time. (on RA too). What symptoms are you having? That’s what I was wondering too. Why didn’t the prednisone even help the RA? I had maybe a few hours where my joints felt better but that was on day 10 at 5mg and didn’t last. I asked the second rheum if perhaps 20mg wasn’t enough and he said he has lupus patients see improvement with as low a dose as 5mg. |
| 58 | 36 | 1 | 'diet', 'kidney', 'renal', 'op', 'harm', 'apple', 'myth', 'pie', 'protein', 'disease', 'carnivore', 'dialysis', 'patients', 'diets', 'kidneys', 'consequences', 'research', 'applicable', 'cinnamon', 'known' | Well, considering you get a very limited amount of wiggle room with kidney damage and you can’t live without them ♀. That’s not being negative. That’s being realistic and ethical. is the response to the OP statement about carnivore diet. |
| 59 | 36 | 1 | 'wake', 'chronic', 'major', 'ruin', 'bored', 'creep', 'scattered', 'migraines', 'hahaha', 'friends', 'progressively', 'soreness', 'yr', 'hrs', 'days', 'resting', 'suck', 'day', 'enough', 'triggered' | You immediately vSometimes my whole body hurts for seemingly no reason, or I did something physically involved the day before. Joint pain, too. But yeah. You have antibodies attacking your tissues throughout your body. Joints, organs, muscles, brain, face. It's more unusual if it doesn't cause pain. |
| 60 | 36 | 5 | 'nerve', 'pain', 'take', 'mg', 'gabapentine', 'antidepressants', 'toroidal', 'lyrica', 'dosage', 'aleve', 'meds', 'toradol', 'lasts', 'depression', 'taking', 'help', 'injection', 'prescribe', 'steroids', 'rebounded' | I can't take tylenol or Nsaids because I have kidney and liver involvement. Plus, the amount I would need to take of over the counter meds would damage my kidney and liver. So I see a pain doctor once every 2 months. |
| 61 | 35 | 4 | 'winter', 'heat', 'seattle', 'cold', 'winters', 'live', 'car', 'washington', 'summer', 'state', 'rainy', 'outside', 'sc', 'fl', 'love', 'lived', 'moved', 'weather', 'move', 'property' | Summer and winter are both difficult for me. I live in Massachusetts so it's not too bad. Winter sucks but I guess one upside of climate change is that winter is more mild now. I don't think I could live anywhere with more extreme heat or cold. |
| 62 | 35 | 2 | 'pregnancy', 'baby', 'son', 'transfusion', 'born', 'platelet', 'denial', 'obgyn', 'shingles', 'block', 'needed', 'fortunately', 'heart', 'platelets', 'mentioned', 'time', 'wanted', 'fetal', 'oz', 'hoarse' | u/Longjumping_Plan_829 Would you mind sharing what types of complications you experienced with pregnancy? I suspect I may have a similar diagnosis and have started family planning to look into what I may be a carrier of, but I think I should look into seeing a rheumatologist as well. My symptoms seem to fluctuate so I keep thinking its just stress related, but they are quite similar to what many others have mentioned: dry eyes, dry mouth, throat feels swollen to the point its difficult to swallow, hoarse voice, joint pain, molted skin pattern. Ugh, I’m sorry to hear. For me, my pregnancy was not planned. |
| 63 | 35 | 5 | 'digestive', 'ibd', 'issues', 'ibs', 'gastro', 'celiac', 'gi', 'lactose', 'stomach', 'gut', 'lupus', 'acid', 'colonoscopy', 'fecal', 'related', 'tract', 'eat', 'system', 'cellcept', 'gastroparesis' | Lupus really rarely affects the digestive tract. My rheum said it only happens when someone is so sick with it attacking multiple organs that they're in the hospital in a super severe flare. It goes after kidney, heart and lungs long before it will attack your GI system, so it sounds like it's not the cause of your constipation (since you're not fully diagnosed, I'm assuming you don't have all those affected). |
| 64 | 34 | 1 | 'gluten', 'wheat', 'celiac', 'stomach', 'free', 'diet', 'tomatoes', 'allergy', 'food', 'corn', 'triggers', 'foods', 'eat', 'cheese', 'rice', 'allergist', 'issues', 'eating', 'broth', 'eggplant' | Yep I have an allergist and had an allergy panel done a little over a year ago. I have a few tree pollen allergies and a reaction to nickel but that's it. Well shoot! I was hoping you could give up cheese and start feeling better. I'd sooooo feel worse if I had to give up cheese! lol When doctors won’t help you can try the Elimination diet. |
| 65 | 34 | 5 | 'methotrexate', 'leucovorin', 'tablets', 'plaquenil', 'swallow', 'mg', 'im', 'pills', 'dose', 'cellcept', 'effects', 'take', 'signals', 'underweight', 'hungry', 'side', 'put', 'twelve', 'nauseous', 'total' | I take my meds. But I don’t want to. They aren’t without side effects. I have a whole slew of health manifestations having nothing to do with lupus that have risen from taking them. Methotrexate for instance. I was on it for half a year. |
| 66 | 34 | 1 | 'wbc', 'lymphocytes', 'low', 'typically', 'high', 'count', 'explained', 'undifferentiated', 'criteria', 'infection', 'usually', 'eats', 'satisfy', 'lupus', 'lymphocytopenia', 'true', 'whining', 'paired', 'counts', 'specific' | True, and I understand. I guess I find it weird the low WBC is explained as stress or infection, which could be true, but it's also one of the diagnostic criteria for lupus, which when paired with all the other stuff I've got going on, would satisfy the criteria for a diagnosis. I guess I'm just complaining (whining) that I wasn't given a specific name, even undifferentiated, and specific criteria (low WBC) is being explained as something else and not an indicator of lupus or other autoimmune condition. |
| 67 | 32 | 1 | 'criteria', 'diagnostic', 'wiki', 'link', 'page', 'top', 'score', 'dx', 'scroll', 'post', 'heds', 'comment', 'list', 'points', 'wonderingthat', 'tests', 'pinned', 'incl', 'lupus', 'see' | The diagnostic criteria for lupus can be found if you scroll up. Most of your symptoms could be too many things and are not used as criteria for lupus. |
| 68 | 30 | 1 | 'aip', 'gnavus', 'book', 'diet', 'starch', 'resistant', 'foods', 'bacteria', 'gut', 'kriegel', 'cookbook', 'intestinal', 'restrictive', 'amy', 'antigens', 'reduce', 'produced', 'food', 'research', 'microbiome' | Having a better diet has been the biggest help for me. Unfortunately not everyone is that lucky and has to rely on meds, buy for some it can be quite life changing! AIP tamed my lupus too, and I've been very watchful on this sub for people saying "AIP didn't work for me", but nobody seems to say that. I do see people saying, "it's hard to adhere to AIP", which I agree with. But I am definitely not seeing people talking about AIP failing them. I evangelize about AIP, but I feel somewhat conflicted to do so because I would feel crappy if I suggested AIP and AIP was ineffective. But I'm still waiting to see someone who says that they stuck with it for 3-6 months and it didn't help a lot. |
| 69 | 30 | 1 | 'tattoo', 'tattoos', 'ink', 'cellcept', 'healed', 'itchy', 'artist', 'raised', 'saniderm', 'tattooed', 'heal', 'wrist', 'got', 'fine', 'gets', 'folliculitis', 'taking', 'prednisone', 'black', 'helps' | During flare up my tattoos try to leave my body or I think my body tries to push the ink out, especially the red ink. They get really itchy. This is such a wild feeling but I 100% agree. Oh my god so THATS WHAT THAT IS. One of my tattoos swells and gets so itchy in some areas - mostly the part of it right above the side of my outer wrist/on top of top of wrist and that’s where the red ink is (also the YELLOW ink does it the worst for me) that entire section of it is raised and in the sun it gets worse and burn-itchy. Now this all makes sense. |
| 70 | 29 | 1 | 'recommended', 'nioxin', 'using', 'hair', 'rogaine', 'loss', 'hahai', 'shampoo', 'boat', 'route', 'grandma', 'chemothank', 'buy', 'thatalso', 'stopped', 'nope', 'didn', 'newly', 'figured', 'chemo' | I’m so sorry! I was in that boat 4 years ago. I used nioxin religiously and it actually resolved it pretty quickly, which is for the best because that stuff is expensive. I came here to say Nioxin appeared to solve my problems too. Did it stay even after you stopped using it? I only had hair loss about four years ago when I was newly diagnosed and I haven’t had hair loss since, not sure if the shampoo had a hand in that. |
| 71 | 29 | 1 | 'laugh', 'lazy', 'line', 'roll', 'immune', 'someone', 'cure', 'win', 'usually', 'eye', 'wired', 'haha', 'lupavita', 'heckle', 'looooool', 'made', 'ashwaganga', 'lupus', 'oy', 'lose' | Wow, wouldn't that be nice. As I type from bed on a Wed afternoon because I'm too exhausted to do anything without a nap first. But I'm not rashing right now so I guess I'm just lazy. Ugh. UUUHHGGGG. Anytime someone calls me lazy I say, “L is for lupus not lazy”. That usually shuts people up (and a laugh). |
| 72 | 29 | 1 | 'diet', 'food', 'eat', 'cure', 'judge', 'mediterranean', 'eating', 'people', 'vegan', 'feel', 'cured', 'projection', 'pasta', 'silently', 'foods', 'processed', 'disease', 'climate', 'choosing', 'whatsoever' | When I came out as having lupus to my friends my one friend said “oh my friend cured her chrons disease, I bet you can too!” I mean. just some sympathy would do, I don’t want to get into how it’s progressive and yes some people can put their flares into remission but it doesn’t work that way, so I just responded “yeah maybe” I’m not educated enough to have discourse about it but it did feel pretty dismissive. That one made me feel pretty bad, and how I wish more of my friends asked or talked to me about it. I am lucky that few did feel confident in asking more about it in person, that did make me feel heard. It’s just a big deal to me, obviously I always hate the “I know someone who cured their autoimmune disease by cutting this entire food group out of their diet!”I know a lot of people talk about the anti-inflammatory diet or whatever, but for me, I have tried … and it makes no difference whatsoever. |
| 73 | 29 | 1 | 'celery', 'juice', 'moss', 'sea', 'joy', 'sarcasm', 'pressed', 'kills', 'oils', 'essential', 'healing', 'stupid', 'omg', 'clean', 'companies', 'videos', 'bodies', 'defense', 'misinformation', 'beneficial' | u/Suspicious_Yogurt553, your comment was removed - only users flaired as SLE, UCTD/MCTD or CLD/DLE Diagnosed can comment on Diagnosed Users Only flaired posts. How to flair yourself here. I am a bot, and this action was performed automatically. |
| 74 | 28 | 0 | 'imitator', 'relief', 'lupus', 'dermatologist', 'symptoms', 'could', 'open', 'go', 'diseases', 'malaise', 'get', 'goal', 'called', 'rashes', 'bottom', 'proper', 'getting', 'rash', 'great', 'reason' | There are a hundred reasons for hair loss. Statistically speaking lupus is going to be close to the bottom of that list for a 16 years old male. You seem fixated on lupus as the cause. |
| 75 | 28 | 3 | 'cane', 'hands', 'tmi', 'grip', 'tight', 'swell', 'toilet', 'sucks', 'bras', 'bandaid', 'metal', 'underwear', 'hand', 'pants', 'pulling', 'effecting', 'stabbing', 'gotta', 'ice', 'bad' | That happens unfortunately :( if you get it in your lower body at all, I’d recommend using a cane. I just got one and it’s made a world of difference. I have a 14 months old, she would probably use it as a weapon LMAO! a worthy risk maybe!Yeah maybe. My hands are so bad idk if I could grip a cane, tho. TMI, but just pulling my underwear up from getting off the toilet is excruciating I am so sorry! That is just awful! Not TMI at all, we can share this stuff with each other! Much love and support Thank you so much! Same to you! Same! It sucks so much! Mine swell like that and I get stabbing pain frequently, especially with movement. |
| 76 | 28 | 0 | 'arthritis', 'joint', 'sle', 'typically', 'visible', 'mornings', 'cause', 'pain', 'mutation', 'perse', 'bilaterally', 'damage', 'description', 'non', 'autoinflammatory', 'ruled', 'moot', 'erosive', 'verbal', 'lifting' | Depends on what the value for anti-smith, specifically, is. If it’s borderline as well, a rheumatologist may do watchful waiting. However, if it’s really elevated, like 2-3 times max cutoff, that’s more the realm we’re talking about for lupus. The SM measure was 1. 4. |
| 77 | 27 | 3 | 'georgia', 'massachusetts', 'mask', 'ga', 'public', 'data', 'covid', 'masking', 'pandemic', 'collective', 'hampshire', 'vermont', 'deaths', 'others', 'maine', 'comply', 'professionals', 'vulnerable', 'surges', 'people' | Oh I believe public health is a collective effort. But there should be no anticipation or expectation of the public to conform to a mandate that hasn’t been in place for months and even years in some places. Is it frustrating yea. Is it enforceable, no. |
| 78 | 27 | 2 | 'postpartum', 'pregnancy', 'canker', 'underlying', 'questions', 'removed', 'sores', 'lenny', 'chica', 'hormones', 'ulcers', 'dismissive', 'ima', 'lupusbad', 'ups', 'persistent', 'wiki', 'submit', 'link', 'approve' | u/lenny-chica Your question was removed because diagnosis questions are limited to 400 words. Shorter questions get more feedback. Note: Editing your removed post won't approve it, you need to re-submit it. I am a bot, and this action was performed automatically. |
| 79 | 27 | 1 | 'hair', 'shaved', 'wigs', 'bald', 'shave', 'head', 'lost', 'wigsbytiffani', 'grew', 'falling', 'human', 'teeth', 'loss', 'showers', 'ever', 'beautiful', 'short', 'clumps', 'hot', 'wig' | Hi there! I am a 25 years old female that shaved my head for the first time this week. It was devastating even though I had been planning on it for over a year as the bald spots grew and grew. I also had really long/thick hair my whole life up until two years ago as it began to thin. Even when it was thinning I would never have imagined it turning into alopecia. |
| 80 | 27 | 1 | 'crystals', 'ass', 'rocks', 'shoved', 'mybodyisatempleok', 'leisurely', 'clenching', 'indiana', 'treasure', 'jones', 'bedin', 'walks', 'butt', 'improved', 'hindu', 'full', 'restart', 'shoving', 'moon', 'crystal' | You’re gonna have to restart. It only works if you do it all at once while listening to high frequency meditation music. Ugh, my mistake was that I shoved 27 crystals up my ass… one crystal too many I guess It’s fine. Just go for a walk. Absolutely. Leisurely walks while clenching rocks in my butt has really improved my fatigue levels! #mybodyisatempleOk Indiana Jones and the lost treasure i need to go to bed. In the full sun, don’t forget! |
| 81 | 25 | 1 | 'ebv', 'via', 'barr', 'virus', 'epstein', 'adults', 'tthank', 'breast', 'infection', 'accurately', 'detect', 'measure', 'tested', 'ms', 'mono', 'kissing', 'directly', 'welldr', 'intercourse', 'virtually' | Go ask your doctor for an Epstein-Barr Virus (EBV) panel, it can measure more accurately detect whether you’ve had a natural infection. But you’re too young and healthy looking to be sick! |
| 82 | 25 | 2 | 'gaslighting', 'victim', 'comment', 'medical', 'ethics', 'op', 'term', 'harm', 'reality', 'gaslight', 'ignorant', 'sanction', 'healthcare', 'correct', 'isn', 'reprimand', 'unbelievable', 'loose', 'gaslit', 'violation' | Just as a internet/text note, when you put things in quotes when it isn’t obviously someone else’s direct words, it comes off as sarcastic. Are you looking for pain relief information or information as to why certain symptoms seemed to catch her attention more than others?I’ll be sure to remove the quotes. Didn’t think it was that seriousWe get a lot of posters who are convinced they are being gaslighted (they aren’t, they’re just ignorant of how the medical system works). There’s a ton of underserved shit piled on healthcare that is due to the general public’s astonishing level of ignorance. |
| 83 | 25 | 5 | 'stomach', 'ibs', 'acupuncture', 'regulating', 'ease', 'mites', 'acids', 'allergy', 'allergic', 'particular', 'reducing', 'grows', 'grass', 'area', 'dust', 'yo', 'shocked', 'clue', 'neither', 'thinka' | That's reassuring coming from you, I'll definitely look into it! Might be weird to ask but which doctor would I even bring this up to? A GP? Or would I have to look into something else? I would go to your GP and see if they recommend a particular specialized doctor. There are some who specialize in allergy and food sensitivities. When I was younger I got sent to a particular doctor to get allergy testing done on me. It was a bitch being young and not being able to scratch. |
| 84 | 24 | 3 | 'bra', 'swimming', 'mono', 'thought', 'natural', 'wear', 'monospot', 'flu', 'always', 'oranges', 'rollerblading', 'baggy', 'mermaid', 'drier', 'parents', 'detergents', 'tended', 'panties', 'elastic', 'tasted' | Sometimes test results and pictures are all you have to prove you're not making things up. By the time I finally got to my rheumatologist I had my chronological history down, with pictures and test results. It took her about 3 minutes after to confirm it's lupus. |
| 85 | 24 | 1 | 'marrow', 'bone', 'hospital', 'travel', 'medicine', 'blood', 'imuran', 'count', 'different', 'tmpt', 'orlando', 'queue', 'transferred', 'nonchalantly', 'register', 'sailing', 'cookie', 'allergic', 'discharged', 'traveling' | Whenever I’m in the sun too long I get all hot and prickly feeling! I thought that just meant it was hot out! Talk to your rheum, it’s different for everyone. I’m allergic to hydroxychloroquine, so the only lupus medication I’m on is imuran. I’ve personally done very well on it. |
| 86 | 24 | 3 | 'dizzy', 'far', 'woozy', 'belly', 'spared', 'though', 'swimming', 'hoping', 'read', 'front', 'tonight', 'dizziness', 'report', 'passes', 'effects', 'bathroom', 'hard', 'friday', 'med', 'close' | I’m on my 3rd dose today! I held off but decided I needed to be a functional person - on hydroxychloroquine for a month now too. How have you been so far? So I had no side effects the first few days, and felt fine. Today I’m a little dizzy but no other effects. Hoping it passes…Dizzy though…autoimmune or medicine…hard to say, lol. |
| 87 | 23 | 5 | 'pleurisy', 'chest', 'pericarditis', 'effusions', 'pleural', 'stabbing', 'membrane', 'pain', 'heartbeats', 'laying', 'icepicked', 'lungs', 'flat', 'breath', 'glide', 'worse', 'ribs', 'builds', 'surrounds', 'could' | It's like a dull stabbing pain. I'm not sure what triggers it to be honest. The pain sometimes radiates to the back of my shoulder too. I don't find anything makes it better, it just eventually goes away. It honestly could be many things. The typical chest pain from lupus is either pleurisy, which is worse with chest wall movement (like breathing), or pericarditis, which is typically described as stabbing pain with heartbeats made worse by laying flat. |
| 88 | 23 | 3 | 'students', 'law', 'school', 'elementary', 'job', 'teaching', 'professors', 'college', 'mobility', 'teach', 'classes', 'week', 'impossible', 'university', 'teacher', 'music', 'part', 'time', 'accommodations', 'flare' | I’m finishing my Master’s in Education (MSEd) in May and had I known my disease would get this bad I would not have gone into education :/I know this is an old post, but could you share why education isn’t a good fit for you with lupus?It’s okay. So I don’t only have SLE, I have Rheumatoid Arthritis. I’m on a lot of different drugs including a biologic that makes me really prone to infection. Between flares, pain, mobility issues (particularly my knees and joints from my wrist up), and the amount of infections I get, being around students causes me to get whatever bugs or illnesses they have, and students always have something, usually a respiratory illness. |
| 89 | 23 | 5 | 'prednisone', 'tictacs', 'smiling', 'shell', 'devil', 'bothers', 'rituxan', 'wow', 'tube', 'human', 'heard', 'remember', 'arosethank', 'afraid', 'putting', 'rtxer', 'flare', 'actually', 'plaquenal', 'ziploc' | Yeah, prednisone is the devil’s tictacs. I went from being a shell of a human to actually smiling in 12 hours (and 40mg). |
| 90 | 23 | 2 | 'uctd', 'strict', 'criteria', 'diagnosis', 'ana', 'dx', 'psoriatic', 'vs', 'kidney', 'disease', 'dsdna', 'different', 'arthritis', 'diagnosed', 'positive', 'yetright', 'pots', 'connective', 'undiagnosed', 'clinical' | when they say mild they usually are referring to symptom progression (like to major organ involvement with damage potential like nephritis in the kidneys), not necessarily pain levels or the number of body areas affected. About ~1/3 of people with UCTD will spontaneously go into remission within a few years, another ~1/3 just have UCTD forever, and ~1/4 will progress to having a differentiated connective tissue disease. The implications of having one diagnosis over the other are incredibly different (take the difference in the 5 years mortality rate, for instance). Both UCTD and MCTD are painful, taxing, soul-sucking conditions. That just isn’t what the mild descriptor is referring to. I hope that makes sense. |
| 91 | 22 | 1 | 'depression', 'neuropsychiatric', 'fib', 'cns', 'dangerous', 'seizures', 'npsle', 'brain', 'mental', 'rare', 'yea', 'heard', 'psychosis', 'mri', 'wires', 'cardiomegaly', 'infarctions', 'glued', 'spooked', 'involvement' | What is this? I had. a MRI with contrast yesterday but for a different reason. I have a pinel cyst that I have been following. Can they see what you are talking about from my recent MRI? Ty!!I’m not sure what they can or can’t see but there’s like brain scarring and inflammation within the called but I hate itMy neuro said it’s called NPSLE - neuropsychiatric SLE. I have it too and my seizures started in 2019. |
| 92 | 21 | 2 | 'question', 'us', 'removed', 'chatgpt', 'exhaustive', 'summarize', 'paste', 'breakdown', 'questions', 'post', 'guidance', 'childhood', 'submit', 'approve', 'editing', 'shorter', 'difficulty', 'limited', 'words', 'moderators' | u/weeookleoo Your question was removed because diagnosis questions are limited to 375 words. Use ChatGPT to help summarize your question if you are having difficulty making your question shorter. Note: Editing your removed post won't approve it, you need to re-submit it. |
| 93 | 21 | 3 | 'teacher', 'school', 'job', 'home', 'kids', 'assistant', 'disability', 'full', 'mas', 'easiest', 'tired', 'virtual', 'ma', 'physio', 'difficult', 'chef', 'summers', 'careers', 'work', 'hs' | I have been in the process of finding doctors who speak English, but since finding someone who can do that AND diagnose whatever I have is rare, my choices are limited. Well, I don't feel lucky with everything else that's been happening to me! I’m a teacher, I was diagnosed in my first year. Pretty crazy experience. |
| 94 | 21 | 3 | 'tendon', 'fallback', 'affecting', 'option', 'forward', 'moving', 'taking', 'days', 'sucks', 'work', 'suitable', 'fairness', 'threatened', 'tee', 'outside', 'achilles', 'fired', 'absence', 'made', 'plan' | I don't go outside as much as I used to, I thought it was outside activity wearing me out, or heat, never considered it could be the actual sun. I used to love being outside, fortunately I also love video games so I had a fallback plan :-/Did you feel crazy going through getting diagnosed? I just want this goose chase for why I'm feeling like garbage to be over with already. I seriously wonder if I'm crazy sometimes with the symptoms, especially since some days I'm alright and other days I can be totally wrecked :(Edit: and thank you for the kind response and words of encouragement. I really needed it. 用户已删除评论Holy crap, I didn't realize tendon inflammation was related? Add that to the list then I guess. I've had a flare up in my Achilles' tendon about 2 months ago (made some sense, the urgent care said they thought it happened from driving), and off and on it keeps affecting my neck, making it hard to move my head around. |
| 95 | 21 | 1 | 'safe', 'ofl', 'letting', 'mistakes', 'saved', 'instead', 'inflamed', 'doctor', 'sufferthis', 'misconception', 'organ', 'surely', 'person', 'relieved', 'samples', 'damage', 'monitoring', 'treat', 'tell', 'bad' | Nope. Never pressure the doctor, just talk to the doctor about it Tell the Dr. go ahead and treat you for it and see if it helps. |
| 96 | 20 | 1 | 'misdiagnosis', 'language', 'hypothyroidism', 'lucky', 'cayce', 'clairvoyant', 'edgar', 'speak', 'wdym', 'moronic', 'finding', 'ignorance', 'beginnings', 'discovered', 'gaslight', 'native', 'english', 'flu', 'pure', 'doesnt' | Yes, it could be lupus. It could also be hypothyroidism. If I lived in a foreign country that didn't speak my native language, I would find a doctor that spoke my language. We have them in the US as well. What I believe brought on lupus for me was a strep infection that wouldn't go away. I also had beginnings of hypothyroidism that started not long before that. |
| 97 | 20 | 2 | 'criteria', 'diagnostic', 'wiki', 'link', 'familiarize', 'see', 'part', 'post', 'heartbreak', 'terror', 'book', 'cookbook', 'lp', 'zinc', 'diagnosis', 'list', 'ty', 'pinned', 'code', 'limit' | You can see the diagnostic criteria for Lupus under the Wiki link in this post. |
| 98 | 20 | 3 | 'socks', 'compression', 'calves', 'legs', 'pac', 'amazon', 'feet', 'felt', 'ankles', 'wear', 'hose', 'tight', 'spots', 'bit', 'dam', 'noticed', 'poke', 'swelling', 'adorable', 'steroids' | Second this and I do compression leggings. Just went to stupid Walmart NOT wearing them and made me realize HOW MUCH THEY HELP! Oof! I bought them to fly years ago and now I wear them at least five days a week. On Amazon there are such awesome / fun designs. I have to watch out though, I have found my family stealing my socks, lol. I typically buy knee high socks. I know! I’ve gotten soooo many compliments when I’ve worn my crazy print compression socks to work! I just got some Pac-Man ones not long ago Pac-Man? Do you have a link to share? I’ve got to try and remember the dam site I ordered them from, as it wasn’t from Amazon. |
| **Weibo** | | | | |
| 0 | 1997 | 1 | artemisinin', 'team', 'hope', 'break through', 'treat', 'great', 'scientist', 'salute', 'Really', 'Lupus erythematosus', 'thanks', 'notice', 'Awesome', 'scientific research', 'enlarge', 'human', 'Pride', 'news', 'grandma', 'expect' | Tu You you!!! |
| 1 | 1917 | 5 | oneself', 'time', 'Mom', 'children', 'We', 'today', 'think', 'know', 'of one's own unit', 'they', 'love', 'parent', 'hospital', 'hope', 'later', 'mother', 'Really', 'Is', 'family', 'life' | It's two o'clock in the morning. I'm very tired today. I wanted to have a good sleep. I'll start a new day tomorrow when I'm full of spirit. Maybe it's because of drugs, maybe it's because of mental state, or I'm sleepless. Let's write down some memories of 2023. I fell ill and seemed to be isolated from the real world. Many things could not be clearly recalled in my mind. In December 2022, I went to a favorite clothing company as a shopping guide. Even if I had no sales experience, even if I was honest and could not talk and promote at work, I would look forward to my favorite brand and style design. The music and scented tea in the store are also my favorite style and taste. In January, I quit my job as a shopping guide and began to prepare for the provincial examination. The days when I go to class every day are also very regular. From 9 a.m. to 10 p.m., I have also been lazy, but I feel like studying every day, which can at least reduce some anxiety and make some progress. However, with my current learning level, I cannot pass the provincial examination. In May, I decided to have my own nest, so I spent all my savings and sold my camera and Xiaoyuan's physical album. Now I still feel very reluctant to part with it. I bought a guitar that doesn't tune. I don't want to take it home anymore, and I don't like it. In June, I participated in the joint examination of Xingyi public institutions. I may have failed too many times. This examination also focused on participation, but I was surprised. I didn't add 2 points, which was also an oversight. So on the recommendation of my friend, I went to Beijing Qiansheng and met a humorous interview teacher and fellow editor. I didn't go out much and never knew how excellent others were. I also understood that all success required perseverance and unremitting efforts. When we came to the face-to-face test, we all cut through difficulties. I was the one with better luck and shorter time. But in the end, I failed to counter attack. Although the day before the interview, all the interviewing teachers told Wo that I was doing well and that I could counter attack successfully if the difference was not large, but the reality was that the difference was also large and the opponent was excellent. I told myself, "it is hard to avoid dark and cool at night, and there must be dawn ahead". In July, in order to maintain my livelihood, I continued to find a job as a shopping guide. My colleagues and sisters took good care of me, so I worked until December with a salary of less than 2K per month. The reason for leaving my job was that I could not take leave on weekends. In order to take exams on weekends in the future, I ended this unsuitable job. I almost forgot that I went to Guiyang to watch the music festival with my sisters in July. After three years, I finally saw prism and finally heard "this is the bravest moment in my life" at dusk; ZHAOLEI's scene is really worth watching over and over again; I also heard Xu Song in my youth and a simple tree like summer flowers. 8. Until the end of my resignation in December, my fate gave me another heavy blow. It was just a cold and cough, but it could not be cured by repeated attacks. Later, it became more and more serious, and my general weakness began to vomit and diarrhea. So I went to the emergency room. For the first time in my life, I took an ambulance, lay on a stretcher without consciousness, and went to the emergency room for the first time. I was afraid, and my life was over. Xiao Guo, sister Wei, Dapeng and general manager Liao came to the hospital to visit me at the first time. My mother and brother also accompanied me throughout the whole process. I seemed to be less scared. On the first day of hospitalization, December 28, the doctor told me that systemic lupus erythematosus was diagnosed, involving multiple organs. Most of the organs had started to fail, and must be treated as soon as possible, otherwise I might lose my life. Hearing this news, my mind had started to blank, like suddenly entering another world, I seemed to have died. Next, if I could continue to live, it would be another life. During the hospitalization, everyone worked very hard. For medical expenses, to encourage me and take care of me, especially my mother, she has hardly closed her eyes since she was ill, but she has been comforting me. As long as I am alive, everything else is not important. Sister Qin came from Ziyun all night and took care of me. I didn't sleep a wink all night. She rushed back to work the next morning. Sister Lian and her old employers came as soon as they knew the news. This made me understand that I can't live up to everyone's care and expectations. I must listen to the doctor, treat well and live well. After 15 days, I was finally discharged from the hospital. Seeing the good and evil of human nature, I knew that I had lost the right to live a normal life. Many things may not be done in my life, such as getting married and having children. Although the doctor always told me not to give up myself, I would get better. Like ordinary people, I would continue to work, get married and have children. But I am still ready for the impossible. I really have never thought of dragging anyone down. Only then do I understand that there may be no love in this world, there may be, but it will not happen to me. I told myself, "it's good to be loved, it doesn't matter.". |
| 2 | 1576 | 1 | Systematic', 'Lupus erythematosus', 'Can't sleep', 'happy', 'feel', 'oneself', 'review', 'insomnia', 'Urine protein', 'tomorrow', 'come on', 'think', 'hope', 'happy new year', 'recently', 'Really', 'life', 'Happy', 'today', 'know' | I hope we are all healthy and happy |
| 3 | 1545 | 1 | Systematic', 'Yes no', 'sister', 'hormone', 'Lupus erythematosus', 'hair', 'once', 'fun', 'now', 'everybody', 'excuse me', 'pose a question', 'recently', 'feel', 'You', 'What to do', 'Metoprolol', 'physical examination', 'Hair loss', 'Isn't it' | I would like to ask if there is any sister with nerve injury due to lupus |
| 4 | 1542 | 2 | mother and daughter', 'Shuangshuang', 'Zhejiang', 'diagnose', 'Get well soon', 'beauty', 'Frightening', 'Lupus erythematosus', 'Read', 'hope', 'inheritance', 'Good health', 'ydl', 'This is', 'dreadful', 'safe and peaceful', 'Oh, my God', 'safety', 'terror', 'woman' | My God |
| 5 | 1227 | 1 | Lupus erythematosus', 'dreadful', 'hope', 'No', 'Really', 'Awesome', 'skin disease', 'Dangerous', 'Frightening', 'Immune disease', 'Radical cure', 'cure', 'Popular science', 'terror', 'Can treat', 'understand', 'once', 'early', 'This is', 'gospel' | If you don't understand, just ask what the disease is |
| 6 | 1168 | 0 | platelet', 'ovary', 'nephropathy', 'reduce', 'kidney', 'embryo', 'red blood cell', 'glomerulus', 'abnormal', 'white blood cell', 'abortion', 'uterus', 'factor', 'cause', 'increase', 'Premature senility', 'anemia', 'reason', 'hypertension', 'Kidney disease' | Blood is mainly composed of plasma and blood cells, and the focus of blood routine examination is to check the blood cell part; According to the results of these three kinds of cells in blood routine examination, we can roughly determine the health of blood, and then determine the health of the body. Red blood cells "↓" indicates that the main component of anemia red blood cells is hemoglobin. Once the value of red blood cells is low, it is equivalent to less hemoglobin. At this time, the blood quality will be affected, the ability to transport oxygen will also become weak, and anemia symptoms will easily occur, making people look pale; Once anemic for a long time, it will lead to heart disease, and even affect the nervous system and brain. This may be caused by three major reasons. Generation reduction. Decreased erythropoiesis may be a problem of normal bone marrow hematopoietic capacity, including leukemia, aplastic anemia, etc; Lost more. If the production of red blood cells is normal, it is necessary to suspect whether there is outward loss, such as hemorrhoids, hematuria, gastrointestinal bleeding, gastrointestinal diseases, etc; Women's excessive menstrual volume will also lead to excessive blood loss, which will lead to red blood cell reduction and anemia. Much damage. Some people are sensitive to some anti-inflammatory drugs, antipyretics and analgesics, and their blood tissues are damaged; Autoimmune diseases may also cause the autoimmune system to "kill" red blood cells. Red blood cells "↑" may be pathological, which may have pathological reasons. The most common is bone marrow proliferative tumor, which will cause excessive production of red blood cells from bone marrow. White blood cells "↓" immune decline white blood cells are the general name of five kinds of cells, namely neutrophils, lymphocytes, monocytes, eosinophils and basophils. Only when there are enough white blood cells in the body, can they have normal ability to resist bacterial or viral attack. If white blood cells are lower than the normal range, especially neutrophils are lower than 1000/ microliter, human resistance is low; If white blood cells are severely reduced, the probability of infection increases significantly. Excluding genetic factors, there are generally four common causes of leukopenia: one is that bone marrow disease leads to leukopenia and maturation disorders; Second, taking antipyretic analgesics, sulfonamides, anti tuberculosis drugs, hyperthyroidism drugs and other drugs; Third, viral infection, such as influenza and hepatitis B virus infection; In addition, some patients have pseudoleukopenia, which is caused by the retention of leukocytes in the circulating pool. In fact, the total number of leukocytes in the body is not low. Leukocyte "↑" and infection related acute infection and suppurative inflammation caused by various cocci, such as otitis media, tonsillitis, appendicitis, abscess, etc; Systemic infections such as pneumonia and sepsis, uremia and malignant tumors can all lead to leukocytosis. If the influence of leukemia and other disease factors is excluded, the platelet is low but the degree is not high, and the patient has no bleeding performance, so it does not necessarily need treatment. When platelets are less than 60000 / microliter, there is a risk of bleeding or infection. High platelets are easy to form thrombus. Too high platelets will affect blood flow. When the blood flow speed is slow, it is easy to have thrombus, which will lead to myocardial infarction and cerebral infarction, which is life-threatening. Thrombocytosis caused by myeloproliferative neoplasms is also accompanied by abnormal platelet function. Patients may have bleeding, which also needs attention. |
| 7 | 999 | 1 | sle', 'lupus', 'patient', 'symptom', 'erythema', 'appear', 'Condition', 'skin', 'Lupus erythematosus', 'damage', 'system', 'woman', 'Systematic', 'rash', 'self', 'factor', 'one kind', 'avoid', 'organ', 'performance' | Lupus erythematosus, with the medical name of "systemic lupus erythematosus", is an autoimmune disease. This disease can cause a large number of pathogenic autoantibodies and immune complexes in the body, causing damage to multiple systems and organs of the whole body. There is a significant gender difference in systemic lupus erythematosus. The incidence in women is about 9 times that in men, especially in young women. 1. fatigue. This is one of the most common symptoms of systemic lupus erythematosus, and patients often feel tired that is difficult to relieve. 2. joint pain and swelling. Although these symptoms may be caused by multiple factors, they are also early signs of systemic lupus erythematosus. 3. low fever. In the early stage of systemic lupus erythematosus, there may be low fever without obvious cause. 4. rash. 5. photoallergy. Obvious skin allergic reaction occurred after sunlight exposure. 6. sparse hair. Patients with systemic lupus erythematosus may experience hair thinning or massive hair loss. 7. discoloration of fingers and toes. In cold weather or under pressure, fingers and toes may turn white, blue or red due to Raynaud's phenomenon (refers to sudden spasm of arterioles at the end of fingers (toes) under the stimulation of cold or emotional tension, resulting in changes in skin color). 8. oral ulcer. 9. hematuria and proteinuria. Systemic lupus erythematosus is prone to cause lupus renal damage, which can appear hematuria, foamy urine and other urine changes. 10. infertility and recurrent miscarriage. When the condition of female patients with systemic lupus erythematosus is poorly controlled, they are not easy to get pregnant and prone to adverse pregnancy events such as recurrent abortion. If the above manifestations occur in the high-risk population of lupus erythematosus, it is recommended to seek medical advice in time, identify the causes and treat symptomatically. Tongqiang said that the etiology of systemic lupus erythematosus is very complex. In order to reduce the risk of disease onset, high-risk people need to maintain a healthy lifestyle, including a balanced diet, regular exercise and keeping a happy mood, in addition to avoiding excessive sun exposure or UV exposure. |
| 8 | 858 | 3 | Water drop', 'easy', 'A kind person', 'transmit', 'everybody', 'love', 'Help', 'Help', 'Please', 'link', 'everybody', 'Home stay', 'Physical illness', 'help me', 'go out', 'this year', 'Help', 'daughter', 'This is', 'hope' | Hello everyone! My name is chenjianping. My family lives in Haishu District, Ningbo City, Zhejiang Province. My daughter's name is ... please help me and forward it to more kind people. |
| 9 | 669 | 3 | lena', 'selena', 'gomez', 'francia', 'gomez', 'Best friend', 'Bieber', 'Friends', 'raisa', 'Kidney transplant', 'Fans', 'Transplantation', 'accept', 'matching', 'selenagomez', 'experience', 'oneself', 'star', 'friend', 'One' | Recently, selenagomez revealed in INS' long article that she had received a kidney transplant before, and her friend franciaraisa donated a kidney, which is also the reason why she had not come out to promote the new music before. Because of lupus erythematosus, Selena had to transplant a kidney. Selena expressed her gratitude to her family and the doctor who operated on her, and also to franciaraisa who made such a sacrifice for her. |
| 10 | 666 | 2 | Jackson', 'Michael', 'mj', 'bleach', 'Rumor mongering', 'Vitiligo', 'colour', 'torment', 'gaga', 'rumour', 'lady', 'Suffer from', 'media', 'dreadful', 'Lupus erythematosus', 'Black', 'nose', 'many years', 'Plainly', 'Superstar' | Ah? So is he? |
| 11 | 516 | 2 | Marathon', 'Disease', 'confrontation', 'woman', 'mislead', 'motion', 'come on', 'tired', 'No', 'Bask in the sun', 'Lupus erythematosus', 'Running', 'insist on', 'news', 'victory', 'Exercise', 'overcome', 'Inspirational', 'such' | Great great |
| 12 | 484 | 1 | Headline', 'article', 'release', 'publish', 'stem cells', 'Reprint', 'Bowen', 'An article', 'Lupus erythematosus', 'Systematic', 'treat', 'case', 'Mesenchyme', 'transplant', 'French', 'matters needing attention', 'Refractory', 'cure', 'patient', 'health' | Can systemic lupus erythematosus stem cells be treated |
| 13 | 465 | 1 | vaccine', 'New crown', 'Make a new crown', 'inoculation', 'Kexing', 'Systematic', 'hpv', 'Yes no', 'No', 'sister', 'Yes', 'reply', 'doctor', 'Lupus erythematosus', 'excuse me', 'Nine valence', 'Make nine price', 'Like rain', 'forty-nine thousand nine hundred and fifty-six', 'today' | It seems that I really can't get the new crown vaccine |
| 14 | 452 | 4 | joint', 'Arthritis', 'desiccation', 'rheumatism', 'Rheumatoid', 'pain', 'rheumatism', 'gout', 'connective tissue', 'factor', 'Myalgia', 'Rheumatic arthritis', 'gout', 'fibre', 'symptom', 'disease', 'diagnosis', 'Osteoarthritis', 'appear', '' | During the diagnosis of rheumatoid arthritis, it should be distinguished from arthritis caused by osteoarthritis, gouty arthritis, reactive arthritis, psoriatic arthritis and other connective tissue diseases (systemic lupus erythematosus, Sjogren's syndrome, scleroderma, etc.). (1) Osteoarthritis: this disease is a degenerative osteoarthropathy. The age of onset is mostly over 40 years old, mainly involving knee, spine and other weight-bearing joints. Osteoarthritis of the fingers is often misdiagnosed as rheumatoid arthritis, especially when the distal interphalangeal joint has Heberden nodules and the proximal interphalangeal joint has Bouchard nodules, it is easy to be regarded as synovitis. Most patients had normal ESR, negative rheumatoid factor or positive low titer. X-ray showed that the joint space was narrow, and the joint edge showed lip like hyperplasia or osteophyte formation. (2) Gout: chronic gouty arthritis is sometimes similar to rheumatoid arthritis. Gouty arthritis is more common in middle-aged and elderly men, and often occurs repeatedly. The predilection site is the unilateral first metatarsophalangeal joint, which can also invade the knee, ankle, elbow, wrist and hand joints. The blood uric acid level is usually increased in acute attack, and gout stones can appear in joints, auricles and other parts of chronic gouty arthritis. (3) . (4) Ankylosing spondylitis: this disease mainly invades the spine, but the surrounding joints can also be involved, especially those with knee, ankle and hip joints as the first symptoms, which should be differentiated from rheumatoid arthritis. The disease has the following characteristics: ① it is more common in young men; ② It mainly invades the sacroiliac joint and spine, and the peripheral joints are mainly involved in the asymmetric joints of the lower limbs, often with tendinitis; ③ 90% - 95% of patients were HLA-B27 positive; ④ Rheumatoid factor negative; ⑤ X-ray changes of sacroiliac joint and spine are very helpful for diagnosis. (5) Arthritis caused by connective tissue disease: Sjogren's syndrome and systemic lupus erythematosus can have joint symptoms, and some patients are positive for rheumatoid factor, but they all have corresponding characteristic clinical manifestations and autoantibodies. (6) . |
| 15 | 449 | 2 | Zhou Haimei', 'death', 'Refute a rumor', 'Dilemma', 'Living alone', 'sudden death', 'Death', 'heart disease', 'beauty', 'Lupus erythematosus', 'Burst', 'fifty-seven', 'Response', 'during his lifetime', 'online rumors', 'studio', 'performer', 'news', 'leakage', 'sister' | It is said that she has lupus erythematosus. Don't frighten me |
| 16 | 447 | 1 | Shuyang', 'Lu Zhi', 'full text', 'Really', 'Zhiyan', 'case', 'diary', 'That's all', 'Weekly hour', 'Warm metaphor', 'Look', 'Send a', 'break up', 'case', 'open', 'At that moment', 'thing', 'public', 'No. jade Mang', 'take out' | When Lu Zhiyan opened the next page, he found a case on the back. He opened the case with trembling hands, and the words on it stabbed into his heart. |
| 17 | 425 | 0 | Boy', 'Climb a building', 'Wanceng', 'ten', 'motion', 'come on', 'mislead', 'vigorous exercise', 'Inspirational', 'Lupus erythematosus', 'Vary from person to person', 'No', 'knee', 'insist on', 'Young man', 'Great', 'such', 'report', 'suit', 'No' | very good |
| 18 | 416 | 1 | sle', 'Popular science', 'Systematic', 'Grocery store', 'allay sorrow', 'medicine', 'medical treatment', 'stand above', 'Lupus erythematosus', 'Prevalence', 'Consulting room', 'lupus', 'Patients', 'second', 'world', 'our country', 'among', 'click', 'one hundred', 'Immunity' | SLE systemic lupus erythematosus |
| 19 | 401 | 0 | alopecia', 'hair', 'alopecia areata', 'hair follicle', 'scalp', 'hair', 'Hair grafting', 'androgen', 'fall off', 'scar', 'Rest', 'cause', 'grow', 'reason', 'Alopecia', 'Folliculitis', 'factor', 'sparse', 'Malnutrition', 'Seborrheic' | Is hair loss a disease. Androgenic alopecia, alopecia areata and telogen alopecia are the most common forms of hair loss. Androgenic alopecia, alopecia areata, postpartum alopecia, trichotillomania, traction alopecia, infectious alopecia, drug-induced alopecia, nutritional alopecia, and so on. There are many types of hair loss, among which the top three are androgenic alopecia, alopecia areata, and telogen alopecia. Androgenetic alopecia androgenetic alopecia is mostly seen in young and middle-aged men. At the initial stage, the hair on both sides of the forehead is thin and sparse, gradually extending to the top of the head, the hairline on the forehead retreats backward, and the forehead becomes high and M-shaped. With the gradual development of hair loss, the frontal and overhead hair loss can fuse with each other. In severe cases, only the occipital and bilateral temporal hair remains, showing a "Mediterranean" appearance. Most patients' hair is greasy, with a large amount of dandruff, generally no symptoms or itching, and some hair is dry and lacks luster. The speed and scope of hair loss vary from person to person, and most progress is slow. Alopecia areata, commonly known as "ghost shaving", can occur at any age, but it is more common in young adults, and there is no obvious difference between men and women. It shows that the hair loss area with round or oval shape and clear boundary suddenly appears on the head. The skin in the hair loss area is smooth, without inflammation, scales and scars. The "exclamation point" like hair is often seen on the edge, and it is easy to fall off with a slight pull. Alopecia areata can mostly recover. The newly grown hair is thin, soft, yellowish white, and gradually becomes thick and black until it returns to normal. Telogen alopecia hair loss caused by anxiety, tension, staying up late and other factors belongs to telogen alopecia, which can be recovered by changing the work and rest rules and abandoning bad living habits. How to distinguish androgenic alopecia from telogen alopecia? Professor yangxichuan told Fenghuang health, "the parts of hair loss are different. Baldness is obvious on the top of the head, and the hair line moves back obviously, while the hair loss caused by staying up late is diffuse hair loss, and there will be no obvious parts of hair loss." in addition, Professor yangxichuan believes that most bad habits in daily life have little impact on hair loss. But staying up late, irregular life, malnutrition, hyperthyroidism and other diseases will lead to telogen hair loss. "The use of shampoo with poor quality will not cause hair loss, but will at most cause dry hair, which is easy to break; while excessive cleaning will cause the impact of hair shaft, which affects the quality of hair. It belongs to hair breaking rather than hair loss, and the hair has not fallen out of the hair follicle." folk folk remedies such as wiping ginger to promote hair growth are not reliable. Professor Yang told Fenghuang health, "at present, the most effective topical hair growth drug is minoxidil. But the hair growth shampoo promoted in the market has no clear hair growth effect, which is suspected of false advertising in a sense." as for whether acupoint massage will be helpful for hair growth? Professor Yang said that in theory, scalp massage can improve the blood circulation of the scalp, but there is no sufficient evidence that acupoint massage can promote hair growth. Finally, Professor Yang reminded everyone that the severity of hair loss has an impact on disease diagnosis. For example, systemic lupus erythematosus has lupus hair, which is clinically characterized by more uneven broken hair and withered and yellow hair, which are even valuable for disease diagnosis. Like the lack of nutrition, hair will be worse. In short, the cause of hair loss should be clarified first. Some hair loss can be recovered by removing the inducing factors without any treatment. Therefore, we should first make clear whether the hair is decreasing. If it is determined that there is a problem, the cause should be found in combination with the medical history. The most common examination is to do hair pulling experiments. Pull about 20 hairs by hand, and the average drop after pulling is no more than 3. Even if it is normal, this experiment is for alopecia areata, which is of little significance for other types of hair loss, but it can also be done. |
| 20 | 373 | 1 | Hit hard', 'every time', 'recrudescence', 'Good health', 'dreadful', 'terror', 'hope', 'Lupus erythematosus', 'everybody', 'Frightening', 'safe and peaceful', 'Really', 'health', 'immune system', 'Once', 'well', 'Radical cure', 'High school biology', 'safety', 'Look' | Shit, what's this? Shit!!! |
| 21 | 357 | 3 | operation', 'myopia', 'cornea', 'cone', 'Eye', 'laser', 'degree', 'glaucoma', 'cataract', 'suit', 'femtosecond ', 'Myopia', 'serious', 'Contraindications', 'icl', 'vision', 'Postoperative', 'crystal', 'correct', 'severe' | Why are those ophthalmologists myopic but not do myopic surgery? Corneal laser myopia surgery has strict requirements on the thickness of the cornea, so "keratoconus" can not meet the standard of corneal laser myopia surgery! In addition, the following groups are not included in the scope of myopia surgery: minors and older people over 50 years old whose myopia has been rapidly deepening in the past two years. Those with eye diseases, such as active inflammation, glaucoma, keratoconus, cataract, severe amblyopia, etc. Those with depression and other psychological diseases. Uncontrolled autoimmune diseases, such as systemic lupus erythematosus, rheumatoid arthritis, etc. |
| 22 | 351 | 3 | Flying', 'Jiggle ', 'First time', 'Ruffian', 'close', 'contact', 'novel', 'remember', 'know', 'impression', 'notice', 'remember', 'Cai and Jiggle', 'empress', 'Heroine', 'In the book', 'butterfly', 'hear', 'Webwriting' | The first time I knew about this disease was because of the song "First Intimate Contact" by Qingwu Feiyang and Pizi Cai. |
| 23 | 343 | 1 | Drug resistance', 'artemisinin', 'team', 'break through', 'difficult problem', 'obtain', 'Antimalarial', 'scientific research', 'aspect', 'Overcome difficulties', 'New progress', 'reply', 'mechanism', 'propose', 'origin', 'works', 'practical', 'World Health Organization', 'Authoritative expert', 'region' | In view of the problem of "drug resistance" of artemisinin in some parts of the world in recent years, Tu you you and her team have made new breakthroughs in "Research on the mechanism of malaria resistance", "causes of drug resistance" and "adjustment of treatment methods" after years of hard work. Recently, they proposed a practical treatment plan to deal with the problem of "artemisinin resistance", and made new progress in "indications of artemisinin in the treatment of lupus erythematosus" and "traditional Chinese medicine scientific research works going out", which were highly recognized by the World Health Organization and authoritative experts at home and abroad. |
| 24 | 333 | 1 | outpatient service', 'Disease species', 'medical insurance', 'chronic disease', 'Insured', 'medical insurance', 'special', 'personnel', 'Reimbursement', 'payment', 'treatment', 'medical treatment', 'salvation', 'limit', 'basic', 'Chronic idiopathic disease', 'guarantee', 'appraisal', 'fixed point', 'declare' | Baoding Qingyuan District Medical Security Bureau medical insurance outpatient special disease policy understanding paper 1. Outpatient chronic disease and outpatient serious disease disease disease scope outpatient chronic disease disease disease scope: chronic obstructive pulmonary emphysema, chronic pulmonary heart disease, nervous system disease (cerebrovascular disease sequelae serious functional disorder), circulatory system disease (angina pectoris, myocardial infarction, ischemic cardiomyopathy, myocardial fibrosis, chronic heart failure, rheumatic heart disease), chronic hepatitis active stage (chronic moderate to severe viral hepatitis), liver cirrhosis, diabetes mellitus (with serious complications), peptic system ulcer (gastric ulcer, duodenal ulcer), hypertension (with serious complications), immune system disease (systemic lupus erythematosus, rheumatoid arthritis serious limb dysfunction), Sjogren's syndrome, rheumatoid arthritis, chronic hepatitis B, chronic hepatitis B, chronic hepatitis B, chronic hepatitis C, chronic hepatitis B, chronic hepatitis C, chronic hepatitis B, chronic hepatitis C, chronic hepatitis C, chronic hepatitis C, chronic Mental system diseases (schizophrenia, depression, obsessive-compulsive disorder, paranoid psychosis, bipolar disorder, mental disorder caused by epilepsy), urinary system diseases (nephrotic syndrome, chronic renal failure, chronic nephritis), inflammatory bowel disease (ulcerative colitis, Crohn's disease), Parkinson's disease, femoral head necrosis, pituitary tumor, primary pulmonary fibrosis, arteriosclerosis obliterans, myasthenia gravis, epilepsy, active tuberculosis, cerebral palsy. |
| 25 | 316 | 0 | immunity', 'Yang qi', 'forming', 'human body', 'intestinal tract', 'immune system', 'immune', 'cell', 'Is', 'gasification', 'function', 'probiotics', 'Traditional Chinese Medicine', 'Western medicine', 'organism', 'Intestinal leak', 'Yang deficiency', 'Flora', 'material', 'body' | There is such a view in the Yellow Emperor's Canon of internal medicine: "Yang turns into Qi, while Yin forms". Zhangjingyue, a famous doctor in the Ming Dynasty, believed that "Yang moves and disperses, so it turns into Qi, while Yin is still and coagulates, so it takes shape." |
| 26 | 315 | 0 | stem cells', 'Mesenchyme', 'cell', 'Umbilical cord blood', 'transplant', 'differentiation', 'repair', 'umbilical cord', 'hematopoiesis', 'have', 'research', 'organization', 'treat', 'application', 'clinical', 'regenerate', 'disease', 'effect', 'Allogeneic' | Which hospital can do stem cells to enhance immunity? Stem cells can be used to enhance immunity in domestic stem cell clinical research filing institutions or hospitals approved by the state. Immunity is an important line of defense against disease. When human immunity declines, it is vulnerable to various diseases. Stem cell therapy injects healthy stem cells into patients, which can stimulate the body to produce more immune cells, thereby improving immunity. At the same time, stem cells can also repair damaged immune cells and restore the normal function of the immune system. . |
| 27 | 314 | 1 | artemisinin', 'Effective rate', 'team', 'unique', 'nine thousand and eighty', 'Dihydrogen', 'test', 'Xinshuang', 'tablet', 'prophase', 'Fastest', 'expect', 'part', 'two thousand and twenty-six', 'smoothly', 'observation', 'Variability', 'effect', 'around' | According to the preliminary clinical observation of Tu youyou's team, the effective rates of artemisinin in the treatment of discoid lupus erythematosus and systemic lupus erythematosus were more than 90% and 80% respectively. . |
| 28 | 312 | 2 | High incidence', 'young', 'crowd', 'woman', 'childbearing age', 'Good health', 'Lupus erythematosus', 'safe and peaceful', 'everybody', 'Retreat retreat', 'hope', 'dreadful', 'well', 'Frightening', 'safety', 'frighten to death', 'easily', 'take care', 'understand', 'Love' | What is it exactly? |
| 29 | 294 | 0 | immunity', 'Two characters', 'work', 'Neither enemy nor foe', 'balance', 'equation', 'strong', 'immune system', 'health products', 'disseminate', 'Get sick', 'Allergic', 'Nutrients', 'nutrition', 'Rheumatoid', 'Stronger', 'self', 'Higher, higher', 'normal', 'Immunity' | Seven favorite nutrients of the immune system! "Health = less illness = strong immunity", such an "equation" is often used in the promotion of health products. If the immune response is too strong, it is easy to "distinguish between ourselves and the enemy", leading to autoimmune diseases or allergic diseases such as lupus erythematosus, rheumatoid, etc. Immunity needs 7 kinds of nutrition most ↓ |
| 30 | 275 | 3 | fifteen', 'licorice', 'cinnamon twig', 'nn', 'ten', '30g', 'Our party', 'Indications', 'Aconite', 'twenty', 'Pinellia ternata', '15g', 'Rehmannia glutinosa', '10g', 'thirty', 'Bupleurum chinense', 'Poria cocos', 'Anemarrhena asphodeloides', 'prescription', 'ginseng' | Indications of classical prescriptions based on syndrome differentiation (practical version) 1. Lizhong decoction Representative prescription of deficiency cold disease of digestive system It can be seen from the hypersecretion of digestive juice and its low function. The evidence shows that the upper part is salivation, or salivation, (children) or oral ulcer, the middle part is epigastric pain, gas swallowing, and the lower part is diarrhea.2. Dahuang Mudan decoction Lower abdomen infection prescription Prostatitis, etc.3. walnut Chengqi Decoction |
| 31 | 274 | 0 | Sunscreen', 'laser', 'skin', 'photon', 'sunlight', 'lattice', 'ultraviolet radiation', 'Rejuvenation', 'photosensitive', 'Sun exposure', 'Depilation', 'Stain', 'scar', 'sunshine', 'Sex dermatitis', 'light', 'laser therapy ', 'sensitive', 'Sunscreen', 'suit' | Nowadays, people's awareness of sunscreen is getting stronger and stronger. Many people know the importance of sunscreen cosmetics. They don't go out without sunscreen. But many people still don't pay attention to sunscreen when they go out, and you know what? If you don't pay attention to sunscreen in summer, be careful of sunburn dermatitis when you go out, so you should do well in daily sunscreen. |
| 32 | 258 | 3 | No.2', 'sound made by a hen', 'Jiajia', 'Three', 'Fortune and longevity', 'auspicious', 'Living alone', 'Medical treatment', 'vicious', '24h', 'Can be cured', 'Bluff', 'Sledgehammer', 'Overbearing', 'fall in love with', 'Too weak', 'Burst', 'marry', 'look after', 'Someone' | Can she cure lupus erythematosus by getting married? |
| 33 | 243 | 4 | Ankylosis', 'Rheumatoid', 'Arthritis', 'Spondylitis', 'rheumatism', 'rheumatism', 'gout', 'consult', 'joint', 'Love medicine day', 'desiccation', 'Rheumatic arthritis', 'Osteoarthritis', 'free', 'Immune disease', 'Quota', 'Behcet's disease', 'Scleroderma', 'Syndrome', '' | The difference between rheumatism and rheumatoid is not a concept. Rheumatism is a large group of diseases with connective tissue damage, mainly arthritis, skin and muscle damage, including rheumatoid arthritis, Sjogren's syndrome, etc; Rheumatoid arthritis is a kind of rheumatic disease, which is characterized by symmetrical swelling and pain of multiple joints, especially small joints. Rheumatoid arthritis can also have lung, kidney and other organ damage. Rheumatism covers a wide range, including not only rheumatoid arthritis, but also osteoarthritis and lupus erythematosus. |
| 34 | 228 | 5 | decision', 'Draw lots', 'daughter', 'parent', 'Nanchang', 'Jiangxi', 'raise', 'Stay', 'wife and daughter', 'world', 'Couple', 'grow up', 'Go with you', 'Physical illness', 'Rest of life', 'give up', 'Helpless', 'Very long', 'Find out' | On June 26, the parents in Nanchang, Jiangxi Province chose to draw lots to decide who would stay in the world to raise their daughter and grow up with her. This helpless decision was made because her daughter was recently found to have "lupus erythematosus", and both husband and wife were suffering from cancer. The husband decided to give up treatment and use all the money to save his wife and daughter. |
| 35 | 218 | 5 | Painful', 'Psychosis', 'thirteen', 'girl', 'Get well soon', 'poor', 'hope', 'pain', 'Lupus erythematosus', 'Little girl', 'come on', 'Distressed', 'Girls', 'children', 'Sickness and pain', 'Little girl', 'Radical cure', 'Misdiagnosis', 'fourteen', 'Nursing' | I also have lupus erythematosus, but it's so serious |
| 36 | 210 | 1 | chloroquine', 'phosphoric acid', 'sulphuric acid', 'New crown', 'pneumonia', 'malaria', 'Trump', 'Archie', 'virus', 'Mycin', 'medicine', 'For', 'coronavirus', 'take', 'Yellow Sea', 'treat', 'hydroxyl', 'effect', 'new type', 'apply' | Can the lupus drug "chloroquine" turn around again to treat covid-19? Chloroquine and hydroxychloroquine belong to quinolone anti infective drugs. They are a class of synthetic antibiotics. In addition to chloroquine drugs, moxifloxacin and ciprofloxacin are common. Chloroquine is an ancient drug, which initially shone brilliantly in history as an antimalarial drug. Its second bloom in the medical field is due to its prominent immune regulatory effect. As a common anti rheumatic drug, it is mainly used to treat rheumatoid arthritis, discoid lupus erythematosus and systemic lupus erythematosus. Chloroquine can resist light sensitivity and protect cartilage mainly by inhibiting interleukin-1 and reducing self antigen reaction. Chloroquine takes effect slowly by oral administration. Generally, the curative effect can reach the peak after 3-4 months of taking medicine. The dosage is 250mg per day, taken twice. Chloroquine has few adverse reactions, but it may cause blindness due to retinal degeneration. Fundus examination needs to be done every six months. In addition, it may cause arrhythmia, and ECG needs to be reviewed regularly. Chloroquine and hydroxychloroquine are related drugs with similar clinical indications and similar retinal toxicity manifestations, although their therapeutic and toxic doses are different. Hydroxychloroquine is also known as hydroxychloroquine sulfate, hydroxychloroquinoline sulfate, hydroxychloroquine sulfate and hydroxychloroquine sulfate. To sum up, chloroquine ≈ hydroxychloroquine = hydroxychloroquine sulfate. |
| 37 | 199 | 0 | oral ulcer', 'early stage', 'frighten to death', 'ulcer', 'Thu thu', 'Shiver', 'Watermelon frost', 'Don't be frightening', 'in the mouth', 'Frightening', 'Lupus erythematosus', 'manufacture', 'anxious', 'random', 'Entry', 'panic', 'Inflamed', 'vocal', 'each', 'once' | In oral ulcer |
| 38 | 198 | 0 | Men', 'woman', 'Britain and America', 'cystitis', 'Male and female sex', 'Much higher than', 'Heavy female', 'Osteoporosis', 'hypothyroidism ', 'mammary cancer', 'Common', 'remind', 'Incidence', 'Prevention', 'depression', 'masculine', 'hyperthyroidism', 'On the body', 'public', 'more and more' | Some diseases are obviously "more female than male", and the incidence of female is much higher than that of male. 1. osteoporosis. 2. breast cancer. 3. hyperthyroidism and hypothyroidism. 4. eating disorder. 5. cystitis. 6. depression. 7. lupus erythematosus. |
| 39 | 198 | 1 | Approved', 'Monoclonal antibody', 'ratify', 'Bailey', 'First', 'Biological agents', 'glaxo ', 'list', 'Shike', 'For', 'Global', 'benlysta', 'Clinical trials', 'gsk', 'State Drug Administration', 'Hengrui pharmaceutical', 'China', 'Indications', 'new drug', 'Mobility' | Belizumab was approved by the State Food and drug administration to be listed for the treatment of systemic lupus erythematosus - the first new drug for systemic lupus erythematosus in 60 years was approved! Recently, GlaxoSmithKline (GSK) (belimumab) was approved by the State Drug Administration. Belimumab is used to treat systemic lupus erythematosus (SLE), which is another new drug for the treatment of SLE in China in the past 60 years. |
| 40 | 198 | 5 | twenty-six', 'last year', 'girl', 'Mom', 'children', 'Spawn', 'bearing', 'inheritance', 'Entry', 'Lupus erythematosus', 'respect', 'Vicious', 'oneself', 'Sick', 'No', 'family', 'disseminate', 'Eulogize', 'still', 'scourge' | A 26 years old girl with lupus erythematosus for five years became a mother last year |
| 41 | 188 | 3 | vicious', 'Young Master', 'crazy', 'Flower arrangement', 'Bluff', 'Fortune and longevity', 'Too weak', 'auspicious', 'Medical treatment', '24h', 'No.2', 'sound made by a hen', 'master', 'Living alone', 'Don't say', 'Can be cured', 'Burst', 'Jiajia', 'tiger', 'Three' | ? can someone take care of sudden diseases and send them to hospital within 24h without living alone |
| 42 | 182 | 4 | classroom', 'and', 'Open class', 'Rheumatology ', 'micro-blog', 'desiccation', 'Syndrome', 'health', 'doctor', 'Postgraduate entrance examination', 'Systematic', 'internal medicine', 'review', 'Punch in', 'surgery', 'English words', 'word', 'True topic', 'fracture', 'Lupus erythematosus' | Is Sjogren's syndrome common? |
| 43 | 173 | 0 | Oral ulcer', 'oral cavity', 'ulcer', 'Gingiva', 'mucous membrane', 'Behcet ', 'Oral cancer', 'plant', 'Behcet's disease', 'teeth', 'vitamin', 'Oral mucosa', 'canceration', 'Brushing teeth', 'repeatedly', 'Recurrent', 'accompanied by', 'lesion', 'hemorrhage' | Many people think that an ulcer in the mouth is due to a lack of vitamins or trace elements. However, this is the case with very few oral ulcers. Usually, local trauma, mental stress, food, drugs, malnutrition, hormone level changes, etc. may induce oral ulcers, and blind large-dose vitamin supplementation is easy to cause other health problems. Moreover, some fruits are high in sugar, so eating too much or not paying attention to cleaning after eating is not conducive to oral health. Some oral ulcers may indicate that the body has potential systemic diseases, such as oral ulcers and gastric ulcers, duodenal ulcers, ulcerative colitis, localized enteritis, vitamin B absorption disorders, etc., which are all associated with oral ulcers. For example, 30% - 48% of patients with oral ulcers have digestive tract diseases, accompanied by abdominal distension, diarrhea or constipation. Ordinary oral ulcer is a self-healing disease. Even if it is not treated, it will recover in 5-10 days. However, if oral ulcer is accompanied by repeated attacks, long-term treatment, large ulcer surface, surrounding bulge, abnormal pain, mass at the gingiva, general fatigue, obvious short-term weight loss, cervical lymph node enlargement and other conditions, we should be alert enough. Because many systemic diseases will manifest as local oral mucosal ulcers, such as systemic lupus erythematosus, Behcet's disease, rheumatism and so on, which often appear repeated oral ulcers, and the oral ulcers that have not been cured for more than two weeks are also at risk of canceration. During the onset of oral ulcer, we can reduce the degree of pain and shorten the healing time: eat more zinc containing food for daily maintenance to promote wound healing, such as lean meat, eggs, walnuts, peanuts, etc; Drink boiled water as much as possible; Avoid smoking and drinking alcohol, coffee and other stimulating beverages. |
| 44 | 166 | 5 | bearing', 'Pregnancy', 'normal', 'West China', 'children', 'Custody ', 'No', 'inheritance', 'Can produce', 'Lupus erythematosus', 'statute', 'contraception', 'Zhangsilai', 'influence', 'Extremely high', 'would', 'article', 'reply', 'gestation', 'woman' | Can I have children normally if I have lupus? |
| 45 | 163 | 1 | Rongchang', 'SIPP', 'Tata', 'biology', 'new drug', 'innovate', 'Yantai', 'list', 'research and development', 'Billion yuan', 'Global', 'adc', 'rc18', 'company', 'Biopharmaceuticals', 'Indications', 'enterprise', 'First', 'clinical' | On April 11, Taiai (common name: taitacep), an innovative new biological drug for the treatment of systemic lupus erythematosus (SLE) independently developed by Rongchang biopharmaceuticals (Yantai) Co., Ltd. (hereinafter referred to as Rongchang biopharmaceuticals), held a listing meeting in Beijing. Lupus erythematosus is a typical autoimmune connective tissue disease. SLE is the most common type of lupus erythematosus and the most serious type of lupus erythematosus. It mostly occurs in women aged 15-45 years old. At present, the number of SLE patients in China exceeds 1million. At the meeting, Professor Zeng Xiaofeng, director of Rheumatology and Immunology Department of Peking Union Medical College Hospital, said: "SLE treatment has always been the heart knot of rheumatic and immune disease doctors, especially in the treatment of SLE, there is a lack of doctors and drugs, which has been significantly improved in recent years, but the lack of drugs still exists. In China, there are still serious problems in the treatment and prognosis of SLE, and new drugs are needed. ”"Many factors are involved in the pathogenesis of SLE, among which B cells are the core of the pathogenesis of SLE, BLyS and April are the key factors for the differentiation and maturation of B cells. Etanercept is a blys/april dual target inhibitor for the treatment of SLE. ”Professor limengtao, chief physician of Rheumatology and Immunology Department of Peking Union Medical College Hospital, introduced at the meeting that the phase IIB clinical study of etanercept in the treatment of SLE showed that patients in the tai'ai treatment group (80mg, 160mg, 240mg) had a high response rate of sri-4 at 48 weeks. The response rate of the tai'ai high-dose group was 79%, significantly higher than 32% of the conventional treatment group. Compared with existing drugs, the curative effect of tai'ai high-dose group was more significant, with higher biological activity and safety, which brought hope for preventing disease recurrence and reducing adverse drug reactions. ”Professor Zhangfengchun, chief physician of Rheumatology and Immunology Department of Peking Union Medical College Hospital, said. On March 12, the State Food and Drug Administration approved the listing of etanercept for injection, a therapeutic biological product declared by Rongchang biology, conditionally through the priority review and approval procedure. At present, patients can buy etanercept through doctors' prescriptions. At present, Rongchang biology has built a top-level marketing team in the industry and is fully prepared for commercialization. In addition, Rongchang biology is also actively promoting the globalization of techpool. It is reported that the product has been approved by the FDA to carry out a US registered clinical trial of etanercept for the treatment of SLE. In April 2020, the FDA granted the fast track qualification of etanercept; In September 2020, it was approved by the US phase II clinical trial (ind); . It is expected that etanercept has not only achieved an important breakthrough in the treatment of systemic lupus erythematosus, but its domestic phase II / III clinical trials for the treatment of IgA nephritis, Sjogren's syndrome, neuromyelitis optica, multiple sclerosis, myasthenia gravis and other indications have been fully launched, and a global multi center clinical study will be launched for several indications. |
| 46 | 154 | 1 | Kunyao', 'group', 'Clinical trials', 'artemisinin', 'Dihydrogen', 'Class products', 'corporate performance ', 'be responsible for', 'company', 'tablet', 'Enrollment', 'research and development', 'Daily limit', 'unit', 'purpose', 'develop', 'announcement', 'sales', 'at present' | According to the approval document for clinical trials of drugs issued by the State Drug Administration, the application for "clinical trial of dihydroartemisinin tablets for the treatment of systemic lupus erythematosus and discoid systemic lupus erythematosus" submitted by the Institute of traditional Chinese medicine of the Chinese Academy of traditional Chinese medicine where Tu youyou's team is located has been approved. Kunming Pharmaceutical Group Co., Ltd. carried out clinical trials as the responsible unit. The reporter learned that clinical trials generally have three phases. The sample size of phase II and phase III trials is larger, and it will take at least 7 to 8 years. If the test is successful, it is expected that the new dihydroartemisinin tablets will be approved to market as soon as around 2026. |
| 47 | 154 | 3 | traditional Chinese medicine', 'Dry itch', 'Oneortwo days', 'Xerosis', 'Conception rate', 'recently', 'Dry', 'climacteric', 'acupuncture', 'point out', 'Moisture', 'Soon', 'comfortable', 'Nourishing Yin', 'Traditional Chinese Medicine', 'Infertility', 'fever', 'available', 'recovery', 'common cold' | 1. Cold and fever. The effect of traditional Chinese medicine is very fast. Usually it can improve uncomfortable symptoms in a day or two. 2. Infertility. Recent studies have pointed out that acupuncture and traditional Chinese medicine can improve the pregnancy rate. 3. Xerosis. . |
| 48 | 152 | 2 | tourism', 'High fever', 'Immortality', 'woman', 'cancer', 'title', 'diagnose', 'Bask in the sun', 'Sunscreen', 'ultraviolet radiation', 'Lupus erythematosus', 'induce', 'dreadful', 'environment', 'Sun exposure', 'Manual labor', 'go out', 'come in', 'Insolation', 'indeed' | Is it lupus erythematosus after a little sun exposure?! |
| 49 | 147 | 0 | Butterfly shape', 'viscera', 'Involvement', 'system', 'cheek', 'erythema', 'appear', 'Readily available', 'susceptibility', 'typical', 'self', 'Heavyweight', 'bite', 'Pathogenicity', 'Swelling and pain', 'endanger', 'May cause', 'Immunity' | Systemic lupus erythematosus is an autoimmune disease that invades multiple systems. In mild cases, it only affects the skin and causes erythema, while in severe cases, it affects multiple organs and is life-threatening.This disease can cause a large number of pathogenic autoantibodies and immune complexes in the body, causing damage to multiple systems and organs of the whole body. Due to hormone effects, differences in immune function, genetic susceptibility and other reasons, women are more likely to get it, especially the "butterfly rash" on the cheek and nasal bridge. Fatigue, joint swelling and pain may also occur. |
| 50 | 143 | 2 | Zhou Haimei', 'Death', 'Zhouzhiruo', 'Death', 'Hong Kong', 'twelve', 'fifty-seven', 'news', 'Beijing', 'Lvliangwei', 'studio', 'Netizen', 'Breaking the news', 'nn', 'be backed up by Heaven', 'Dragon slaying', 'Seafood', 'platelet', 'role', 'medical record' | Zhou Haimei clarified that she had thrombocytopenia in an interview with traditional Chinese medicine magazine. Zhou Haimei said that she developed bruises as long as she had a slight collision, and she was not unhappy when she knew she was suffering from this disease. In addition to the doctor's medication, the traditional Chinese medicine also reminded her to stay relaxed. She said psychotherapy is more important. Optimism can cure any disease. |
| 51 | 140 | 3 | condyloma acuminatum', 'recrudescence', 'probability', 'sexual behaviour', 'Verrucous body', 'Multiply', 'renal transplant', 'Low', 'sexual life', 'Recurrence rate', 'morbidity', 'Sexual partners', 'hpv', 'Condyloma', 'Malignant tumor', 'genitals', 'infect', 'smoke', 'application', 'Youjia' | Under what circumstances is it easy to infect condyloma acuminatum when it comes to condyloma acuminatum, many people will be familiar with this disease. According to the survey, condyloma acuminatum is mainly transmitted through sexual behavior, and the incidence is high in people with chaotic sexual life. 1. . 2. Trauma infection with trauma if you come into contact with the secretions of patients with condyloma acuminatum or daily appliances contaminated by patients, warts bacteria will enter the human body through the wound, which will cause people to suffer from condyloma acuminatum. 3. Unclean sexual life is the main reason for infection with condyloma acuminatum. If sexual partners are not fixed in daily life and sexual life is more prevalent, it is very easy to suffer from condyloma acuminatum. |
| 52 | 140 | 0 | ointment', 'Cream', 'ointment', 'Corticosteroids', 'For', 'Hormones', 'Prednisone', 'dermatitis', 'effect', 'Sex dermatitis', 'Antipruritic', 'Antiallergic', 'hormone', 'dexamethasone', 'apply', 'hydrogenation', 'Glucocorticoid', 'apply', 'eczema' | There are many kinds of small ointments commonly used in dermatology. Here are 18 kinds of commonly used ointments. 1. Desonide ointment: it is mainly applicable to skin inflammation and skin itching caused by contact dermatitis, neurodermatitis, seborrheic dermatitis, eczema, psoriasis, lichen planus, simple lichen, blistering, etc. |
| 53 | 139 | 2 | Tianqiwen', 'Zhou Haimei', 'beset', 'entertainment', 'long-term', 'Billion points', 'exposure', 'Death', 'plan', 'sister', 'video', 'micro-blog', 'Get well soon', 'All the way', 'goddess', 'works', 'heaven', 'Lupus erythematosus', 'Lvliangwei', 'Sickness and pain' | I wish Zhou Haimei an early recovery. What is lupus erythematosus? |
| 54 | 133 | 2 | Immortality', 'cancer', 'Called', 'Lupus erythematosus', 'artist', 'plan', 'Radical cure', 'suffer', 'woman', 'Systematic', 'cannot', 'science', 'dreadful', 'Ankylosis', 'Spondylitis', 'deadly', 'Micro pressure', 'Many', 'such' | Systemic lupus erythematosus (SLE) is systemic lupus erythematosus (SLE). What kind of cancer can survive if it is discovered early |
| 55 | 132 | 5 | Chilblain', 'in one's hands', 'think', 'girl', 'diagnose', 'ninety-five', 'dreadful', 'Lupus erythematosus', 'Frightening', 'definite', 'Find out', 'Strongest', 'Defensive line', 'Good health', 'Oh, my God', 'young', 'Select', 'LiuQian', 'winter', 'importance' | I don't think so |
| 56 | 131 | 1 | Tacrolimus', 'capsule', 'Dressing change', 'A box', 'Two boxes', 'Reimbursement', 'cheap', 'Systematic', '05mg', 'Saifu', '1mg', 'Yes no', 'Cyclosporine', 'Private chat', 'East China', 'Provincial Department', 'Sanya', 'Lupus erythematosus', 'change into', 'review' | Is there anyone who resells tacrolimus? |
| 57 | 131 | 3 | operation', 'myopia', 'Not', 'vision', 'Fallback', 'everyone', 'femtosecond ', 'suitable', 'laser', 'formulate', 'ophthalmology', 'mature', 'taboo', 'All', 'remind', 'habit', 'detailed', 'reasonable', 'scar' | The doctor reminded: femtosecond laser surgery is very mature in China, but it is not suitable for everyone, and there are strict taboos. Patients with severe scar constitution, diabetes, lupus erythematosus and other diseases need to undergo a detailed ophthalmic examination to determine whether they are suitable for surgery and formulate a reasonable surgical plan. Avoid complications or vision regression. |
| 58 | 128 | 5 | test tube', 'lady', 'Mrs.', 'children', 'decision', 'twins', 'success', 'Huaiyun', 'tell', 'Pregnant', 'advanced age', 'mother', 'bearing', 'Give birth to', 'In vitro fertilization', 'sperm', 'want', 'Overjoyed' | Ms. Cang had always wanted to have children, but when she was diagnosed with systemic lupus erythematosus, she thought her dream of motherhood was over. However, her doctor told her about IVF, and Mrs. Cang decided to have a try. After months of treatment, Mrs. Cang finally conceived twins. She was overjoyed and couldn't wait to see her children. Unfortunately, Mrs. Cang's luck is not easy. Because of her condition, she had to stay in bed most of the time and was constantly monitored by her doctor. But she was determined to overcome the difficulties and eventually gave birth to two healthy children, a man and a woman. Although this is a difficult journey, Mrs. Cang is grateful that she can have children and establish her own small family. |
| 59 | 127 | 3 | Ten years', 'music', 'singer', 'boss', 'creation', 'sincere', 'Fans', 'Dream weaving sound', 'stage', 'Zhongzi', 'Keep a low profile', 'Admission ticket', 'publisher', 'Ever suffered from', 'Transparent', 'Lupus erythematosus', 'modest', 'grow up', 'Interview' | This is an immune system disease, which does not affect work and life. With a childlike heart, Yu Tian has gone through these ten years, from fighting with himself to reconciling with himself. What he has changed is his age and experience, and what remains unchanged is his attitude of still loving music?? In the next ten years, keep going with music |
| 60 | 122 | 4 | Syndrome', 'Los', 'Les', 'houson ', 'houson', 'Houxi', 'mast cells', 'position', 'Tachycardia', 'Live in', 'Endometriosis ', 'shock', 'activation', 'intima', 'many times', 'at that time', 'uterus', 'Get well soon', '' | Oh my god get well soon. In addition to suffering from lupus erythematosus, Houxi was also diagnosed with leukemia! She was really ill fated. She had been hospitalized with anaphylactic shock for many times after giving birth. At that time, she was diagnosed with endometriosis, Ehlers Danlos syndrome, Sjogren's syndrome, mast cell activation syndrome, postural tachycardia syndrome... Alas |
| 61 | 116 | 1 | Yes no', 'which', 'recommendation', 'Patients', 'hospital', 'Wuhan', 'excuse me', 'compare', 'once', 'Systematic', 'junior partner', 'Guangzhou', 'Zhongshan', 'First hospital', 'doctor', 'sister', 'Which one', 'Shanghai', 'Lupus erythematosus', 'everybody' | Are there any sisters who see traditional Chinese medicine in Wuhan? Are there any recommended doctors |
| 62 | 115 | 3 | Offline', 'Green sea', 'Wangyibo', 'Night of microblogging', 'Aquatic products', 'unearthed', 'Motorcycles', 'Fans', 'magazine', 'broom', 'scene', 'sales volume', 'Bastard', 'Light plate', 'Online', 'stage', 'potato', 'Ministerial level' | Since there are so many microblogging nights??? It won't be sold to other families by big fans. Where are all the offline people? Anyway, we have enjoyed a few green seas this year. |
| 63 | 113 | 3 | Dry eye', 'tear', 'Dry eye', 'Eyes', 'contact lenses', 'evaporation', 'blink', 'secretion', 'Dry and astringent', 'Eye drops', 'air-conditioning', 'Eye', 'Plate gland', 'computer', 'cornea', 'Ocular surface', 'apply', 'Wear' | Patients with certain diseases: some diseases will affect our immune system, make it out of balance and attack our own body. For example, Sjogren's syndrome, rheumatoid arthritis, diabetes, graft-versus-host disease and lupus erythematosus. These diseases may damage our lacrimal gland or other ocular tissues, resulting in insufficient or poor quality tear secretion, causing dry eye. |
| 64 | 112 | 1 | drug', 'catalog', 'medical insurance', 'Inclusion', 'country', 'Medication', 'new edition', 'negotiation', 'New', 'Enable', 'one hundred and nineteen', 'Price reduction', 'pneumonia', 'five thousand and sixty-four', 'Anticancer drugs', 'Latest edition', 'New crown', 'formal', 'average', 'price' | The catalogue of drugs for national basic medical insurance, work-related injury insurance and maternity insurance (2020) has been officially launched. It includes 119 new drugs, with an average price drop of 50.64%. Drugs benefit a wide range of treatment fields, including drugs for chronic obstructive pulmonary disease, lupus erythematosus, antidepressant drugs, etc. The new national medical insurance drug catalog added 17 new anticancer drugs, including PD-1, lenvatinib, etc. |
| 65 | 111 | 3 | 15g', 'fifteen', 'thirty', 'usage', 'everyday', 'Decocted in water', 'Paeonia lactiflora Pall', 'Cortex Moutan', 'Birthplace', 'twenty', 'licorice', '30g', 'form', '10g', 'Therapeutic method', 'ten', 'Forsythia suspensa', 'Secondary service', 'angelica', 'Smilax glabra' | Lupus erythematosus traditional Chinese medicine prescription (I) syndrome differentiation liver depression turns into heat, heart fire is blazing, blood heat turns into blood stasis. treatment clear heart fire, cool blood heat, and detoxify heat. Fang name xiaoxiaoling. composition 20g rehmannia root, 15g Paeonia lactiflora, 15g Cortex Moutan, 15g Achyranthes bidentata, 15g Sophora flavescens, 20g dandelion, 20g Viola yedoensis, 15g pollen, 15g angelica, 15g Forsythia suspensa, 15g Scutellaria baicalensis Georgi and 10g licorice. usage soak the medicine with proper amount of water for 30 minutes, and then put it on slow fire to decoct for 30 minutes. Each dose is decocted twice, and the two decocted solutions are mixed. Take 1 dose daily in the morning and evening. source Han bailing Fang |
| 66 | 110 | 0 | thyroid', 'Hashimoto', 'thyroiditis', 'hyperthyroidism', 'Goiter', 'function', 'tsh', 'hypothyroidism ', 'Thyroid cancer', 'jia', 'antibody', 'Immunity', 'self', 'Jiagong', 'tpoab', 'peroxide', 'disease', 'rise', 'tgab', 'thyroxine' | Studies have shown that people with Hashimoto's thyroiditis are more likely to develop other autoimmune diseases, such as rheumatoid arthritis and systemic lupus erythematosus?? Therefore, once Hashimoto's thyroiditis is found, attention must be paid to it. The earlier the intervention, the easier it will be to control. |
| 67 | 109 | 3 | Dunhuang', 'grape', 'one hundred thousand', 'hibernation', 'Jiggle ', 'tyre', 'Male master female', 'Dawei', 'lawyer', 'Mainly', 'tear', 'Lymphoid carcinoma', 'times', 'TV play', 'remember', 'forget', 'Part I', 'Instant noodles' | Isn't it lupus erythematosus |
| 68 | 108 | 0 | cart', 'therapy', 'cell', 'cd19', 'Targeting', 'treat', 'Chimerism', 'car', 'Antigen receptor', 'cure', 'reform', 'success', 'First case', 'research', 'immune', 'Hopefully', 'self', 'Germany', 'tumour', 'paper' | A health paper published in the British journal Nature Medicine showed that five patients with systemic lupus erythematosus had achieved drug free remission for 17 months after car-t cell therapy. Systemic lupus erythematosus (SLE) is an autoimmune rheumatic disease with an incidence of about 0.1% in the global population, which is highly prevalent in young women. Most patients are treated with glucocorticoids and therapies targeting T cells or antibody producing B cells. But these treatments are often ineffective, and there is no curative therapy at present. Car-t therapy refers to chimeric antigen receptor T cell immunotherapy, in which T lymphocytes, a kind of human leukocytes, are derived from bone marrow hematopoietic stem cells and can play immune functions in human blood, lymph and surrounding tissues and organs. Car is responsible for "positioning guidance" to help identify tumor cells in the body. Therefore, car-t therapy can be simply understood as a precision targeted therapy, which is fast and efficient and shows a very good prospect in clinical tumor treatment. This time, the research team of the University of Erlangen Nuremberg in Germany used the modified anti-CD19 chimeric antigen receptor (car) T cells to treat 5 patients with refractory SLE (4 females, 1 male, median age 22 years). These cells are designed to clear antibody producing B cells by targeting the CD19 protein produced on their surface. In the follow-up (3-17 months after treatment), it was found that all patients showed improvement of symptoms, including relief of internal organ burden and disappearance of disease-related autoantibodies, so traditional treatment was no longer necessary. Although these findings may suggest a new treatment option for SLE patients, it still needs to be followed up for a longer time in larger clinical trials to determine the safety and efficacy of car-t cell therapy in this situation. |
| 69 | 106 | 1 | compound', 'Feeding field', 'Deer chirp', 'Grass', 'A leaf', 'Nine figures', 'be known as', 'High efficiency', 'artemisinin', 'amazing', 'Antimalarial', 'Understand', 'Drug resistance', 'malaria', 'magical', 'Artemisia annua', 'Conquer', 'team', 'first choice' | "Yo Yo deer chirping, eating Artemisia annua", it is the magical "Chinese grass"; This compound called artemisinin is of great significance in fighting malaria and treating lupus erythematosus. Artemisinin is still the first choice of high-efficiency antimalarial drugs, but why the problem of artemisinin resistance?? Forward learning! |
| 70 | 104 | 1 | Bailey', 'Hit Bailey', 'Monoclonal antibody', 'Reimbursement', 'Systematic', 'sister', 'Yes no', 'medical insurance', 'review', 'seven hundred and fifty-five', 'excuse me', 'Needle Bailey', 'once', 'How's it going', 'Baileyou', 'Lupus erythematosus', 'January', 'Duplex', 'doctor' | The third dose of belivumab |
| 71 | 104 | 3 | Reynolds', 'nail', 'phenomenon', 'Cold hands and feet', 'finger', 'cold', 'pale', 'Reynolds ', 'colour', 'spasm', 'Scleroderma', 'Flushing', 'artery', 'blood vessel', 'appear', 'warm', 'emotion', 'Toes', 'Cold', 'Attacks' | A patient with systemic lupus erythematosus vs Raynaud's phenomenon asked, "is it because my condition has worsened that Raynaud's phenomenon occurs at the same time of suffering from systemic lupus erythematosus?" I believe that people who have the same experience have been terrified. |
| 72 | 103 | 4 | Home Online', 'Bone disease', 'rheumatism', 'academic research', 'rheumatism', 'diet', 'physiotherapy', 'recovered', 'introduce', 'patient', 'once', 'what', 'nursing', 'nephritis', 'treat', 'Lupus erythematosus', 'take care', 'food', 'early stage', 'Systematic' | Introduce what lupus erythematosus can eat |
| 73 | 99 | 2 | Vitiligo', 'pigment', 'Melanocytes', 'Henan', 'deletion ', 'Lanzhou', 'hospital', 'skin', 'Melanin', 'white', 'Melanin', 'patient', 'skin disease', 'part', 'spread', 'influence', 'cell', 'trace element', 'Chengdu' | When inflammation such as neurodermatitis, psoriasis or discoid lupus erythematosus subsides, local skin vitiligo may also appear, which is called secondary vitiligo. |
| 74 | 89 | 0 | Femoral head', 'necrosis', 'hormone', 'bone', 'substitution', 'hip joint', 'plaintiff', 'defendant', 'bilateral ', 'femur', 'Systematic', 'infarct', 'Osteomalacia', 'sister', 'Yes no', 'Open class', 'Lupus erythematosus', 'Osteonecrosis', 'micro-blog', 'cause' | Taking hormone to treat lupus erythematosus causes femoral head necrosis! |
| 75 | 88 | 0 | Have children', 'Going to the United States', 'single', 'baby', 'United States', 'Russia', 'uterus', 'Polycystic', 'Fallopian tube', 'Test tube baby', 'guest', 'congratulations', 'Mixed race', 'Older age', 'test tube', 'myoma', 'lovely', 'Adenomyosis', 'overseas', 'core' | Bask in the baby! The baby was born safely in the United States. The baby's mother was infertile due to blocked fallopian tubes. After going to the United States, the transplant was successful. Congratulations! |
| 76 | 87 | 0 | Meeting order', 'Chilly', 'decompression', 'damp', 'Work and rest', 'Life circle', 'away from', 'cctv', 'prevent', 'Sunscreen', 'appropriate', 'Do well', 'environment', 'See', 'avoid', 'health', 'video', 'diet', 'food', 'together' | ① Healthy work and rest, healthy diet, and proper decompression; ② Pay attention to sun protection; ③ ; ④ Stay away from foods that may irritate you. |
| 77 | 86 | 1 | inoculation', 'vaccine', 'New crown', 'virus', 'Live vaccines', 'Adenovirus', 'carrier', 'acute', 'Infected person', 'stable', 'proposal', 'crowd', 'recombination', 'control', 'Suspend', 'epidemic situation', 'good', 'Attacks', 'Postoperative' | Since the start of the new crown vaccination work, the hot lines of the masses have been continuously, "can I call in this situation?" has become one of the most concerned issues of the masses. Recently, the Municipal Center for Disease Control and Prevention (CDC) gave detailed and clear guidance and suggestions on what can and cannot be fought. |
| 78 | 84 | 1 | misunderstanding', 'common', 'good night', 'Retreat retreat', 'safe and peaceful', 'everybody', 'importance', 'Good health', 'Good health', 'Lupus erythematosus', 'important', 'definite', 'hope', 'turn', 'English', 'really', 'around', 'know', 'not', 'like' | Health is needed! |
| 79 | 80 | 3 | orange', 'Dream girl', 'travelers', 'Light rain', 'grandmother', 'At the beginning', 'Help', 'Nojiuzhou', 'pain', 'Look', 'Force a farewell', 'Wang Yuan', 'Spring is warm', 'Not smelling', 'No', 'come', 'Countdown', 'Kwai Ru' | It's an orange for help ... very painful |
| 80 | 78 | 4 | halsey', 'Houxi', 'leukemia', 'Western monkey disease', 'end', 'the', 'reveal', 'new song', 'oneself', 'issue', 'Get well soon', 'Alive', 'lucky', 'New album', 'singer', 'two years', 'share', 'My Haide', 'music', 'ig' | After suffering from lupus erythematosus and leukemia disappeared for so long, I was ill. I hope you can recover soon!! |
| 81 | 77 | 3 | eighty-three', 'wife', 'depression', 'For years', 'later period', 'father-in-law', 'grandma', 'biological father', 'look after', 'Father in law', 'Two', 'Problematic', 'mother-in-law', 'Fragrant clothes', 'Out of control', 'Maiyu', 'husband', 'Indecency', 'Lower body', 'cancer' | This man's mother has lupus, his father has suffered from depression for several years, and his father-in-law has terminal cancer. Less than three months after the operation, the wife has an 83 years old grandmother and two children to take care of. In this way, he could not control his lower body without delay. |
| 82 | 76 | 0 | Troubleshooting', 'face', 'erythema', 'Focus', 'cosmetology', 'once', 'skin', 'solve', 'Desquamation', 'symmetry', 'plaque', 'Sex dermatitis', 'cheek', 'Butterfly shape', 'symptom', 'diagnosis', 'distribution', 'contact', 'skin disease', 'Patch' | Symptoms of redness, swelling, dryness and desquamation of the cheek consider the symptoms of contact dermatitis caused by allergy to facial products. We should check autoimmune skin diseases. |
| 83 | 76 | 1 | SIPP', 'Tata', 'Yes no', 'Reimbursement', 'Out of Thailand', 'Patients', 'Systematic', 'Use Thai', 'excuse me', 'sister', 'Thai love', 'Tai AI', 'Yes', 'How's it going', 'effect', 'Someone', 'A stitch', 'Can turn' | Does anyone know about the drug "Taitasip"? |
| 84 | 74 | 0 | urticaria', 'allergy', 'histamine', 'chronic', 'ige', 'factor', 'etiology', 'Attacks', 'Wheal', 'food', 'physical', 'Cold and hot', 'pruritus', 'inducement', 'pollen', 'inhalation', 'Xinminkang', 'stimulate', 'infect' | ? Is urticaria always due to poor immunity? |
| 85 | 74 | 3 | tampons', 'assassin', 'charging', 'watch', 'Muffled sound', 'velvet', 'Mousse', 'December', 'Chengdu', 'cream', 'A smile', 'Bai Xiyan', 'inventory', 'dim sum', 'Bodhi', 'half', 'internal medicine', 'master', 'Ten', 'last year' | List of outpatient expenses of a patient with systemic lupus erythematosus last December |
| 86 | 73 | 1 | autonomously', 'research and development', 'Tert sodium', 'a batch', 'Alzheimer', 'State Food and Drug Administration', 'angina pectoris', 'help', 'numerous', 'new drug', 'Redeposit', 'Put into production', 'capsule', 'coronary heart disease', 'In recent years', 'ratify', 'innovate', 'recently', 'our country' | Recently, mannate sodium capsules were officially approved by the State Food and drug administration, which means that this treatment. In recent years, a number of innovative drugs independently developed by China have brought new hope to many patients. Stamp image ↓↓ transfer to save! |
| 87 | 71 | 1 | company', 'develop', 'US $100 million', 'market', 'innovate', 'field', 'biology', 'research and development', 'Pipeline', 'domestic', 'gsk', 'Monoclonal antibody', 'China', 'list', 'clinical', 'product', 'Global', 'increase', 'Billion yuan', 'Self exemption' | The field of autoimmune diseases is the second largest drug market in the world after tumor, with a huge market size and growth potential, which is attracting many pharmaceutical giants to actively expand their R & D pipelines. \N\n a few days ago, the two M & A transactions focused on the free zone on the same day. First, Johnson & Johnson, a giant in the field of immunization, purchased yellowjerseytherapeutics, a wholly-owned subsidiary of numab, for $1.25 billion to obtain the core pipeline il-4r/il-31 dual antibody of numab, which is being developed for the treatment of atopic dermatitis (AD). |
| 88 | 71 | 2 | High fever', 'Fuzhou', 'Fujian', 'Bask in the sun', 'Sanya', 'woman', 'Sea breeze', 'Emerge', 'a week', 'antigenicity', 'erythema', 'diagnose', 'Half', 'one's face', 'west', 'Pseudonym', 'decision-making' | Recently, in Fuzhou, Fujian Province, Xiaoyu (not her real name) spent a week in Sanya half a month ago, blowing the sea breeze and basking in the sun every day. After returning, the high fever didn't subside, the skin was sunburned, and some erythema appeared on the face. The patient was diagnosed with systemic lupus erythematosus. Doctors said that in a sunny environment, ultraviolet light will enhance the antigenicity and induce the occurrence of lupus erythematosus. After symptomatic treatment, Xiaoyu's high fever has subsided and her erythema has also subsided. |
| 89 | 67 | 3 | myopia', 'operation', '80%', 'Once and for all', 'Preoperative', 'High school students', 'rebound', 'Later meeting', 'college entrance examination', 'All', 'CCTV', 'comprehensive', 'scar', 'physical examination', 'No', 'remind', 'data', 'display', 'constitution', 'Bingbing' | Data show that more than 80% of high school students in China are myopic, and many of them will have myopia surgery after the college entrance examination; ② , myopia may rebound after surgery; ③ A comprehensive physical examination should be done before operation |
| 90 | 66 | 0 | effusion ', 'pleural', 'pericardium', 'Ponding', 'pleura', 'Tuberculous', 'pleurisy', 'pump', 'treat', 'ascites', 'cost', 'Lungs', 'etiology', 'abdominal cavity', 'mode', 'Dyspnea', 'puncture', 'drainage', 'Exudative', 'carry out' | My father's lung thickening caused by long-term smoking and pleural effusion caused by fever. How can I treat it? . |
| 91 | 66 | 3 | participate in', 'weight', 'subsidy ', 'follow-up', 'Shanghai', 'ct', 'subsidy', 'Boys', 'nucleic acid', 'screen', 'physical examination', 'smokeless', 'Girls', 'Check in', 'Blood collection', 'kilogram', 'one thousand eight hundred and forty-five', 'requirement', 'One year old', '06' | Male: 8022 yuan, female: 8547 yuan follow-up for 7 days (6/14) online chest CT, no B-ultrasound, smoke-free examination drug syhx1901 tablets, indications: systemic lupus erythematosus, rheumatoid arthritis (previously participated in the screening of this project last year, please do not sign up) 【 nutritional allowance 】 male: 8022 yuan, female: 8547 yuan (hospital accounts for two weeks) 【 process 】 physical examination 2.3 days notice on June 12 at home nucleic acid or antigen (upload epidemic detection) on June 13 in a nearby hospital (must be a hospital community hospital can also) do nucleic acid 【 paid retain invoice reimbursement 】 with the second nucleic acid on June 12.13 came to Huashan West Hospital for screening on June 14. Informed, screened, nucleic acid, fasting on the morning of June 15, blood collection, urine retention qualified, unified 16 CT Nucleic acid single dose group: from June 17 to 24, the follow-up ended in the morning of July 1. description blood collection subsidy is 100 yuan, CT subsidy is 50 yuan, and the green health code is required. The requirements are as follows: 1. healthy male students aged 18-45 years old, male weight ≥ 50 kg; Female weight ≥ 45 kg; BMI between 18 and 26 2. no infectious viral diseases such as hepatitis B and C, healthy, and no history of drug allergy; No history of pulmonary tuberculosis 3 do not smoke, or prohibit smoking during hospitalization; Did not participate in other clinical trials or donate blood within three months. |
| 92 | 64 | 2 | Misdiagnosis', 'Most easily', 'Harvard University', 'Seven species', 'myalgia ', 'Chyle', 'Parkinson', 'summary', 'fibre', 'Sclerosis', 'forty', 'Decrement', 'fatigue', 'Polydactyly', 'professor', 'thyroid', 'medicine', 'diagnosis', 'find' | According to clinical findings, about 40% of the initial diagnosis of the disease will be misdiagnosed. Harvard University medical professor summed up the "seven most easily misdiagnosed diseases": ① Parkinson's disease. ② . ③ Fibromyalgia. ④ Multiple sclerosis. ⑤ Celiac disease. ⑥ Chronic fatigue syndrome. ⑦ Lupus erythematosus. |
| 93 | 64 | 4 | Friends', 'good morning', 'Get up early', 'Punch in', 'Every day', 'weekend', 'Xiaofen', 'rheumatism', 'happy', 'Xiaonian', 'today', 'holiday', 'five one', 'rain', 'Systematic', 'Monday', 'loose', 'Bone', 'Rain gear', 'go out' | Tomorrow is a happy weekend |
| 94 | 60 | 2 | university student', 'Hyperthermia', 'twenty-one', 'repeatedly', 'diagnose', 'flea', 'fever', 'This disease', 'Summer heat', 'Netease', 'anti-intellectual ', 'Lupus erythematosus', 'Petrel', 'girl', 'upset', 'Girls', 'mask', 'Tuan Tuan', 'See a doctor' | How to prevent this |
| 95 | 59 | 0 | Dubai', 'dermatitis', 'universal', 'Lichen', 'flat', 'Obstinacy', 'Red tube', 'Neurogenic', 'Dandruff', 'Discoid', 'psoriasis', 'eczema', 'Indications', 'Sex dermatitis', 'identical', 'psoriasis', 'usage' | Dubai people call it universal skin cream to treat various psoriasis (psoriasis), intractable eczema, neurodermatitis, seborrheic dermatitis, insect bite dermatitis, lichen planus, discoid lupus erythematosus. It is effective for dandruff. Red tube pregnant women can use the same efficacy, usage and dosage of external application on the affected part of children can also be used |
| 96 | 59 | 0 | Shi Rui', 'Sun Wei', 'Poisoning', 'file', 'intelligent', 'China', 'Huawei', 'all nations', 'United States', 'investment', 'Billion yuan', 'Auspicious snow', 'interpreter', 'Zhu Ling', 'victim', 'company', 'murderer', 'one hundred and ninety-nine thousand eight hundred and thirty-two', 'investor', 'meeting' | Obutinib is the first and only approved Btk inhibitor targeting MZL in China. In terms of indications for self immunization, ITP is in clinical phase III, and it is expected to complete patient recruitment by the end of 2024 or early 2025. The phase IIB clinical trial of systemic lupus erythematosus (SLE) is in progress, and the patient recruitment has been nearly completed by August 20, 2024. |
| 97 | 57 | 1 | herpes zoster', 'varicella', 'Neuralgia', 'virus', 'Latent', 'inoculation', 'crowd', 'ganglion', 'vaccine', 'nerve', 'Low', 'pain', 'herpes', 'fifty', 'immunity', 'infect', 'prevent', 'Blister', 'more than' | Describe the situation after suffering from herpes zoster: "it hurts so much that your eyes are dazzled and you can't see it." when it hurts the most, it's a bit swirling. It hurts everywhere, as if your intestines are all painful... " |
| 98 | 57 | 3 | chrysanthemum', 'Pseudostellaria heterophylla', 'Cassia seed', 'hawthorn', 'dandelion', 'Yam', 'icon', 'Lotus seed', 'Euryale ferox seed', 'American ginseng', 'three seven', 'honeysuckle', 'Spleen deficiency', 'Ophiopogon japonicus', 'Poria cocos', 'Astragalus membranaceus', 'Fat sea', 'Chicory' | Lupus erythematosus - shikonin + cornu antelope powder + Fangfeng. |
| 99 | 57 | 0 | loose', 'Bone', 'cortisol', 'fracture', 'Hyperthyroidism', 'Parathyroid gland', 'Several categories', 'Dyspepsia', 'lose', 'bones', 'Osteoporosis', 'abnormal', 'hyperthyroidism', 'Rheumatic', 'long-term', 'nervous system', 'Wind like', 'diarrhea', 'cause', 'nerve' | Experts said that endocrine diseases easily lead to osteoporosis, such as the most common hyperthyroidism, hyperparathyroidism, hypercortisolism, etc., in addition to dyspepsia, long-term chronic diarrhea, etc., as well as rheumatic diseases, such as rheumatoid arthritis, lupus erythematosus, and neurological diseases, muscle abnormalities, nerve abnormalities, can lead to bone loss. . |
| 100 | 56 | 2 | Three major', 'Difficult disease', 'It will', 'What kind', 'organ', 'Central nervous system', 'Cardiopulmonary', 'intervene', 'News Network', 'Called', 'damage', 'Multiple', 'world', 'Immune disease', 'heart', 'except', 'attack', 'what', 'relieve', 'method' | Lupus erythematosus is an immune disease, not a skin disease, which attacks many organs such as heart, lung and kidney. Lupus erythematosus was once known as the world's three major difficult diseases. It can affect many organs of the body, such as the heart, lungs and kidneys, and may also cause damage to the central nervous system in severe cases. What kind of people are prone to lupus erythematosus? In addition to drug intervention, what other methods can alleviate the disease? |
| 101 | 56 | 0 | thin', 'Vigilance', 'Lymph node', 'weak', 'See', 'muscle', 'video', 'rash', 'Swelling and pain', 'express', 'Oral ulcer', 'symptom', 'signal', 'fever', 'expert', 'Life circle', 'appear', 'suffer from', 'prompt', 'what' | What are the signals of systemic lupus erythematosus? Experts said that we should be alert to these eight symptoms! ① Emaciation; ② ; ③ Joint and muscle pain; ④ Alopecia; ⑤ Oral ulcer; ⑥ Fever; ⑦ Lymph node swelling and pain; ⑧ A rash appeared. |
| 102 | 56 | 1 | aggression', 'describe', '3d', 'animation', 'resist', 'foreign', 'Disease should', 'medicine', 'common', 'introduce', 'Popular science', 'lupus', 'as well as', 'What to do', 'immune system', 'video', 'Immunity', 'cr', 'self', 'media coverage ' | What should I do with lupus erythematosus? This 3D medical animation depicts how the immune system resists foreign aggression, as well as common autoimmune diseases, such as lupus. What is lupus erythematosus? It also introduces the common symptoms and treatment of lupus erythematosus? |
| 103 | 55 | 1 | ibuprofen', 'take', 'Use with caution', 'Disable', 'dysmenorrhea', 'asthma', 'Anti inflammatory drugs', 'Antipyretic', 'All', 'Hemorrhagic', 'potential', 'Canker disease', 'extend', 'nasal polyp', 'Non steroidal', 'digestive tract', 'cautious', 'pregnant woman', 'old people' | Which groups should be forbidden to use ibuprofen with caution? Ibuprofen is commonly used to relieve moderate pain, such as muscle pain, toothache, arthralgia, headache, neuralgia, rheumatoid arthritis, periarthritis of shoulder and other diseases, and is also used to treat fever caused by the common cold or influenza? 1. The third week to the end of the third month of pregnancy of pregnant women and lactating women is the period of differentiation and formation of embryonic organs, which belongs to the "teratogenic highly sensitive period". Therefore, at this time, whether ibuprofen or other drugs, have a great impact on the development of the fetus. Ibuprofen can prolong pregnancy, cause dystocia or prolonged labor process in pregnant women in the third trimester of pregnancy; After the lactating mother takes ibuprofen, the drug can be absorbed by the baby through the milk. Therefore, pregnant women and lactating women should ban ibuprofen. 2. Allergic rhinitis, asthma, nasal polyps people with allergic rhinitis, asthma, nasal polyps should be cautious when using ibuprofen for the first time, because ibuprofen has the potential risk of inducing bronchospasm, which can aggravate or induce asthma, especially for children with moderate and mild asthma. 3. Ibuprofen can only be used as an antipyretic analgesic for patients with renal insufficiency for a short time. If ibuprofen is used in large quantities for a long time, renal function damage may occur, so ibuprofen should be used with caution for patients with renal insufficiency. 4. Ibuprofen can prolong bleeding time or aggravate bleeding tendency in patients with hemophilia or other bleeding diseases, so patients with hemophilia or other bleeding diseases should be cautious with ibuprofen. 5. . 6. Frail and elderly people should use ibuprofen cautiously, mainly because the elderly's physique is reduced, which may be accompanied by arteriosclerosis, cardiac function decline, liver and kidney function decline and other chronic diseases. Taking normal doses of ibuprofen may also cause adverse drug reactions. 7. Patients with a history of peptic ulcer or potential peptic ulcer are prone to gastrointestinal side effects when taking ibuprofen, including new ulcers, so people with a history of peptic ulcer or potential risk factors should be cautious. 8. Ibuprofen is an antipyretic, analgesic, and anti-inflammatory drug for people allergic to other NSAIDs, and it should also be forbidden for people allergic to the same kind of drugs, such as aspirin, dipyrone, indomethacin, and butazone. Ibuprofen should not be taken for a long time. It is generally used for pain relief for no more than 5 days and for antipyretic for no more than 3 days. After taking ibuprofen, if there is any discomfort, stop or consult a doctor. |
| 104 | 54 | 5 | lifeline', 'salvation', 'blue', 'girl', 'Painful', 'nine thousand nine hundred and fifty-eight', 'contribution', 'Psychosis', 'urgent', 'butterfly', 'project', 'success', 'thirteen', 'core', 'warm', 'children', 'Abandonment of treatment', 'together', 'Pieces', 'Shuicheng' | Give someone roses, and your hands will smell fragrant. I made a successful donation to the "Butterfly Girl to be rescued" project! |
| 105 | 53 | 0 | activity', 'Moldy', 'abuse', 'fermentation', 'Bad luck', 'in vivo', 'food', 'form', 'Chemical action', 'digestive enzyme', 'Slimming drugs', 'ice cream', 'Cold drink', 'two types', 'decline', 'allergy', 'temperature', 'This is', 'body' | Food in our body is divided into two forms: there are enzymes called fermentation, people are healthy; If there is no enzyme called moldy, people will be unlucky enlightenment: food in our body is divided into two forms, because our body has digestive enzymes, metabolic enzymes and food produce chemical reactions, and the participation of enzymes is called fermentation, people are healthy; If there is no enzyme, it is called moldy, and people are unlucky, it is called allergy. Under what circumstances, the activity of enzymes in the human body will decrease. One is the temperature drop. Also, the abuse of antibiotics, the abuse of weight-loss drugs, anger, overeating, will cause the reduction of physical activity enzymes, and the decline of activity. Therefore, it has caused many diseases with immune system disorders, such as allergy, lupus erythematosus, cancer, etc. to eat less cold ice cream, to supplement the body with probiotics, to eat more vegetable foods, to increase exercise, to improve our body immunity, which is the real prevention. |
| 106 | 52 | 0 | Ganoderma lucidum', 'wild', 'Thin tree', 'enhance', 'effect', 'tumour', 'efficacy', 'macrophage', 'Spore powder', 'Tranquilizing', 'stabilize the will', 'Triterpenoids', 'immunity', 'Conditioning', 'cell', 'Polysaccharide', 'm1', 'Glycopeptides', 'Malnutrition', 'Special effects' | Introduction clinical verification shows that Ganoderma lucidum can directly or indirectly treat more than 300 diseases, among which the effectiveness of taking Ganoderma lucidum is higher in five groups. 1. For example, various tumors, cancers, various liver diseases, insomnia, coronary heart disease, hypertension, hyperlipidemia, diabetes, chronic bronchitis, asthma, stomach disease, nephritis, allergy, fatigue, gout, Alzheimer's disease, menopausal syndrome, and even lupus erythematosus, and it is effective in improving the symptoms of various diseases, while Ganoderma lucidum has better preventive effect. Because Ganoderma lucidum can "strengthen health, balance yin and Yang, and keep the body stable and healthy!" from the perspective of medicine, all diseases (including cancer) belong to immune function defects. 2. Traditional Chinese medicine has the theory of "supporting the vital and eliminating the pathogenic factors", which believes that as long as the healthy qi in the body is strong in immune function, the evil Qi is the evil of wind, cold, heat, dampness, dryness and fire, and pathogenic factors such as bacteria and viruses will not damage the body and lead to various diseases; If the immune function is weak, evil Qi enters the body and destroys the balance of yin and Yang in the body, disease will occur, which is the root of all diseases. 3. Ganoderma lucidum can strengthen the body and eliminate pathogens, strengthen the foundation and nourish the yuan, and adjust the internal environment of the body to maintain balance, stability and health all the time. This is closely related to the fact that Ganoderma lucidum can improve the number and activity of immune cells in our body and regulate the balance of humoral immunity. Therefore, Ganoderma lucidum is very effective for various sub-health, chronic diseases, cancer and other people, especially for five categories of people: 1. Many patients are weak, easy to catch cold, often dizzy, headache, fear of cold, sweating, easy to fatigue, sleepiness 2. People with neurasthenia and loss of sleep. Ganoderma lucidum acid can excite the central nervous system, and tryptophan can regulate and supplement natural melatonin to induce good sleep. 3. . 4. Ganoderma lucidum inhibits "bad blood lipids", improves "good blood lipids", and regulates blood lipid balance in people with cardiovascular and cerebrovascular diseases; Ganoderma lucidum can promote insulin secretion, reduce blood glucose, and effectively improve diabetic complications. 5. Ganoderma lucidum polysaccharide can indirectly kill tumor cells by increasing the number and activity of immune cells in various tumor and cancer populations; Ganoderic acid can inhibit the activity and growth of tumor cells and block tumor angiogenesis. What is the most important, the body is the most important don't hesitate, eat Ganoderma lucidum now! |
| 107 | 52 | 1 | Aniru', 'Monoclonal antibody', 'Headline', 'article', 'recruit', 'release', 'clinical', 'Target', 'principle', 'anifrolumab', 'Lupus erythematosus', 'Medication', 'new drug', 'abroad', 'existing', 'medicine', 'Biological agents', 'treat', 'cease', 'curative effect' | The efficacy of anilumab in the treatment of lupus erythematosus. |
| 108 | 51 | 3 | Amitabha', 'Chant Buddha', 'Amitabha', 'Reincarnation', 'ultimate reality', 'Gratitude', 'Previous life', 'achievement', 'Release', 'Lay person', 'interfere', 'others', 'Shoujing', 'How ugly', 'Immeasurable', 'Dharma', 'Bodhisattva', 'Merit', 'put down' | Her fate was also very special. She had lupus erythematosus, which was more serious than cancer. The doctor told her that she could die at any time and asked her to be mentally prepared. Good luck! |
| 109 | 50 | 2 | He Jie', 'Newly married', 'Cheat marriage', 'divorce', 'husband', 'Accusing', 'Find out', 'Chongqing', 'Deceive', 'woman', 'Face changing', 'honeymoon', 'temporary', 'Incurable disease', 'force', 'open', 'Radical cure', 'Split bed', 'requirement' | He Jie, a Chongqing woman who was enjoying her wedding honeymoon, was suddenly found to have systemic lupus erythematosus. The disease cannot be cured temporarily, and children cannot be born until the disease is stable. The mother-in-law instantly changed her face and forced her to divorce her husband. The husband also felt cheated and even publicly accused him of cheating on marriage. |
| 110 | 50 | 1 | monologue', 'Taurus', 'diary', 'Kabu', 'Make a list', 'new song', 'Zhoushen', 'Asia', 'Systematic', 'Ferroptosis', 'Xiongguan', 'happy', 'Lupus erythematosus', 'Should be', 'even if', 'Not as good as', 'travel', 'never', 'forever', 'time' | Our understanding of ourselves is more important than what we are |
| 111 | 50 | 1 | nation', 'Pride', 'Barbell', 'Handsome', 'laughter', 'Ghost horse', 'Chrysanthemum parvifolium l.var.parvifolium l.var.parvifolium l.var.parvifolium l.var.parvifolium l.var.parvifolium l.var.parvifolium', 'great', 'tiger', 'Awesome', 'momo', 'Childe', 'Oh, my God', 'Ming Jiaming', 'Mingjia', 'lemon', 'scientist', 'As beautiful as flowers' | National pride, great female scientist! |
| 112 | 48 | 5 | daughter', 'deliver vegetables', 'Pyramid selling', 'Beijing', 'one thousand and seven hundred', 'span', 'kilometre', 'Son', 'old husband and wife', 'spend', 'Shooting customers', 'visit', 'a pair', 'Ever entered', 'countless', 'move', 'villager' | The video of an old couple who traveled 1700 kilometers to Beijing to deliver vegetables to their sick daughter has touched countless netizens. |
| 113 | 48 | 4 | Spinous process', 'nerve cell', 'storm', 'mitochondria', 'Cerebral peduncle', 'induce', 'myocarditis', 'disorder', 'tubercle', 'factory', 'cell factor', 'May cause', 'spinal cord', 'leukemia', 'Purpura', 'vaccine', 'tinnitus', 'protein', 'inflammation', 'urticaria' | The spike protein in the vaccine can attack human nerve cells, cause mitochondrial (cell energy factory) damage, induce cytokine storm, cause immune system disorder, and cause spinal cord inflammation, such as urticaria, alopecia, pulmonary nodules, allergic purpura, lupus erythematosus, leukemia, myocarditis, cerebral infarction, tinnitus, menstrual disorder, etc |
| 114 | 47 | 4 | pulmonary fibrosis', 'Interstitial', 'Lungs', 'Interstitial', 'tubercle', 'fibrosis', 'Alveoli', 'pneumonia', 'Pulmonary flaccidity', 'Idiopathic', 'Dyspnea', 'connective tissue', 'dust', 'Pulmonary disease', 'quot', 'breathing', 'asbestos', 'Dry cough', 'inhalation', 'cause' | Zhang Yanping, a respiratory expert at Xiyuan Hospital of the Chinese Academy of traditional Chinese medicine, said: there are several misunderstandings in the understanding of pulmonary fibrosis. Recently, it has been found that many people's understanding of pulmonary fibrosis is still unclear through the treatment of patients and online. Here, expert Zhang Yanping, in combination with her personal clinical experience, talks about how to correctly understand pulmonary fibrosis, so that the disease can be accurately diagnosed and reasonably treated on several issues that are easy to cause misunderstanding. 1： Pulmonary fibrosis does not refer to a single disease. The term "pulmonary fibrosis" does not refer to an independent disease, but a general term for a large group of diseases. In medicine, pulmonary fibrosis is attributed to diffuse interstitial lung disease (so-called interstitial lung disease), which is also known as diffuse parenchymal lung disease in recent years. There are many reasons for pulmonary fibrosis, common environmental, occupational, physical and chemical factors, such as asbestos, mineral dust, chemotherapy drugs, radiation injury, harmful gas inhalation, etc. Exogenous allergic alveolitis caused by contact with pigeon dung, animal fur, moldy subtilis, etc., can also lead to pulmonary fibrosis. For those with unknown causes, they are collectively referred to as idiopathic interstitial pneumonia, including the often said idiopathic pulmonary fibrosis. Only through a variety of examinations, combined with clinical manifestations, can doctors clarify which type of pulmonary fibrosis belongs to and what causes it may be. Only with a clear diagnosis can we talk about reasonable treatment. 2： Not all pulmonary fibrosis has no way to cure. I often hear people say that "there is no medicine for pulmonary fibrosis" or even "it can only live for 5 years at most". This statement is not comprehensive and scientific enough. If this view is reasonable, I think it should refer to the so-called "idiopathic pulmonary fibrosis". Idiopathic pulmonary fibrosis is one of the many types of "pulmonary fibrosis". The cause is unknown. It has typical clinical manifestations, chest X-ray and CT features, and is familiar to respiratory doctors. Clinical observations abroad have found that the median survival of patients is about 5 years, which is only a statistical figure; In fact, many patients survive for far more than 5 years! In particular, the above understanding is limited to idiopathic pulmonary fibrosis! In fact, most cases of pulmonary fibrosis found clinically are not idiopathic pulmonary fibrosis, but secondary pulmonary fibrosis. After encountering pulmonary fibrosis, the most important task for doctors is to clarify whether it is idiopathic or secondary. For example, if it is clear that pulmonary fibrosis is caused by rheumatic immune diseases, the focus of treatment should be on the primary disease. Pulmonary fibrosis caused by some environmental factors can remain relatively stable for a long time after leaving the corresponding environment. 3： . Chest X-ray and lung CT are the most important first step in the diagnosis of pulmonary fibrosis / interstitial lung disease, and there are also misdiagnoses in this first step. Zhang Yanping, an expert, has seen many times in clinical practice that patients with lung CT showed "interstitial changes" due to insufficient inspiration or breath holding, or suspected "diffuse shadows" due to poor CT shooting conditions, and suspected pulmonary interstitial disease not only brought unnecessary concerns, but also may accept some unnecessary auxiliary examinations. In fact, pulmonary fibrosis / interstitial lung disease is a large group of diseases that are difficult to diagnose in the respiratory department. Doctors need not only rich experience and knowledge of related diseases, but also a variety of other auxiliary examinations (including laboratory examinations). Moreover, these examinations must be reasonable and the results must be accurate in order to make a comprehensive analysis of the disease and finally make a correct diagnosis. There are many auxiliary examinations mentioned above, including lung function tests, a variety of blood tests, bronchoscopy related examinations, and even lung biopsy. Zhang Yanping's expert tips: for the above reasons, it is generally recommended that patients with "pulmonary fibrosis / interstitial lung disease" should be further diagnosed by doctors with corresponding clinical experience to avoid misdiagnosis and mistreatment. |
| 115 | 47 | 0 | Stay up late', 'imbalance', 'Come to the door', 'Sub health', 'function', 'long-term', 'performance', 'gastroenteritis', 'human body', 'body', 'harm', 'state', 'digestive tract', 'influence', 'Also', 'damage', 'respiratory tract', 'sleep', 'In' | Easy to cause immune dysfunction: dark circles and mental fatigue are only superficial symptoms caused by staying up late. In fact, the more terrible harm of staying up late for a long time is the immune dysfunction of the body. The immune dysfunction will produce nephritis in the kidney, lupus erythematosus in the skin, and rheumatoid arthritis in the joints. At the same time, the body's immune capacity will also be reduced, making the human body in a serious sub-health state. Respiratory diseases such as colds, gastroenteritis and other gastrointestinal diseases will come to the door. Skin damage: staying up late will directly damage the skin, because the skin will be repaired and maintained after the period from 10 pm to 2 am. Staying up late for a long time will lead to endocrine disorders and affect the circulation of the nervous system. In the long run, the skin will be dry and lack of luster, and endocrine disorders will also lead to acne and blackheads, so the damage to the skin is the most direct. Disrupting the circadian clock: the human body has a normal circadian clock. If you often stay up late, it will make the circadian clock disordered. In the long run, fatigue and mental distress will occur, which will directly reflect on the human body's resistance. If you stay up late, it will also lead to various diseases. Cause neurasthenia, headache and insomnia: People's sympathetic nerves should rest at night and be excited during the day, so as to support people's work throughout the day, while those who stay up late are excited at night. . For a long time, there will be neurasthenia, insomnia and other problems. So we should pay attention to staying up late as little as possible. If you stay up late, you should remedy it in time. |
| 116 | 45 | 2 | Zhou Haimei', 'Live vaccines', 'Death', 'Death', 'woman', 'twelve', 'Cliff', 'news', 'Lupus erythematosus', 'follow', 'men and women', 'one kind', 'childbearing age', 'patient', 'Preference', 'self', 'prevent', 'vaccine', 'birthday' | ① With the news of Zhou Haimei's death, the survival status of patients with systemic lupus erythematosus also attracted attention. After all, according to the status of social media, Zhou Haimei celebrated her 57th birthday on December 6. If she finally died due to complications induced by systemic lupus erythematosus, it also means that the fatal risk of systemic lupus erythematosus disease may be much higher than the traditional public perception of "no harm for chronic diseases". ② The 10-year survival rate of domestic patients with systemic lupus erythematosus has risen to 89%; After 25-30 years, the survival rate showed a precipitous decline, only 30%. This also shows that the long-term survival rate of patients with systemic lupus erythematosus is not optimistic. ③ The patients were mainly women of childbearing age. The ratio of male to female was 1:11.9, and the average age of onset was 30.7 years old. The average age at diagnosis was 31.6 years old, and the average diagnosis delay was 10.8 months. This shows that women should pay more attention to this kind of disease. |
| 117 | 44 | 0 | Ichthyosis', 'psoriasis', 'cure', 'acne', 'urticaria', 'Course of treatment', 'State Intellectual Property Office', 'The present invention', 'Short course of treatment', 'Quick effect', 'sterilization', 'research institute', 'Made', 'have already', 'invention', 'Effective', 'advantage', 'international' | A drug that can cure psoriasis, acne lupus erythematosus, and ichthyosis urticaria skin No. 1 China International Academy of medicine has invented a drug that can cure psoriasis, acne lupus erythematosus, and ichthyosis urticaria skin No. 1, and has applied for an invention patent through China's State Intellectual Property Office. The invention relates to a drug skin No. 1 which can cure psoriasis, acne, lupus erythematosus, ichthyosis and urticaria. It is made of pure Chinese medicine and has many advantages, such as good therapeutic effect, small side effects, short course of treatment, etc; It has the effects of activating blood circulation, removing blood stasis, sterilizing and relieving itching, and enhancing human body's own immunity. Treat psoriasis (psoriasis), acne, lupus erythematosus, ichthyosis, urticaria and other skin diseases. It has the characteristics of quick effect and no recurrence after cure. Generally, one course of treatment is effective, and about three courses of treatment can cure. |
| 118 | 44 | 0 | Bone', 'loose', 'Osteoporosis', 'fracture', 'Bone mass', 'absorb', 'bones', 'proton pump', 'bone density', 'increase', 'insulin', 'motion', 'menopause', 'Anticoagulants', 'Corticosteroids', 'inhibition', 'Sensitizer', 'spine', 'formation', 'promote' | Osteoporosis (OP) is a systemic bone disease, which is characterized by decreased bone mass and destruction of bone microstructure, resulting in decreased bone strength, increased fragility, and prone to fracture. Fractures can occur in minor trauma, and those with severe osteoporosis can occur when turning over naturally, bending down and coughing. Organic matter forms a frame, and minerals fill it to form tangible bone. The interior of bone is a complex structure. Osteopenia refers to the reduction of mineral and bone organic matter in bone. Bone mass is commonly expressed by bone mineral density in clinic. There are different methods to detect bone mineral density, including ultrasound CT、 Dual energy X-ray is internationally recognized to detect bone mineral density. The destruction of bone tissue microstructure, that is, the destruction of bone frame structure, is manifested in the absorption of bone trabeculae formed by organic matter in bone becoming thinner and broken, resulting in a reduction in the number, thinning and porous bone. The hardness and toughness of bone are decreased, and it is prone to fracture. There are many etiologies of osteoporosis, which have not yet been finally clarified. The main co morbidity factors can be divided into five aspects: ① immune factors; ② ; ③ Physical factors; ④ Endocrine factors; ⑤ Genetic factors. Osteoporosis 1. different onset rheumatic immune diseases are chronic inflammatory diseases caused by autoimmune disorders. Rheumatic disease-related osteoporosis has three aspects: chronic inflammation, reduced activity, and hormone application. Inflammatory lesions and osteoporosis the increase of various inflammatory factors, such as interleukin-1, interleukin-6 and tumor necrosis factor, leads to increased bone destruction and bone loss, thus causing osteoporosis. The incidence of osteoporosis in rheumatoid arthritis and systemic lupus erythematosus is as high as 40%. The fracture caused by osteoporosis in patients with rheumatoid arthritis is 2.25 times that of patients without rheumatoid arthritis in different ages and genders. Chronic inflammation caused by spondyloarthritis and idiopathic myositis is also involved in the occurrence of osteoporosis. Exercise loss and osteoporosis exercise loss often cause whole-body bone loss. Exercise can also induce hormone changes to improve bone metabolism. A large number of studies have shown that exercise intensity and exercise volume are related to the increase of estrogen level in the body, and the effect on the increase of estrogen continues until after exercise intervention; The regulation of exercise on bone metabolism signaling pathway, domestic and foreign studies have shown that appropriate physical exercise can up regulate the key factors in Wnt, BMP, opg/rank/rankl and other signaling pathways, which is conducive to bone formation and promote bone growth and development. It can be seen that the influence of exercise on bone is multifaceted, but most rheumatic diseases affect the body activity due to the involvement of joint muscles, thus promoting the occurrence of osteoporosis. Glucocorticoid and osteoporosis glucocorticoid induced osteoporosis is widely recognized. Whether a single high dose or a high cumulative dose, it increases the risk of fracture. Glucocorticoid mainly causes the reduction of cancellous bone and trabecular bone mass, but has relatively little effect on cortical bone, so it is particularly prone to vertebral fracture. In a study of bone tissue structure in children with long-term application of hormones to control rheumatic diseases, the changes were mainly decreased trabecular retroversion, decreased thickness of uncalcified bone, thinning of osteoblast layer and increased trabecular bone interval. 2. different treatments the main factor of rheumatism related osteoporosis is the disease activity. The disease activity not only increases the local bone erosion and local bone resorption, but also aggravates the bone loss due to pain and swelling, which limits the activity. Most diseases need glucocorticoid treatment to aggravate the risk of osteoporosis and fracture from the aspect of exogenous factors. Effective control of inflammation inflammatory control can significantly improve osteoporosis. In the inflammatory state, the bone of patients with arthritis will decalcify, without bone mineral density. When clapping hands or spine x, it will be found that the bone will become black and the bone mass will be reduced. Effective treatment of inflammation in rheumatic immune disease is the premise of rheumatic immune disease combined with osteoporosis. Standardized functional exercise at present, the contents of exercise against osteoporosis include aerobic exercise, progressive resistance training, impact exercise, weight-bearing exercise, national traditional fitness exercise, combined exercise and vibration training. For patients with rheumatic diseases, it is not suitable for impact exercise and weight-bearing exercise. With disease control and improvement of joint and body conditions, combined exercise and professional guidance downward vibration training, such as walking and Tai Chi, are gradually added. Osteoporosis is prone to occur in obese and emaciated people. Reasonable dietary adjustment of body weight is beneficial to prevent the occurrence of osteoporosis. Daily 20 minutes of sunshine can promote the activation of vitamin D, which can promote intestinal calcium absorption and bone metabolism. Clinical application of anti osteoporosis drugs drugs for the treatment of osteoporosis can be divided into bone resorption inhibitors, bone formation stimulators, bone mineralization enhancers. These include estrogen, calcitonin, diphosphates, parathyroid hormone, calcium and vitamin D and their derivatives. |
| 119 | 44 | 3 | nine thousand nine hundred and fifty-eight', 'Kill', 'clause', 'acupuncture', 'kind-heartedness', 'Children', 'Sub donation', 'Children's Charity Association', 'Life should not be lost', 'Charities', 'wamq0528', 'Figure V', 'Draft', 'Funding', 'Cat mother', 'away from', 'format', 'Balance', 'Enlighten' | There are also ways to actively kill those who should not die, such as "subsidizing" lupus erythematosus patients to change acupuncture treatment |
| 120 | 43 | 3 | Rain bean', 'Kidney bean', 'Comparable', 'married', 'transfer', 'To be honest', 'Too many', 'fireworks', 'Sleepless', 'bottled', 'overwhelming majority', 'Jiajia', 'sound made by a hen', 'No.2', 'Three', 'Sledgehammer', 'fall in love with', 'Overbearing', 'vicious', 'want' | To tell the truth, if Zhou Haimei wants to be accompanied, there are too many people who can be transferred than most married women, and there are also too many professionals |
| 121 | 43 | 3 | stammering', 'Liu Wei', 'moxibustion', 'insist on', 'Trough', 'Fiance', 'Protagonist', 'Conditioning', 'answer', 'Documentary', 'Positive solution', 'believe', 'harvest', 'Tagore', 'recollect', 'subscription', 'story', 'self-confidence', 'abandon' | "Believe not because of seeing, but because of believing". Many answers of life are in Tagore's words - in the second issue of our documentary "because of AI", the protagonist Liu Wei regained confidence from lupus erythematosus hormone obesity to health gain, and found her positive solution with "Ai" and "persistence". |
| 122 | 42 | 3 | herpes zoster', 'crowd', 'Suffer from', 'one hundred and fifty-six', 'Most painful', 'according to the statistics', 'Age', 'forty-one', 'More importantly', 'Zhonghui', 'risk', 'High risk', 'ten thousand people', 'one-third', 'fifty', 'Illness', 'annually', 'dangerous' | The most painful skin disease. Experts said that age is the most important risk factor for herpes zoster. According to statistics, there are about 1.56 million new cases of herpes zoster in people over 50 years old in China every year. The older the age, the more likely the disease is and the more serious the disease is. In addition, patients with diabetes, chronic kidney disease, chronic obstructive pulmonary disease and other chronic diseases have an increased risk of herpes zoster by 24% to 41%; Patients with autoimmune diseases, such as rheumatoid arthritis and systemic lupus erythematosus, have a 2.3 to 4 times higher risk of herpes zoster than the general population. |
| 123 | 42 | 3 | our country', 'recrudescence', 'Peking Union Medical College Hospital', 'Years old', 'Hit hard', 'five thousand five hundred and sixty', 'Age', 'times', 'introduce', 'morbidity', 'patient', 'Zhangzhiyi', 'Immunology Department', 'three hundred and sixteen', 'three hundred and seven', 'half', 'every time', 'Limengtao' | . According to the health times: in 2009, the Department of Rheumatology and immunology of Peking Union Medical College Hospital led the establishment of the China systemic lupus erythematosus research collaboration group in China to investigate the epidemiological characteristics of lupus erythematosus patients in China. The data showed that the average age of onset of systemic lupus erythematosus patients in China was 30.7 years old, with a female to male ratio of 12:1. The average age at diagnosis was 31.6 years old, of which 4.58% were aged &lt; 18 years old; 79.56% of patients aged 18-40 years old, reflecting that the vast majority of patients with systemic lupus erythematosus in China are young people. In terms of geographical distribution, the northern region of China has the largest number of patients with systemic lupus erythematosus, accounting for 25.03%; Patients with systemic lupus erythematosus who have low education level, poor local medical level, long sunshine time and live in the south of China have higher disease severity. In addition, both UV irradiation and smoking can induce the onset and recurrence of systemic lupus erythematosus, and smokers are more serious and prone to renal damage. "Systemic lupus erythematosus is an autoimmune disease caused by multiple factors, and the pathogenic factors include genetics, cyclins, gender, sex hormones, UV environment, etc., so the disease is highly heterogeneous and the requirements for individualized diagnosis and treatment are high." Professor Zeng Xiaofeng, director of Rheumatology and Immunology Department of Peking Union Medical College Hospital, Chinese Academy of Medical Sciences, introduced in a health newspaper article in April 2020, "the cruel reality is that more than half of the patients with onset age around 30 years old in China will die by the age of 55~60 years." "relapse is the most difficult obstacle for lupus erythematosus patients to face in their life, and many patients are constantly consumed and injured in the 'Relapse remission relapse'." Zhang Xiaofeng, vice president of the First Affiliated Hospital of Harbin Medical University Zhiyi introduced in the wechat Journal of health times in September 2020 that organ damage would worsen once it recurred. |
| 124 | 42 | 3 | Onlookers', 'answer', 'Yuankuai', 'value', 'free', 'come quick', 'problem', 'usually celadon', 'together', 'one thousand', 'twelve thousand and eight hundred', 'Shan Guimin', 'everybody', 'Wangyuling', 'zynzztzlp', 'Kongqingdong', 'Yangxichuan', 'thirteen thousand and five hundred', 'two thousand nine hundred and ninety-nine', 'three thousand and eight hundred' | I watched the answer of @ Dr. Nie from rheumatology department. The question is worth 20.00 yuan, but only 1 yuan. Come and watch |
| 125 | 41 | 3 | Universiade', 'Eight characters', 'Jiamu', 'appearance', 'Terrestrial branch', 'Venus', 'menstruation', 'feeling', 'body', 'Xin Jin', 'Propionic fire', 'Trunk and branch', 'representative', 'Bieber', 'Giant gate', 'Ethyl wood', 'Sting' | Psoriasis in junior high school and lupus erythematosus in senior high school are both chronic diseases that have not been cured and have completely affected life and learning. Last year, my family was cheated out of all the accumulation. Now I am very confused and can't find the direction of life. |
| 126 | 41 | 2 | Preference', 'ydl', 'woman', 'No', 'estrogen', 'butterfly', 'Ha Yong', 'See you in the house', 'Play the piano', 'Furong Yijin', 'Specific drug', 'Lupus erythematosus', 'ncrdy', 'Diction', 'Too big to say', '' | Ah |
| Use the "Insert Citation" button to add citations to this document.  127 | 41 | 2 | student of average academic achievement', 'Zhang Yu', 'Daughter', 'Maternal fetus', 'I see', 'for what reason', 'Concord', 'university student', 'forty-five', 'liver cancer', 'lung cancer', 'optimistic', 'Netizen', 'mentality', 'nervous system', 'keep', 'leukemia', 'thirty', 'inheritance' | Netizens often ask why my daughter is a middle-class student, and I can maintain an optimistic attitude. As a doctor, I have seen too many patients, including nervous system diseases inherited from the mother's birth, leukemia at the age of 4-5, lupus erythematosus at the age of 4-5, liver cancer and lung cancer at the age of 30. Many of them are things around me. I don't have too many requirements for life. |
| 128 | 41 | 4 | Supervisor nurse', 'nurse', 'nursing', 'Talent Network', 'examination', 'Qualification certificate', 'Nurse', 'Knowledge points', 'health', 'measure', 'Postgraduate entrance examination', 'primary', 'Medical care', 'recruit', 'Qualification examination', 'occupation', 'China' | Knowledge points for preparing for the examination of the nurse in charge: treatment of systemic lupus erythematosus & nursing measures |
| 129 | 40 | 3 | donate blood', 'Urgent', 'firemen', 'Massive hemorrhage', 'fire protection', 'Blood bank', 'Jiangxi', 'Group', 'Surgical procedure', 'Nanchang', 'Pass the customs', 'Pregnant mother', 'chance upon', 'spontaneous', 'West Lake District', 'rescue', 'teammate', 'go to' | On February 20, in Nanchang, Jiangxi Province, a maternal with lupus erythematosus suffered from massive bleeding during the operation, and the blood bank was in urgent need of help. After seeing the emergency news, firefighters of the West Lake fire rescue brigade spontaneously organized their teammates to donate blood at the blood donation point. They said: I hope our little help can make pregnant mothers pass the test safely! ! |
| 130 | 40 | 1 | Lily', 'Labor contract', 'company', 'two thousand and twenty-two', 'court', 'building', 'Solstice', 'two thousand and twenty-one', 'Renewal', 'labour', 'lactation', 'maternity leave', 'Expiration', 'Medical period', 'sick leave', 'hear', 'relieve', 'termination', 'go to work' | On May 27, the second intermediate people's Court of Shanghai released a labor dispute caused by the approval of lactation leave. On July 10, 2015, Lily joined a construction company as a designer, and the term of labor contract is from July 10, 2019 to July 9, 2022. In 2020, Lily became pregnant. On April 30, 2021, Lily gave birth and took maternity leave until September 21, 2021. After the expiration of maternity leave, Lily applied to the company for breastfeeding leave because she still needed to continue treatment, but the company did not approve. From September 22, 2021, Lily didn't attend the work until June 6, 2022. On June 9, 2022, the construction company informed both parties that the labor contract would expire on July 9, 2022, and the company decided not to renew the labor contract. After hearing, the court held that Lily did not explicitly refuse to renew the labor contract with the company, because the nature of Lily's leave from September 22, 2021 to April 5, 2022 was lactation leave rather than sick leave, so Lily was still in medical treatment on July 7, 2022. The construction company terminates the labor contract during the medical treatment period and violates the relevant provisions of the labor contract law, which is illegal termination. |
| 131 | 40 | 3 | The third time', 'rey', 'Seed retention', 'Remarry', 'have to', 'Funny', 'Male side', 'Intensive care unit', 'receive a patient for treatment', 'lucky', 'Twice', 'Too many', 'Lily', 'Men', 'Three', 'natural', 'twenty-five' | To be funny, two women with lupus erythematosus who had to give birth to the man at the age of 25 had been treated in the hospital before. The first two spontaneous abortions left the ICU thinking that they could be lucky had a third time, the third time was directly life-threatening, and the man could remarry three months later |
| 132 | 40 | 3 | ladygaga', 'Gaga', 'Discount', 'gaga', 'aunt', 'Sudden death', 'Anal hair', 'Sandu', 'lady', 'birthday anniversary of the dead', 'Cadaveric gas', 'Top7', 'ladygaga0328', 'Fans', 'diva', 'artist', 'Qinghai Tibet Plateau', '' | Ladygaga was killed by a dog wheel on the Qinghai Tibet Plateau. The exhalation of body gas after the cremation of the body is very harmful to lupus erythematosus patients. It is said that ladygaga's aunt died suddenly after smelling this smell |
| 133 | 40 | 4 | phospholipid', 'aps', 'thrombus', 'abortion', 'Syndrome', 'antibody', 'Anti cardiac', 'gestation', 'formation', 'positive', 'aptt', 'Recurrent', 'secondary', 'repeatedly', 'vein', 'apl', 'placenta', 'antiphospholipid', 'self', 'Arteriovenous' | Antiphospholipid syndrome antiphospholipid syndrome (APS) is a disease characterized by recurrent arterial or venous thrombosis, sick pregnancy, and persistent positive antiphospholipid antibodies (APL). APS can be secondary to systemic lupus erythematosus or other autoimmune diseases |
| 134 | 40 | 3 | lorelei ', 'lorelei', 'no one', 'Children', 'bait', 'Swindle', 'Kill', 'Jinlin', 'nine thousand five hundred and fifty-eight', 'money', 'Turn around', 'clj ', 'My hand', 'persecute to death', 'alive', 'Carlyle ', 'Drag to death', 'interest', 'writer' | Nobody pays attention to it! This is to directly use the patient as bait to cheat the money of good people, and finally kill the bait! This disease is particularly painful! How much does a child bear for the benefit of adults!! |
| 135 | 39 | 5 | Save women', 'Selling house', 'War', 'lifeline', 'blue', 'nine thousand nine hundred and fifty-eight', 'contribution', 'urgent', 'father', 'success', 'project', 'salvation', 'Xiaowenrui', 'core', 'children', 'girl', 'warm', 'overcome', 'Pieces' | I hope to give a little warmth in this big world. I made a successful donation to the project "girls fight lupus erythematosus, father wants to sell a house to save women!"! |
| 136 | 39 | 1 | solve', 'apostle', 'Awesome', 'Drug resistance', 'problem', 'vigilate', 'Dark night', 'morning breeze', 'Coerce', 'Disciple', 'Tact', 'Bright moon', 'irrigation', 'Bixin', 'This is', 'amazing', 'Really', 'Drug God', 'Out of the wall' | The problem of drug resistance has been solved, and there is hope to solve lupus erythematosus. That's great! |
| 137 | 39 | 1 | nylon', 'Methylprednisone', 'change into', 'Prednisone', 'Four tablets', 'acetic acid', 'Take methylprednisone', 'Reduced to', 'Half', 'Systematic', 'Finish nail splashing', 'review', 'Replace again', 'Love each other', 'which', 'hormone', 'once', 'today', 'today', 'Five tablets' | I want to ask you, do you need to take hormone drugs for a full cycle before changing? The attending doctor changed from methylprednisolone to prednisone acetate. As a result, the last doctor prescribed me too much methylprednisolone. The attending doctor said that I could change it after eating methylprednisolone. Today is the day to reduce the dosage to four tablets. Methylprednisolone can only be taken for a few days. Should I directly change to four tablets of prednisolone acetate, or take the remaining four tablets of methylprednisolone every day and then change the medicine? |
| 138 | 39 | 3 | Bupleurum parvifolium', 'Bupleurum chinense', 'peony', 'angelica', 'Our party', 'Tanghe', 'Shaoyang', 'Prescription syndrome', 'Gypsum', 'constitution', 'Decoction syndrome', 'Half surface and half interior', 'Pinellia ternata', 'nn', 'Chaigui', 'headache', 'Poria cocos', 'Can', 'Cold and heat' | Introduction to 20 tried and tested modified and combined prescriptions of Chaihu Decoction: Chaihu Decoction is widely used, especially for common chronic diseases which often present with Chaihu syndrome. Mr. Hu Shishu is good at using Chaihu Decoction to treat various difficult diseases. This issue shares with you the 20 most commonly used modified and combined prescriptions of Chaihu Decoction in clinical practice. Most of them can achieve quick and miraculous effects when applied to appropriate symptoms. It is full of practical information and is worthy of our repeated consideration and learning. |
| 139 | 38 | 0 | Mr Lin', 'Climb a building', 'motion', 'image', 'restart', 'multilayer', 'Fujian', 'Boy', 'Wanceng', 'Layer to', 'Shopping', 'Improve', 'CongGang', 'Full of confidence', 'young', 'shadow', 'Longest', 'Cheer up' | In 2018, Mr. Lin was diagnosed with systemic lupus erythematosus. He has been ill for nearly 5 years. His condition has been repeated, his physical strength and energy have decreased, and his image has also changed greatly. Mr. Lin was deeply shocked, but seeing his wife's encouragement and thinking of his young daughter, Mr. Lin adjusted his mood and combined his own situation to "restart life". He began to climb stairs, Baduanjin and other sports. From the beginning of the first floor to later, he could exercise for 1.5 hours a day, up to 3 hours. In the past five years, he has climbed more than 100000 floors. I also hope the patients will stick to it. |
| 140 | 38 | 1 | great', 'bbc', 'scientist', 'century', 'Short film', 'Madame Curie', 'Einstein', 'If used', 'won qualification for a competition', 'No doubt', 'Selection', 'measure', 'legend', 'compere', 'quot20', 'quot', 'save', 'history', 'quotbbc' | Tu Youyou, Madame Curie, Einstein and Turing were shortlisted as the greatest scientists of the 20th century by BBC! ! |
| 141 | 38 | 1 | chloroquine', 'sulphuric acid', 'Chloramphenicol', 'Tadine', 'Don't eat', 'Two', 'One', 'Several tablets', 'Systematic', 'elbow joint', 'precipitate', 'Stop', 'excuse me', 'opinion', 'Yes no', 'Rash', 'Eyes', 'Stupid', 'Bigger' | Lupus erythematosus, hydroxychloroquine, help. |
| 142 | 38 | 3 | 15275169999', 'West Road', 'Pentapeptide', 'youth', 'thymus', 'Yaoda', 'telephone', '661', 'Lu Dao', '1257233245', 'My shop', '82921366', '053182921966', '0531', 'Daming Lake', 'qq1223371544', '56789', 'pharmacy', 'facsimile', 'Coach station' | What is the role of thymopentin in the treatment of diseases with low cellular immune function. Thymopentin can be used in regular hospitals. It is mainly used in children older than eight years with chronic hepatitis B and various primary or secondary T cell deficiency diseases. Some immune system diseases, such as rheumatoid arthritis and systemic lupus erythematosus, can also be used; It is commonly used in various diseases with low cellular immune function. Tumor patients will take this drug for adjuvant treatment, which has certain effects. It is better to go to a regular hospital and use it under the guidance of a doctor. Don't buy drugs outside blindly, so as to prevent improper use from causing adverse effects on patients and affecting their recovery. |
| 143 | 38 | 0 | Femoral head', 'necrosis', 'hip joint', 'traumatic', 'collapse', 'hormone', 'Femoral neck', 'fracture', 'Osteonecrosis', 'orthopaedics', 'dislocation', 'Alcoholism', 'Osteocyte', 'ischemic', 'occurrence', 'cause', 'long-term', 'ischemia ', 'pain', 'Blood supply' | What is "hip pain"? 1. Osteonecrosis of the femoral head osteonecrosis of the femoral head is a common disabling disease of the hip joint in China, which is due to bone circulation disorders, bone cell death, and then lead to changes in the structure of the femoral head, later collapse of the femoral head, hip pain and dysfunction. ① Traumatic: trauma leads to necrosis of the femoral head, such as femoral neck fracture, hip dislocation, hip trauma, hip sprain and contusion caused by external force impact. It can directly or indirectly damage the blood supply of the femoral head, resulting in avascular necrosis of the femoral head. Trauma is the main factor causing femoral head necrosis. However, the occurrence and extent of traumatic avascular necrosis of the femoral head mainly depend on the degree of vascular damage and the compensatory ability of collateral circulation. ② Non traumatic: there are many pathogenic factors for non traumatic femoral head necrosis, including alcoholism, after high-dose hormone therapy, after local radiotherapy, decompression sickness, systemic lupus erythematosus, coagulation dysfunction, hemoglobinopathy, and so on. Among them, alcoholism and hormone induced femoral head necrosis are the most common causes in China. Alcohol stimulation leads to necrosis of the femoral head. Due to long-term heavy drinking, alcohol accumulates in the body, resulting in increased blood lipids and damage to liver function. The rise of blood lipids causes the increase of blood viscosity, the slow down of blood flow speed, and the change of blood coagulation, which can make blood vessels blocked, bleeding or fat embolism, and cause bone necrosis. The clinical manifestations were aggravated after drinking, walking duck steps, heart failure, fatigue, abdominal pain, nausea and vomiting, etc. |
| 144 | 38 | 0 | Stay up late', 'induce', 'inducement', 'What factors', 'Sun exposure', 'Work and rest', 'In addition', 'among', 'rule', 'Life circle', 'disorder', 'cctv', 'express', 'video', 'expert', 'See', 'together', 'Systematic', 'immune system', 'cause' | Experts said: irregular work and rest, often staying up late, will cause immune system disorders, may induce systemic lupus erythematosus, in addition, what other factors may induce systemic lupus erythematosus? |
| 145 | 37 | 4 | Pemphigus', 'Xin yu'e', 'Quancheng', 'plasma', 'Jinan', 'substitution', 'Brother Yang', 'Cost', 'Official website', 'Behcet ', 'Syndrome', 'follow', 'oral cavity', 'Oral ulcer', 'adsorption', 'lichen planus ', 'nephropathy', 'import', 'Germany' | Jinan Quancheng hospital spent money on the introduction of German plasma exchange equipment geili to carry out "plasma exchange plasma adsorption of blood blue oxygen" to treat autoimmune system diseases, connective tissue diseases, rheumatoid nephropathy and other diseases such as Sjogren's syndrome, pemphigus, pemphigoid, systemic lupus erythematosus, lupus nephritis, rheumatoid arthritis, Behcet's syndrome, recurrent oral ulcer, lichen planus, scleroderma, dermatomyositis, myasthenia gravis, thrombocytopenia, ankylosing spondylitis, psoriasis, purpura, nephrotic syndrome. |
| 146 | 37 | 0 | rose', 'acne', 'Sex dermatitis', 'face', 'Seborrhea', 'erythema', 'distinguish', 'Papule', 'blush', 'Flushing', 'middle part', 'diagnosis', 'dermatitis', 'Pustule', 'ordinary', 'blood capillary', 'Skin lesions', 'Nose', 'skin', 'performance' | Because the skin lesions of rosacea rosea vary in form, especially some facial and ocular symptoms are not typical, it is easy to be confused with other diseases. Some differential diagnoses are collected here for reference. Differentiation between lupus erythematosus and rosacea rabbit lupus erythematosus is an autoimmune disease, which may present with skin lesions similar to rosacea. Lupus erythematosus has the following characteristics: the skin lesions of lupus erythematosus may be erythema or plaque, with deeper color, purplish red color, and clearer boundary; Lack of some inducing factors of paroxysmal flushing and rosacea (such as thermal stimulation, exercise, etc.), and lack of obvious burning sensation and tingling sensation. When the treatment effect of rosacea patients is poor, doctors should consider lupus erythematosus. Differentiation between seborrheic dermatitis and rosacea the clinical manifestations of seborrheic dermatitis may be similar to rosacea, and these two diseases may exist simultaneously. There are two main points for identification: seborrheic dermatitis usually occurs in adolescence, so the age of the patient can be one of the basis for identification; The lesions are often symmetrical, well-defined yellow red patches, and greasy scales can be seen in the nasolabial groove, external auditory canal and eyebrows. Please choose rosacea from the following three pictures. |
| 147 | 37 | 3 | muscle weakness', 'Severe', 'crisis', 'mg', 'muscle', 'Posterior membrane', 'Weakness', 'acetylcholine', 'Involvement', 'cholinesterase', 'achr', 'Hyperactivity', 'adenoma', 'Joint', 'receptor', 'Extraocular', 'swallow', 'Dysphagia', 'thyroid' | What causes myasthenia gravis? Myasthenia gravis is an autoimmune disease. Like other autoimmune diseases, the main cause of the disease is the disturbance of the immune system and the destruction of the normal "order" of the body. Myasthenia gravis is that the acetylcholine receptor on the postsynaptic membrane of neuromuscular junction is attacked by autoantibodies. When the function of this important receptor is abnormal, it will directly affect the normal conduction and action of neuromuscular. The immune system disorder is generally related to environmental factors, infection factors, medication factors, etc., but the specific causes of myasthenia gravis are not clear. Myasthenia gravis patients are often complicated with hyperthyroidism, thyroiditis, systemic lupus erythematosus, rheumatoid arthritis, pemphigus and other autoimmune diseases. |
| 148 | 37 | 2 | Girls', 'Zhang Yang', 'More common in', 'twenty-two', 'can't open one's eyes', 'severe', 'childbearing age', 'Henan', 'woman', 'university student', 'High incidence', 'Cause and effect', 'forty', 'family', 'patent', 'heartbeat', 'To', 'notice', 'consciousness' | December 13, Zhengzhou, Henan Province. In the video, the 22-year-old girl suffered from severe systemic lupus erythematosus. Her heart beat faster and her eyes were swollen. When her family sent her to the hospital, she had lost consciousness and was given a critical illness notice. According to the doctor, the pathogenesis of lupus erythematosus is not very clear, and the high incidence population is mostly seen in women of childbearing age between 15 and 40 years old. Typical symptoms include butterfly shaped erythema, alopecia, ulcer, joint pain, and some people will have morning stiffness of fingers. |
| 149 | 37 | 1 | derivative', 'ii', 'Maleic acid', 'Ether', 'Clinical trials', 'candidate', 'ethic', 'Shanghai Jiao Tong University', 'artemisinin', 'Water solubility', 'examine', 'Chinese Academy of Sciences', 'Stronger', 'The hospital', 'obtain', 'develop', 'Renji', 'Institute', 'Immunosuppression', 'The drug' | The Shanghai Institute of Materia Medica, Chinese Academy of Sciences, obtained artemisinin maleate, a water-soluble artemisinin derivative with stronger immunosuppressive activity, through structural optimization of the natural product artemisinin, which was approved by the State Drug Administration for clinical trials. The new discovery and application has been authorized by two patents at home and abroad, and has completely independent intellectual property rights. Phase II clinical trials will be conducted in patients diagnosed with mild to moderate systemic lupus erythematosus. |
| 150 | 36 | 1 | melatonin', 'sleep', 'insomnia', 'Pineal gland', 'Sunyun', 'awakening', 'Sleeping pills', 'Elf', 'Golden partner', 'improve', 'health foods ', 'take', 'Different', 'fall asleep', 'shorten', 'Before going to bed', 'additive', 'secretion', 'Middle aged and elderly people', 'allow' | Melatonin is an auxiliary sleep, which can shorten the awakening time before going to bed and the time to fall asleep, and improve the quality of sleep. It is not a drug. It is completely different from sleeping pills. Melatonin is a hormone secreted by an elf called pineal gland in the human brain. Different countries have different requirements for melatonin, and China allows it to be used as a health food additive. The only function of products containing melatonin that can be declared and publicized is to improve sleep. However, melatonin can only be used for the middle-aged and elderly people who suffer from insomnia due to the reduction of melatonin, and it is not suitable for pregnant women, lactating women, patients with autoimmune diseases (rheumatism, rheumatoid arthritis, lupus erythematosus, nephritis, etc.), and people with insomnia caused by depressive psychosis |
| 151 | 36 | 5 | Min Wang', 'Ancient costume', 'dance', 'Critically ill', 'Become popular', 'Luzhou', 'girl', 'Release', 'notice', 'Netizen', 'seaside', 'Sichuan', 'Live in', 'Blowing air', 'Hospitalization', 'twenty-one', 'video', 'recrudescence', '' | The real name of the 21-year-old "fat Daodao sister wants to be happy" is Wangmin, a villager in group 5 of Jilong village, Luzhou, Sichuan Province. His parents earn a living by farming in Gulin County on weekdays. Due to lupus erythematosus, Wang Min has been in poor health and has been hospitalized for disease recurrence several times. Previously, she had posted a video of herself dancing in ancient costume on social platforms, and her confident and optimistic attitude infected many netizens. Recently, Wang Min was admitted to the hospital due to the recurrence of lupus erythematosus, and the hospital has issued a notice of critical illness. Wang Min said that her family and netizens gave her a lot of encouragement and help. These comforting words made her feel positive. After leaving the hospital, I wanted to go to the seaside for a hair dryer, because I hadn't seen the sea in 21 years. |
| 152 | 35 | 3 | Fluffy', 'Lingxi', 'Dimly', 'boyfriend', 'Not to kill', 'Liandang', 'Maidservant', 'Cheap fortune teller', 'To show my concubine', 'Throw into the river', 'misfortune', 'die in vain', 'Transfer of ownership', 'Leave one', 'Suicide', 'Two sets', 'London', 'Property', '' | Why do you want to have a baby when you have lupus erythematosus? Don't die?! |
| 153 | 35 | 5 | Shi Shi', 'cornea', 'See the light again', 'sixteen', 'Suzhou', 'donate', 'remains', 'To many', 'light', 'girl', 'Daily application', 'donation', 'left eye', 'Teaching research', 'Antemortem', 'Death from illness', 'fifteen', 'anatomy', 'Gifts' | Shi Shi, a 16-year-old girl in Suzhou, was diagnosed with systemic lupus erythematosus six years ago. In June this year, lupus suddenly recurred, and the complications caused multiple organ failure. On September 30, Shi Shi died of illness. Before her death, she had applied to become an organ donation volunteer. Shi Shi's cornea has helped a 15-year-old boy's left eye to see light again. His body was donated to the medical college for pathology and anatomy teaching research. |
| 154 | 35 | 3 | Flammulina velutipes', 'How to eat', 'broccoli', 'nutritive value', 'Pleurotus ostreatus', 'Avoid food', 'Volvariella volvacea', 'Pleurotus eryngii', 'Spleen and stomach', 'mushroom', 'amino acid', 'lentinus edodes', 'contain', 'recommendation', 'matters needing attention', 'cholesterol', 'Asthenic cold', 'edible', 'content' | Mushrooms in autumn are the most nourishing! These kinds of mushrooms must be eaten Oh 01 Lentinus edodes nutritional value: rich in vitamin D and amino acids that are easily absorbed by the human body, its large amount of glutamate creates a unique taste and flavor. 02 nutritional value of Flammulina velutipes: low in calories and fat, high in protein, vitamins and amino acids. 03 nutritional value of Pleurotus eryngii: it has very high nutritional value, including protein, amino acids, vitamins, carbohydrates, rich dietary fiber, calcium, magnesium, copper, zinc and other minerals. 04 straw mushroom nutritional value: rich in a variety of amino acids and vitamin C, it can promote human metabolism and slow down the absorption of carbohydrates. . 06 nutritional value of Pleurotus ostreatus: Pleurotus ostreatus contains anti-tumor cell polysaccharides, which can improve the body's immunity. Taurine is a component of bile acids, which can dissolve cholesterol, digest and absorb lipids. |
| 155 | 35 | 2 | Wenyifan', 'husband', 'ninety-five', 'image', 'Online loan', 'Antemortem', 'Death', 'Xiongershun', 'forgive', 'Response', 'Yifan', 'Overtemperature', 'Outside', 'Daily sickness', 'didn't come', 'Working', 'Show up', 'Central South', 'WuHan University', 'seventy-five' | On March 6, Wen Yifan, a post-95 mother who suffered from lupus erythematosus, died in Zhongnan Hospital of Wuhan University 75 days after struggling with the disease. She released a video before her death, but still didn't forgive her husband, and still asked whether he had been here. Wenyifan's husband had previously appeared to respond, saying that he "returned the online loan and car loan in order to pay back the money.". |
| 156 | 34 | 0 | Nihaisha', 'On the station', 'NI SHI', 'For this', 'Confucian School', 'origin', 'heart', 'teacher', 'Traditional Chinese Medicine', 'Prescription', 'video', 'White jade', 'bilibili', 'gentleman', 'Tangqu', 'Albino', 'textual research', 'Menstrual leave', 'Famous doctor' | There is teacher nihaixia at station B. If you need help with the cause and treatment of this disease, you must see it |
| 157 | 34 | 3 | Lung qi', 'large intestine', 'Deficiency', 'Lung regulating qi Division', 'Lung governing', 'Adenoid body', 'Resuscitation', 'Sex pharynx', 'Ying Wei', 'peristalsis', 'coat', 'Exterior interior', 'Qi of', 'nasal polyp', 'nasosinusitis', 'Exopathogens', 'Nasal congestion', 'hypertrophy', 'pharyngitis' | Insufficient Lung Qi can lead to: 1. The lung and the large intestine are in the same exterior and interior. Insufficient Lung Qi can not peristalsis the large intestine, so it will lead to constipation. 2: Lung resuscitation in nose: rhinitis, sinusitis, nasal polyps, nasal congestion, adenoid hypertrophy. 3: The lung governs Qi and is responsible for breathing, respiratory problems, cough, pharyngitis, cough and asthma, bronchial and tonsillar inflammation, herpetic pharyngobuccal inflammation. 4: Lung dominates fur, vitiligo, psoriasis, lupus erythematosus, urticaria, eczema, etc . Good spleen and stomach, sufficient Lung Qi! |
| 158 | 34 | 1 | Tongtong', 'encephalopathy', 'Psychosis', 'Mental illness', 'neck', 'Lines', 'Houtongtong', 'Pinch', 'Check', 'Insanity', 'In class', 'Villain', 'lupus', 'Cognitive impairment', 'classmate', 'Unprovoked', 'forgetful', 'both cheeks', 'local' | Tongtong, a 15-year-old girl, talked to herself in class and told her teacher that she saw a group of little people dancing in the classroom. At first, the head teacher thought these were "tricks" of troubled students to make trouble in class. The teacher didn't realize the seriousness of the problem until Tongtong grabbed the neck of the class for no reason and said the lines in the animation. Tongtong's family took him through a series of evaluations and was diagnosed as "acute and transient psychotic disorder". Soon after, Tongtong developed a high fever, and the examination also found the presence of pleural effusion. Her parents rushed her to a tertiary hospital for treatment. The doctor found a red rash on Tongtong's cheeks and nasal bridge, like a butterfly with wings on his face. The doctor said that the child had lupus encephalopathy instead of mental illness. At present, after active immunotherapy and the assistance of relevant departments, Tongtong's mental symptoms gradually. |
| 159 | 34 | 0 | Lumbago and leg pain', 'ulcer', 'old age', 'Dosage', 'Brain canal', 'syndrome', 'Mental worker', 'Obstacle', 'Tonic peptide', 'Cerebral hemorrhage', 'fracture', 'Frail person', 'Child intelligence', 'Detoxification', 'chronic', 'However, it should', 'gynaecology', 'Painstaking efforts', 'renal function', 'colon' | Who needs peptide supplements? People with the following diseases should take peptides more: 1. patients with diseases that are difficult to absorb and lose efficacy on drugs for a long time. 2. mental workers and weak people; 3. develop children's intelligence, but should be halved; ; 5. those who want to quit drugs, alcohol and smoking should increase the amount by 1/2; 6. patients with renal dysfunction can promote the recovery of renal function, but the dosage cannot be increased; 7. various inflammation: cold, tuberculosis, tumor, lupus erythematosus, gynecological chronic inflammation, etc; 8. digestive system diseases: fatty liver, early liver cirrhosis, chronic gastritis, duodenal ulcer, colon ulcer, constipation; 9. fracture convalescence, osteoporosis, rheumatism, rheumatoid arthritis, chronic waist and leg pain in the elderly; 10. cardiovascular and cerebrovascular diseases, such as cerebral infarction, cerebral hemorrhage, cerebral thrombosis, autonomic nervous dysfunction syndrome; 11. type II diabetes mellitus, wound healing, anemia, leukemia, patient recovery period; ; 13. male and female sexual function and physical dysplasia. 14. the fetal image of pregnant women is unstable. People in special need: cancer, tumor, blood loss, scald, anemia, fracture, burn, fatigue, cold, pregnant women, surgery, weight loss, children, hypertension, diabetes, the elderly, athletes, gastrointestinal ulcer, malnutrition. |
| 160 | 33 | 5 | diamond', 'Mom', 'Zhouyushan', 'Filial piety', 'Subsidize household', 'Vigorously', 'Drop out', 'juvenile', 'look after', 'Far away', 'Besides', 'Live on campus', 'away from home', 'Most beautiful', 'overcome', 'shoulder', 'back', 'go to school', 'junior high school', 'school' | A 13-year-old girl with a sick mother painted a "Diamond painting" to supplement her family. Zhouyushan's mother suffers from lupus erythematosus. Besides taking care of her mother, she also makes diamond paintings to ease the difficulties at home. For a long time, she had back pain, shoulder pain and myopia, but for her mother's sake, she tried to overcome it. Junior high school has to go to a school far away from home. She can only live on campus. In order to take care of her mother, she even thought of dropping out of school and going home. |
| 161 | 33 | 1 | Qiao Yu', 'Zhoujingze', 'Jingze', 'full text', 'diary', 'Really', 'That's all', 'Send a', 'At that moment', 'Look', 'public', 'that day', 'case', 'Piercing', 'break up', 'Heart', 'ruthless', 'notice', 'No. jade Mang' | God seems to have made a huge joke with me. At the moment when I got the report, my sky seemed to collapse. What should I do... "They all said that this disease is called immortal cancer. I am really afraid. Life with kyungwa is just getting better. Why does fate make such a joke with me?" |
| 162 | 33 | 0 | immunity', 'Repression', 'Lack of sleep', 'Two characters', 'equation', 'work', 'abuse', 'health products', 'strong', 'habit', 'disseminate', 'Get sick', 'antibiotic', 'actually', 'great', 'balance', 'protect', 'contract' | "Health = less illness = strong immunity", such an "equation" is often used in the promotion of health products. In fact, the most important thing for the immune system to function properly is the word "balance". Abuse of antibiotics, lack of sleep, depression, etc. will reduce immunity, while the following good habits will protect immunity. |
| 163 | 32 | 4 | finger', 'Whitish', 'Reynolds', 'Miss Wang', 'Sometimes', 'Cold', 'Jiangsu', 'Changzhou', 'Supercooled', 'Syndrome', 'Miss Li', 'Purplish', 'cooling', 'fifty-three', 'connective tissue', 'recently', 'winter', 'woman' | Doctor: it's not skin disease. It's the immune system. Recently, Ms. Wang, 53, from Changzhou, Jiangsu Province, found that as long as she cooled down, her fingers would change color, sometimes white and sometimes purple, her joints were also painful and swollen, and she also had chest tightness and cough. The doctor found that this was because Ms. Wang had an immune system disease: connective tissue disease, which caused Raynaud's syndrome. Connective tissue diseases mainly include lupus erythematosus, rheumatoid, Sjogren's syndrome, etc. Doctors reminded that many people thought that when Raynaud's syndrome appeared, it was just cold weather, and they came to the hospital after a long time with joint pain, muscle weakness and other symptoms, which would affect the treatment effect and even endanger life. |
| 164 | 32 | 0 | O'Connor', 'Flannery', 'United States', 'south', 'Hard to find', 'writer', 'literature', 'novel', 'novelist', 'Grotesque', 'Literary circles', 'critic', 'Scholarly fragrance', 'Good people', 'works', 'peacock', 'Thirty nine years old', 'National Book Award', 'thirty-nine' | Ted Guide: famous American writer Flannery O'Connor bilingual subtitle Mary Flannery O'Connor, American novelist, short story writer and critic, and an important spokesman of American literature. She has written two novels, 32 short stories and a large number of book reviews and film reviews. O'Connor is a Southern writer. Her works have Southern Gothic style and rely on regional background and grotesque characters. She was the first American female writer to be listed in the authoritative "American Library" after World War II. O'Connor never married. She spent most of her time on the farm with her mother. At the age of 39, she died of familial hereditary lupus erythematosus. |
| 165 | 32 | 3 | appropriation', 'Xiaojinlin', 'five hundred and ninety-three', 'eighty-six thousand six hundred and fifty-one', 'Hanjiang District', 'two thousand and eighteen', 'Project approval', 'Yuwanyuan', 'transport', 'Person time', 'one hundred and ninety', 'Total', 'total', 'second half', 'Yangzhou', 'expenditure', 'Solicit donations', 'two thousand and fourteen', 'clj ', 'writer' | Discovered by professionals. 9958 cooperated with an acupuncture and moxibustion hospital in Hanjiang District, Yangzhou, and delivered a total of 593 patients with ankylosing spondylitis, lupus erythematosus, and psoriasis (August 2014 June 2018), with a total appropriation of more than 8.6651 million yuan. Xiaojinlin is one of them. In the second half of 2018, it changed its name and re approved the project. It raised 1.9 million yuan a year. There was no appropriation record in the publicity. |
| 166 | 32 | 4 | Tripterygium wilfordii', 'lactone', 'contraception', 'masculine', 'contraceptive', 'research', 'Male rat', 'traditional Chinese medicine', 'Rheumatic arthritis', 'have', 'toxicity', 'sperm', 'preparation', 'extract', 'The book', 'Hormones', 'find', 'rheumatism', 'male', 'Alpha' | Tripterygium wilfordii polyglycoside, a slow acting anti rheumatic drug, is a very important drug in the Treasury of traditional Chinese medicine, especially in the treatment of rheumatoid arthritis, systemic lupus erythematosus and other rheumatic diseases. The mechanism of Tripterygium wilfordii polyglycosides has been gradually confirmed by medical scholars. However, because Tripterygium wilfordii polyglycoside is a plant extract, its composition is related to the planting area of Tripterygium wilfordii, resulting in different effects of Tripterygium wilfordii polyglycoside extracted from different regions. The usual dose of Tripterygium wilfordii is 20mg, three times a day, which can be tolerated by most patients, and its curative effect is also positive. |
| 167 | 32 | 1 | misunderstanding', 'actually', 'lupus', 'infectivity', 'Heritability', 'side effect', 'Half a year', 'more than', 'Stop using', 'Liusijia', 'Several major', 'Medication treatment', 'clarify', 'Give birth to a baby', 'xiangya hospital ', 'know', 'Central South University', 'medicine', 'High risk', '' | Liusijia, a doctor in the Department of Rheumatology and immunology of Xiangya Hospital of Central South University, would like to talk about some misunderstandings many people have about systemic lupus erythematosus and clarify them: ① the rash and erythema of lupus patients on the face and hands are infectious. In fact, these skin lesions are not infectious. Misunderstanding ② hormone drugs have long-term side effects. In fact, the drug has only temporary side effects. Long term psychological preparation should be made for the drug treatment of lupus. Never leave the drug treatment because the drug has side effects. Misunderstandings ③ lupus patients cannot have babies. In fact, the patient's condition was stable for more than half a year, the hormone dose remained below one and a half tablets, and the use of other drugs affecting the fetus was stopped for more than half a year. Misunderstanding ④ lupus is a genetic disease. In fact, lupus is only a genetic predisposition, but it is not a genetic disease. |
| 168 | 31 | 1 | Zhouzhiruo', 'So old', 'poor', 'unmanned', 'Present cause', 'difficulty', 'goddess', 'life', 'Headline', 'article', 'release', 'Most beautiful', 'Bijiuyin', 'White bone', 'Take away', 'Zhuyan', 'Most', 'Hanfeifei', 'To pacify the elderly', 'Mirror Flower' | "Zhou Zhiruo" is really old and has difficulty in living with "lupus erythematosus"; today, because of poverty, there is no one to catch up with " |
| 169 | 30 | 3 | Counter offensive', 'plasmodium', 'Drug resistance', 'Call', 'Call and release', 'Military University', 'Human history', 'plant', 'gentleman', 'achieve', 'efficacy', 'Renshijun', 'Big move', 'Not only', 'cita', 'artemisinin', 'Xiaodou', 'secsoul', 'cc', 'amber' | The big move has been released. Artemisinin has not only achieved the counterattack against drug-resistant Plasmodium, but also has a new effect on lupus erythematosus. Call Mr. Tu youyou! |
| 170 | 30 | 0 | fever', 'temperature', 'Low fever', 'Low fever', 'fever', 'infant', 'Infectivity', 'baby', 'children', 'Heat dissipation', 'See', 'long-term', 'infect', 'Shiver', 'convulsion', 'reason', 'cooling', 'Hyperthermia', 'Thermogenesis' | General medical knowledge: how to deal with fever caused by common diseases the teacher's Shanghai friend said that they are currently strictly prevented and controlled, and they all choose to live in isolation or work at home. The food at home will be delivered by the community. What should we do if there is fever during home? |
| 171 | 29 | 3 | help seeker', 'pbobeerbanma', 'This', 'Children', 'Mom and Dad', 'Help', 'grandpa', 'Cripple', 'Help', 'Valiantly', 'Roasted bran', 'Disorderly meal', 'Pleurotus', 'Listen', 'knife', 'twelve', 'Mom', 'Milk candy', 'Sweet black' | Help the family. My father, mother and grandfather are infected. My mother also has lupus erythematosus. The person asking for help is a 12-year-old child. Help me!!! |
| 172 | 29 | 2 | Faint', 'Zhou Haimei', 'Etiology', 'Female artist', 'screen', 'total assets', 'rest', 'Deceased', 'Unexpected', 'Unprovoked', 'too low', 'wake', 'Heart', 'Relatives', 'image', 'Hong Kong', 'appear', 'Good health' | . She said that when she was a child, her platelets were too low and she fainted for no reason. She did not find out the cause. Her relatives and friends around her could not imagine that she would not wake up after fainting because of this disease. Zhou Haimei is a Hong Kong female artist loved by the Post-70s, post-80s and post-90s. Her screen image will still be in our hearts. Finally, may the deceased rest in peace. |
| 173 | 29 | 3 | women's soccer team', 'You Yiting', 'Shanghai', 'Vital', 'u18', 'Team member', 'Life and death test', 'Unbearable', 'everybody', 'Make every effort', 'Flowering season', 'Huge', 'Give', 'Jianmusheng', 'Writing', 'coach', 'Medical expenses', 'Wither', 'dear' | Dear friends, you Yiting, a member of the Shanghai women's football team, suffered from systemic lupus erythematosus and was in critical condition. The 18-year-old young girl was facing the test of life and death. Her family also made every effort to provide assistance for her children, but the huge medical expenses were beyond the ordinary family's bearing. I hope you kind-hearted people can give a little love and don't let this young life wither. Thank you |
| 174 | 29 | 3 | anger', 'emotion', 'negative', 'hostility', 'easily', 'Grief', 'Repression', 'resentment', 'hate', 'forgive', 'stress', 'coronary artery', 'Dislocation', 'anxious', 'forgive', 'prolapse', 'call', 'others', 'obtain' | There is a book called Deadly Emotions, written by Don Colbert, a licensed family physician in the United States. In his book, he cited many studies on the relationship between emotion and disease in recent years. For example, people who are often angry and hostile are prone to suffer from hypertension and coronary artery disease; Resentment, grief, inability to forgive and forgive others, self hatred, easily lead to autoimmune system diseases, such as rheumatoid arthritis, lupus erythematosus and multiple sclerosis; Anxiety, easy to cause stress intestinal syndrome; Panic attack, leading to mitral valve prolapse; Suppressed anger can easily cause tension, migraine, chronic low back pain, dislocation of mandibular joint, fibromyalgia syndrome, etc. When we suppress some negative emotions, they will be expressed through the body! Every disease is the call of the body, which calls for attention, affirmation, gratitude and acceptance. A lot of sadness, fear, anger, dissatisfaction, and complaints stem from not getting what the body needs or wants. Disease is an inevitable part of life. Modern medicine has no cure for many diseases. Hypertension, cardiovascular disease, diabetes, various cancers, AIDS, and various immune deficiency diseases can only remain relatively stable, while the side effects of drugs are great. |
| 175 | 29 | 0 | Cyanine', 'Brown algae', 'Rigidity', 'Spondylitis', 'asthma', 'Ectopic', 'rhinitis', 'balance', 'Allergic', 'dermatitis', 'urticaria', 'allergy', 'Excesses', 'supreme', 'New Zealand', 'candy', 'health', 'Arthritis', 'immunity', 'Qingsu tablet' | ♀ All inflammations are immune problems rhinitis, nasal allergy, asthmatic arthritis, ankylosing spondylitis, allergic dermatitis, urticaria, lupus erythematosus, atopic dermatitis, and so on -- we must take brown algal astaxanthin |
| 176 | 29 | 2 | Bask in the sun', 'No', 'Wolf friend', 'Total healing', 'sunlight', 'medical matters', 'patient', 'sensitive', 'outdoor', 'absolute', 'Light', 'Consulting room', 'Drips', 'View', 'Large window', 'appropriate', 'Flare', 'Popular science', 'stifling' | Why can't lupus patients sunbathe? |
| 177 | 29 | 2 | Sunbathing', 'woman', 'diagnose', 'Sunscreen', 'Bask in the sun', 'prepare for one's old age', 'Claim to have', 'Four seasons of the year', 'Frightening', 'Sanya', 'My Regiment', 'Don't sunburn', 'induce', 'Fujian', 'Photoaudiometry', 'Tenthousand times', 'Uncoated', 'Raise a child' | The key is that many people don't know they have this disease. It's too late to know until they get sick |
| 178 | 28 | 4 | Anomala', 'Xinwozi', 'official', 'Fancy', 'Chenfang', 'Ouxi', 'Practice', 'For injection', 'micro-blog', 'recombination', 'attention', 'fuse', 'receptor', 'Zongzhaoji', 'thanks', 'factor', 'lymphocyte' | I would like to know what medicine is used to treat lupus erythematosus? |
| 179 | 28 | 3 | Thymosin', 'technology', 'pd1', '00', 'fermentation', 'express', 'affinity ', '000', 'pdl2', 'pdl1', '0c', 'Specificity', 'hydrolysis', 'Blockers', 'Inactivation', 'Activator', 'minute', 'regulator ', 'extract', 'activation' | 《Secondary acidic thymosin /ph=6/ Bacillus subtilis / ethanol free / intracellular non fusion expression /800000 iu/g/ anhydrous》 |
| 180 | 28 | 5 | disaster', 'General', 'miracle', 'Incurable disease', 'encounter', 'recovered', 'contract', 'time', 'skin', 'fund', '878', 'Zhuyilong', '559', '898', '651', '868t', '268', 'Brilliant blue', 'three hundred and eighty-nine', 'three hundred and twenty-one' | I was suffering from the terminal skin disease lupus erythematosus. I met him at the most critical moment and he helped me recover miraculously. |
| 181 | 27 | 2 | the people', 'direct seeding', 'lecture', 'theme', 'Immunology Department', 'Zhengzhou', 'Be a guest', 'Zheng Yi', 'Free clinic', 'Live broadcasting room', 'lupus', 'world', 'rheumatism', 'director', '17001800', 'place', 'education', 'invite', '10', 'Chief physician' | What kind of disease is lupus erythematosus? Do you really know? From 17:00 to 18:00 on January 12, Chen runyun, director of Rheumatology and Immunology Department of Zhengzhou people's Hospital, and Han Dan, deputy chief physician of rheumatology department of Zhengzhou people's hospital were invited to the live broadcast room to focus on "what kind of disease is lupus erythematosus? Do you really understand it? "This topic is for everyone to answer questions. |
| 182 | 27 | 3 | Green Valley', 'cfda', 'pharmacy', 'gv971', 'Easy to see', 'Cheat', 'Substitutes', 'Low quality', 'polyphenol', 'Fifth', 'rough', 'Scam', 'Join forces', 'away from', 'Traditional Chinese Medicine', 'Third', 'control group', 'Chinese Academy of Sciences', '' | The first gv-971 is obviously easy to see low-quality clinical scam like, CFDA "conditionally approved" Green Valley pharmaceutical; The fifth artemisinin "treatment of lupus erythematosus" is only an immunosuppressant substitute. The effect of the rough animal experiment 20 years ago is not as good as that of the control group |
| 183 | 27 | 2 | mandible', 'How's it going', 'Molar bone', 'Word of mouth', 'self-help', 'Which one', 'picosecond', 'query', 'Guangzhou', 'resection', 'Chongqing', 'Often ask', 'hospital', 'Freckle removal', 'Osteotomy', 'hypertrophy', 'on line' | The best hospital for systemic lupus erythematosus in Chongqing. |
| 184 | 27 | 5 | Lines', 'neck', 'classmate', 'encephalopathy', 'girl', 'diagnose', 'lupus', 'Zhaojia', 'Induction', 'consciousness', 'First time', 'think', 'Idea', 'Female classmate', 'dreadful', 'sober', 'young', 'Party members', 'Hong Kong Island', 'Scattered regression' | I thought lupus erythematosus was a skin disease |
| 185 | 27 | 5 | Hong Kong wide', 'fifty-three', 'Rich merchant', 'Red carpet', 'Men', 'Infatuation', 'lover', 'Hong Kong', 'Return to the Jianghu', 'Marriage glamour pressure', 'abandon', 'bearing', 'Still gorgeous', 'Dare not', 'entertainment', 'fifty-two', 'Headline', 'Lvliangwei', 'article', 'ideal' | "She is the ideal type for all men in Hong Kong. She is afraid to have children when suffering from lupus erythematosus. She is not married at the age of 53." |
| 186 | 27 | 3 | Yangxichuan', 'perchloric acid', 'Dermatology', 'chirality', 'Isomer', 'Sweetening', 'professor', 'acupuncture', 'Murder for money', 'doctor', 'andersguo', 'laruence', 'once', 'disciple', 'Pleasure', 'Craty', 'Hippo', 'Chestnut', 'the nine degrees of kinship', 'Liuweizhong' | What??? fuck？ Acupuncture treatment of lupus erythematosus? |
| 187 | 27 | 4 | Concord', 'Beijing', 'Peking Union Medical College Hospital', 'Yes no', 'doctor', 'Hang up', 'Too hard', 'review', 'registration', 'cattle', 'Ask questions', 'next time', 'Who', 'Friends', 'Registration fee', 'Systematic', 'Beijing hospital', 'doctor', 'recommendation', 'sister' | I want to go to Peking Union Medical College, please? |
| 188 | 26 | 3 | get angry', 'Sulk', 'anger', 'the old', 'Not satisfied', 'Inflamed', 'character', 'speak', 'right', 'be more competitive', 'annoying', 'Contradict', 'left', 'others', 'Sad', 'work', 'Lumbar disc', '' | Generally, girls get sick because their mother or father is angry with their biological parents. Boys get sick because their parents are angry with non consanguineous parents. For example, mothers are angry with their in laws and fathers are angry with their in laws. |

**Table S4. Mean Sentiment Across Groups**

Mean of the average sentiment score for all groups by Weibo and Reddit. Values closer to -1 reflect negative sentiment, values closer to 0 reflect neutral sentiment, and values close to 1 reflect positive sentiment.

| **Group** | **Mean Sentiment（SD）** | **95% CI** |
| --- | --- | --- |
| Reddti | 0.42 (0.86) | 0.40, 0.43 |
| Navigating Lupus Diagnosis and Daily Struggles | 0.44 (0.86) | 0.42, 0.46 |
| Skepticism and Sarcasm in Alternative Health | 0.45 (0.85) | 0.41, 0.49 |
| High-Risk Pregnancy and Infant Medical Journeys | 0.38 (0.86) | 0.31, 0.45 |
| Everyday Life & COVID Precautions | 0.38 (0.90) | 0.32, 0.44 |
| Photosensitivity and Visible Symptoms in Autoimmune Disease | 0.29 (0.91) | 0.24, 0.34 |
| Struggles with Autoimmune Medications and Treatment Hesitancy | 0.43 (0.86) | 0.38, 0.48 |
| Weibo | 0.01 (0.91) | 0.00, 0.02 |
| Immunity and Yang Chi Regulation from a Chinese Medicine’s Perspective | -0.25 (0.94) | -0.27, -0.23 |
| Daily Life and Emotional States of Lupus Patients | 0.22 (0.83) | 0.21, 0.23 |
| Inspirational Stories of Fighting Illness | -0.29 (0.83) | -0.25, -0.21 |
| Everyday Herbal Health Advice and Recommendations | 0.05 (0.97) | 0.02, 0.08 |
| Basic Knowledge and Diagnostic Confusion in Rheumatic Diseases | 0.04 (0.87) | 0.00, 0.08 |
| Emotional Support and Family Bonding During Illness | -0.06 (0.95) | -0.09, -0.03 |

**Table S5. Parameter Settings of GPT-5 Thinking**

| Parameter | Value |
| --- | --- |
| Temperature | 0 |
| Top-p | 1 |
| Seed | 42 |
| Presence penalty | 0 |
| Frequency penalty | 0 |
| Reasoning effort | High |
| Maximum output tokens | 4096 |
