# Supplementary material for: AI-Driven Topic Modeling and Sentiment Analysis of Systemic Lupus Erythematosus Discussions on Social Media: Cross-Platform Study
Source: J Med Internet Res. 2026 Aug 31;28:e95175. doi: 10.2196/95175 (PMC13528885; doi:10.2196/95175)
Supplement: Supplementary file 2 [file jmir-v28-e95175-s002.docx]

**Table 1 Operational definitions of sentiment categories**

| **Category** | **Definition** | **Examples of linguistic markers** |
| --- | --- | --- |
| Positive | Posts expressing favorable attitudes, hope, gratitude, improvement, reassurance, or constructive engagement toward the topic | hope, grateful, better, improving, thankful, supportive |
| Negative | Posts expressing unfavorable attitudes, fear, frustration, pain, dissatisfaction, anxiety, or criticism | scared, painful, frustrated, hopeless, angry, worried |
| Neutral | Posts containing factual statements, informational queries, or objective descriptions without clear emotional valence | questions about symptoms, medication names, diagnostic results, factual descriptions |
