# Supplementary material for: AI-Driven Topic Modeling and Sentiment Analysis of Systemic Lupus Erythematosus Discussions on Social Media: Cross-Platform Study
Source: J Med Internet Res. 2026 Aug 31;28:e95175. doi: 10.2196/95175 (PMC13528885; doi:10.2196/95175)
Supplement: Supplementary file 3 [file jmir-v28-e95175-s003.docx]

**Table 2 Overview of Groups of Topics With Example Text**

| Group | Name | Topic No. | No. | Definition | Keywords | | Example text |
| --- | --- | --- | --- | --- | --- | --- | --- |
| Reddit | | | | | | | |
| 0 | Navigating Lupus Diagnosis and Daily Struggles | 0,1,3,4,11,12,14,21,29,36,40,41,44,45,46,55,74,76 | 6084 | Focuses on lupus patients’ diagnostic experiences, emotional support, and hope for the future. | | 'thank', 'appointment', 'ask', 'great', 'know', 'rheum', 'told', 'aches', 'also', 'aunt' | Yep, I developed the burning tongue also. Anything with the slightest spice or hot (temperature) will trigger it for hours. Cold soothes it for me.  I started experiencing this years after my diagnosis which is weird. I'm still seeking a diagnosis as I was just dropped by my rheumatologist because my numbers weren't high enough for him. It's still positive, but not bad enough for him.  It took me months to finally be told I had lupus, and at one point I was taking medication I shouldn't have all because they didn't understand. So I know how frustrating this is. |
| 1 | Skepticism and Sarcasm in Alternative Health | 15,17,24,26,27,31,34,35,37,47,48,49,51,54,56,58,59,64,66,67,68,69,70,71,72,73,79,80,81,85,91,95,96 | 1491 | Includes sarcastic and skeptical discussions about alternative health remedies like celery juice and sea moss. | | 'diet', 'eating', 'foods', 'infection', 'loss', 'cure', 'food', 'research', 'antiphospholipid', 'aps' | Is it possible to have a Beta 2 Glycoprotein Deficiency? If so, what does it mean? Anyone have any ideas?  But what about the celery juice? Did you try shoving it up ur ass? If not, then unlikely to work. Yeah, rookie mistake!!Omg they always talk about the stupid celery juice lol! And it’s gotta be “cold pressed” or else it kills the cells or whatever. |
| 2 | High-Risk Pregnancy and Infant Medical Journeys | 18,25,28,30,42,43,62,78,82,90,92,97 | 592 | Centers on high-risk pregnancies and neonatal medical challenges like transfusions and rare conditions. | | 'pregnancy', 'postpartum', 'underlying', 'transfusion', 'born', 'hormones', 'want', 'gaslighting',' undiagnosed', 'questions' | Side note: has anyone felt better when pregnant? I thought it was so strange that when I got pregnant my depression went away, my heartburn/reflux stopped, my joints didn’t ache and my skin looked great and I had tons of energy. It’s usually the opposite so I started being suspicious something weird was up with my body  I’ve never had a baby but, my mom had lupus while pregnant with me(she past away when i was 14) and i was a small baby. i was 5 pounds 8 ounces and a month and a half early |
| 3 | Everyday Life & COVID Precautions | 9,10,13,16,20,75,77,84,86,88,93,94,98 | 850 | Posts reflecting on daily routines, home or work life, and discussions of mask-wearing and vaccination during the pandemic era. | | 'covid', 'home', 'job', 'mask', 'school', 'sick', 'work', 'amazon', 'ice', 'kids' | Then I got boosted and then masking became less important when I broke my leg from a massive seizure from lupus 3 years ago and I have barely left the house. But to be told I am killing people and that my anecdotal evidence is being touted as fact by you this is just getting ridiculous  O, those symptoms are not typical lupus symptoms. Even if they started sometime after my second Pfizer COVID shot and haven’t gone away? It’s the worst exhaustion I’ve ever felt and it’s getting in the way of me getting things done. |
| 4 | Photosensitivity and Visible Symptoms in Autoimmune Disease | 2,5,19,23,33,39,53,61 | 1216 | Discusses visible symptoms like rashes and sun sensitivity in autoimmune diseases such as lupus. | | 'skin', 'sun', 'rosacea', 'sunscreen', 'car', 'derm', 'heat', 'looks', 'makeup', 'malar' | I highly suspect that this is a malar rash. Basic blood work indicates autoimmune condition, doctors ignore this and say further testing will just cause "health anxiety"  I’m thinking I need to avoid the sun like a vampire lol. Avoiding sun like the vampire is oddly difficult. |
| 5 | Struggles with Autoimmune Medications and Treatment Hesitancy | 6,7,8,22,32,38,50,52,57,60,63,65,83,87,89 | 1085 | Focuses on experiences, fears, and side effects related to medications for autoimmune conditions such as UCTD and early lupus. | | 'mg', 'plaquenil', 'dose', 'medication', 'meds', 'methotrexate', 'acid', 'benlysta', 'colonoscopy', 'hcq' | Also check what you taking (fluid, food, other meds, etc) along with HQC. I learned the hard way that HCQ doesn’t play well with stimulants (especially adhd meds) or anti-histamines.  It was poisoning me. I tried Cellcept. After three years I was finally approved for benlysta. Oh god I cannot imagine how hard it was for you to get by until you found the medication that worked for you. |
| Weibo | | | | | | | |
| 0 | Immunity and Yang Chi Regulation from a Chinese Medicine’s Perspective | 6,17,19,25,26,29,31,37,38,43,49,52,66,68,74,75,76,82,84,90,95,96,99,101,105,106,115,117,118,139,143,144,146,156,159,162,164,170,175 | 5562 | Focuses on traditional Chinese medicine concepts like immunity, yang energy, and qi transformation, often recommending moxibustion. | | 'immunity', 'dermatitis', 'function', 'Yang Chi', 'cause', 'balance', 'sub-health', 'traditional chinese medicine', 'social circle', 'hyperthyroidism' | When patients with lupus erythematosus become pregnant, their platelet count is below the normal range. After taking the modified Angelica Decoction for one month, it returned to the normal level. Then, it was continued to be taken until the 6th month of pregnancy. Currently, it is 8 months pregnant and the blood test results are normal. |
| 1 | Daily Life and Emotional States of Lupus Patients | 0,2,3,5,7,12,13,16,18,20,23,24,27,36,39,45,46,56,61,64,69,70,77,78,83,86,87,97,102,103,107,110,111,130,136,137,140,141,149,150,158,161,167,168 | 12920 | Documents the emotional fluctuations and life experiences of lupus patients, including symptoms like insomnia and proteinuria. | | 'invasion', 'monoclonal antibody', 'severe', 'reimbursement', 'terrible', 'hope', 'insomnia', 'is there', 'feeling', 'monologue' | She developed lupus encephalitis. She lost her ability to recognize her family members and stopped eating. The family members requested to take her home for treatment. A local hospital acquaintance said that she hadn't eaten for three days and three nights. Her husband had given up.  The immune system within my body is now attacking itself, causing me unbearable pain and making me feel as if I'm no more than a lifeless ghost. |
| 2 | Inspirational Stories of Fighting Illness | 4,10,11,15,28,48,50,53,54,73,88,92,94,100,109,116,126,127,148,155,172,176,177,181,183 | 4792 | Tells motivational stories of individuals (especially women) . | | 'Zhou Haimei', 'sunbathe', 'pass away', 'prefer', 'college student', 'healthy', 'childbearing age', '40', '57', 'Jackson' | My wife had her second check-up the other day. It was confirmed that she has developed systemic lupus erythematosus nephritis. It's been exactly one month since then. During the first check-up, all the indicators were on the rise, and the medication has not been missed at all under my urging. Everything is moving in a positive direction.  Now I've realized...It turns out that lupus erythematosus is so terrifying. But I'm not afraid. For three years, the fear may have already vanished, or perhaps there is light in my heart! |
| 3 | Everyday Herbal Health Advice and Recommendations | 8,9,21,22,30,32,41,47,51,57,59,62,63,65,67,71,79,81,85,89,91,98,108,119,120,121,122,123,124,125,129,131,132,134,138,142,147,152,154,157,165,169,171,173,174,179,182,186,188 | 5464 | Presents daily health tips using herbs like chrysanthemum, hawthorn, and yam for general wellness and self-care. | | 'fans', 'three', 'No.2', 'Jiajia', 'vicious', 'operation', 'princess', 'poria cocos', 'myopia', 'acupuncture' | When going out for exercise, it is important to protect yourself from the sun. You can use a sunshade umbrella, apply sunscreen, wear a hat, and also wear long-sleeved shirts and pants for protection. Additionally, you should take good care of your skin and mucous membranes. Avoid using certain cosmetics and be especially cautious about not using harsh soaps or bath liquids.  In an environment with strong sunlight, ultraviolet rays can enhance the antigenicity, thereby triggering the occurrence of lupus erythematosus. |
| 4 | Basic Knowledge and Diagnostic Confusion in Rheumatic Diseases | 14,33,42,60,72,80,93,113,114,128,133,145,163,166,178,187 | 1576 | Discusses confusion about rheumatic diseases like ankylosing spondylitis and rheumatoid arthritis, often involving user consultations. | | 'syndrome', 'rheumatism', 'dryness', 'connective tissue', 'rheumatoid arthritis', 'joint', 'friends', 'factor', 'check in', 'nursing' | it should be distinguished from arthritis caused by osteoarthritis, gouty arthritis, reactive arthritis, psoriatic arthritis and other connective tissue diseases  The difference between rheumatism and rheumatoid is not a concept. |
| 5 | Emotional Support and Family Bonding During Illness | 1,34,35,40,44,55,58,104,112,135,151,153,160,180,184,185 | 3314 | Centers on the role of mothers, children, and family in emotional expression and support during illness. | | 'girl', 'child', 'give birth', '13', 'think', 'children', 'decide', 'donate', 'time', 'mother' | Hug you, cheer up, think more about the happy things. There are many lupus patients like you by your side. We are all with you. Even if we are not by each other's side, I promise I will take good care of myself.  Sister wants to take you home, not letting systemic lupus erythematosus claim your life.  Don’t let refractory systemic lupus erythematosus take my mother away |
